# Discovery of Oral Degraders of the ROS1 Fusion Protein with Potent Activity against Secondary Resistance Mutations

**DOI:** 10.1021/acs.jmedchem.4c01205

**Published:** 2024-10-03

**Authors:** Xianyou Peng, Shanchun Guo, Shilong Zheng, Ahamed Hossain, Changde Zhang, Madhusoodanan Mottamal, Elena Skripnikova, Peng Ma, Kindy Martinez-Carter, Qiang Zhang, Faisal Abedin, Thomas Huckaba, Guangdi Wang

**Affiliations:** † Department of Chemistry, Xavier University of Louisiana, New Orleans, Louisiana 70125, United States; ‡ RCMI Cancer Research Center, Xavier University of Louisiana, New Orleans, Louisiana 70125, United States; § College of Pharmacy, Xavier University of Louisiana, New Orleans, Louisiana 70125, United States; ∥ Department of Biology, Xavier University of Louisiana, New Orleans, Louisiana 70125, United States

## Abstract

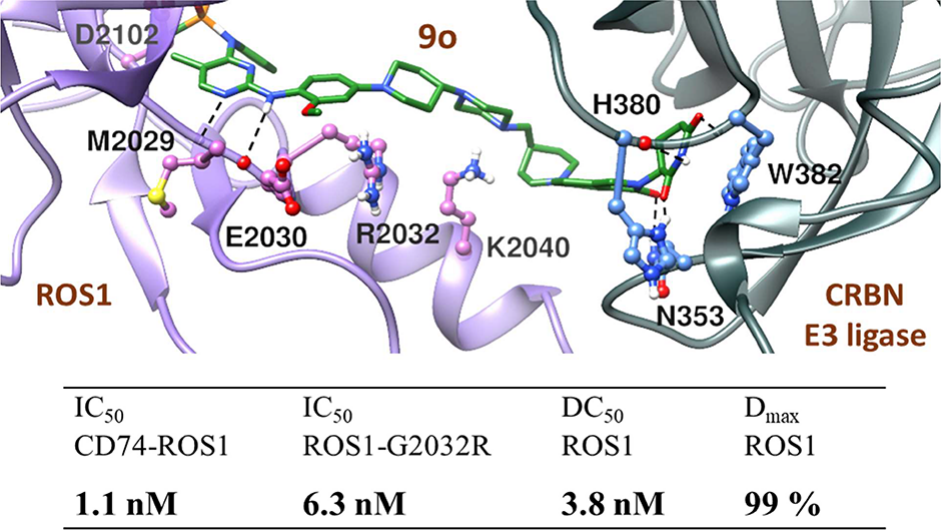

The development of therapeutic resistance in the majority of patients limits the long-term benefit of ROS1 inhibitor treatment. On-target mutations of the ROS1 kinase domain confer resistance to crizotinib and lorlatinib in more than one-third of acquired resistance cases with no current effective treatment option. As an alternative to stoichiometric inhibition, proteolytic degradation of ROS1 could provide an effective tool to combat resistance generated by these mutations. Our study has identified a potent, orally active ROS1 degrader with an excellent pharmacokinetics profile. The degrader can effectively inhibit ROS1-dependent cell proliferation and tumor growth by degrading the ROS1 kinase, thereby eliminating the active phospho-ROS1. More importantly, the degradation-based therapeutic modality can overcome on-target mutation resistance to tyrosine kinase inhibitors by efficient degradation of the mutated kinase to achieve greater potency than inhibition.

## Introduction

ROS1 is a receptor tyrosine kinase encoded by the ROS1 proto-oncogene.^1^ Chromosomal rearrangements involving ROS1 and a fusion partner generate constitutively active ROS1 fusion kinases that are oncogenic drivers in human cancers.^2^ ROS1 fusions are found in approximately 2% of late-stage nonsmall cell lung cancer (NSCLC) with CD74-ROS1 and SLC34A2-ROS1 as the most common rearrangements.^3^ The ROS1 kinase domain shares 70% identity with the anaplastic lymphoma kinase (ALK), and ROS1 gene rearrangements are mutually exclusive of ALK rearrangements. Consistent with their high homology, ROS1- and ALK-rearranged NSCLCs have been treated with shared tyrosine kinase inhibitors (TKIs). Crizotinib, an ALK/ROS1/cMET inhibitor, achieved 72% objective response rate and 19.2 months progression-free survival, and was the first TKI to receive FDA and EMA approval for the treatment of advanced ROS1 fusion–positive lung cancer.^4^ A second multikinase inhibitor, Entrectinib, with intracranial activity against ROS1, ALK, and TRK, was approved for the treatment of metastatic ROS1-positive nonsmall cell lung cancer (ROS1+ NSCLC) based on data from an integrated efficacy analysis of the STARTRK-1, STARTRK-2, and ALKA-372-001 clinical trials.^5,6^

Despite the initial robust benefit of ROS1 inhibitor treatment, therapeutic resistance eventually develops as a clinical challenge in the majority of patients, limiting duration of benefit to these therapies. On-target mutations of the ROS1 kinase domain have been identified in patient samples, including the most commonly occurring ROS1 mutation G2032R, accounting for approximately 33–41% as well as other common mutations that include D2033N (2.4–6%), L2026 M (1%), L1951R (1%), L2086F (3.6%), and S1986F/Y (2.4–6%).^7−13^ Some of these ROS1 mutations confer resistance to crizotinib and lorlatinib in more than one-third of acquired resistance cases with no current effective treatment option, but they are still valid targets of modulation by new inhibitors that can bind to the mutated ATP binding domain.

As an alternative to stoichiometric inhibition, proteolytic degradation of ROS1 could have dramatic consequences in ROS1 function. Removal of the ROS1 protein would eliminate ROS1 kinase activity as well as any protein interaction or scaffolding function of ROS1. Specific degradation of ROS1 could be accomplished by bifunctional small molecules to recruit ROS1 to a ubiquitin ligase and thus promote ubiquitylation and proteasomal degradation of ROS1. This unique therapeutic approach could more completely blockade ROS1 signaling than stoichiometric ROS1 inhibition, as short-term ternary complex formation would be sufficient to induce degradation, while stoichiometric inhibitors require long-term stable interactions. Furthermore, this degradation approach could effectively target the compound mutation forms of ROS1, overcoming resistance to current ROS1 inhibitors.

**Figure 1:**
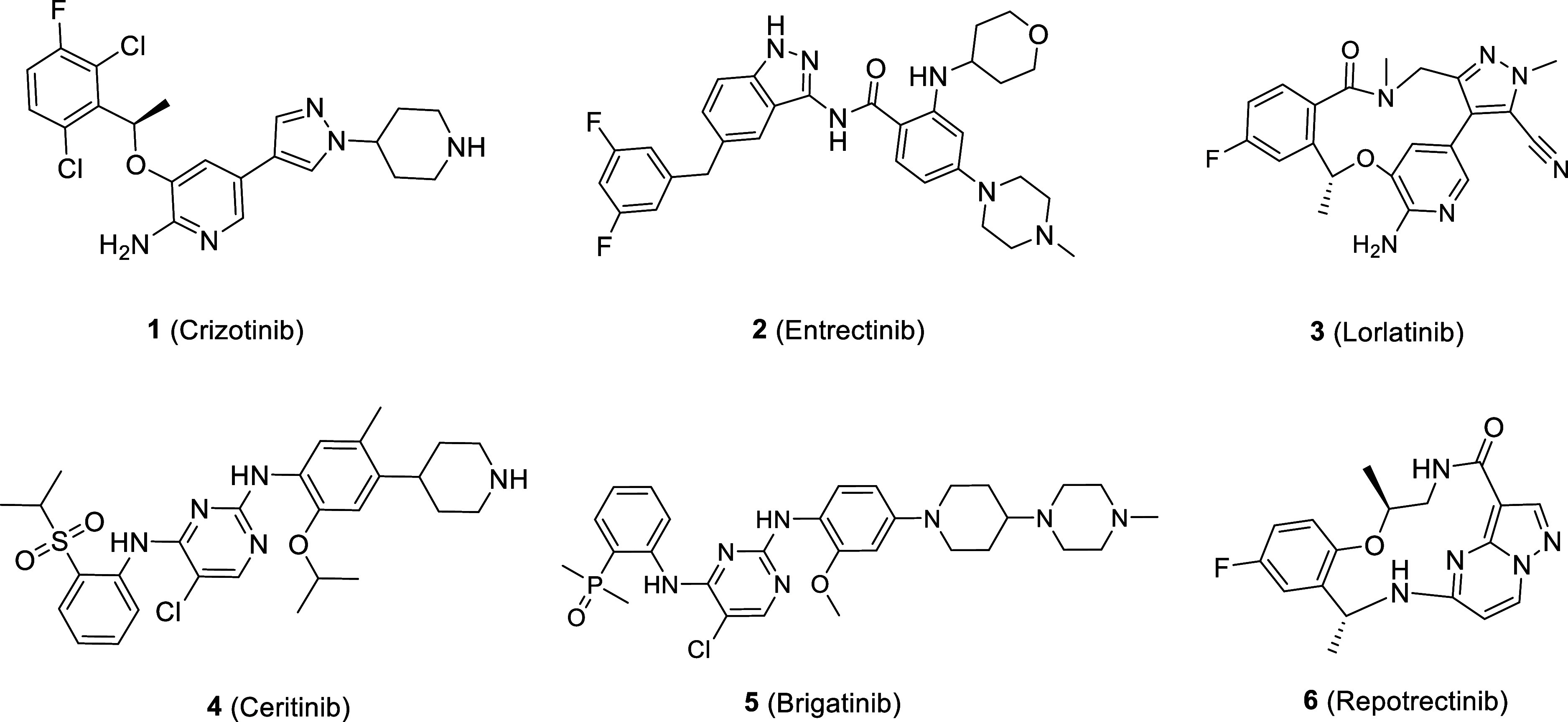
Structures of clinically validated ROS1 inhibitors.

We have designed and constructed a small library of ROS1 degrading molecules by conjugating a ROS1 binding moiety to a cereblon (CRBN) E3 ligase-binding ligand through diverse linker structures. Given multiple clinically validated ROS1 active inhibitors (Figure 1), we utilized three ROS1 recruiting elements as the warhead component, optimized linker length and composition for each warhead, and included various E3 ligands for avoidance of off-target degradation of CRBN neosubstrates. We have identified lead ROS1 degrading compounds that are potent, selective, orally active, and effective against all ROS1 resistance mutations that were assessed.

## Results

### Ba/F3 Cell Models with ROS1 Driver Gene and Secondary/Tertiary Mutations

To assess targeted degradation of ROS1 fusion proteins, we first established a mouse pro-B cell line Ba/F3 as a model expressing the CD74-ROS1 oncogene or CD74-ROS1 with clinically observed secondary/tertiary mutations. The Ba/F3 cell line is dependent on interleukin-3 for survival and proliferation. Upon transduction of oncogenic ROS1, Ba/F3 cells become independent of interleukin-3 but dependent on the transduced driver gene. The Ba/F3 models have been effectively employed in the discovery and preclinical development of next generation ROS1 inhibitors.^11,14,15^ We have tested ROS1 inhibitors against these Ba/F3 cells to validate their sensitivity and resistance to ROS1 inhibition. As shown in Table 1, the inhibition profiles of existing ROS1 inhibitors are largely consistent with reported data,^13^ confirming that these cell models are suitable for screening and evaluating potential ROS1 targeting degrader agents.

**Table 1:** TKI Growth Inhibition IC_50_ of Ba/F3 Cells with ROS1 Non-Mutant and Resistance Mutations^a^

	IC_50_ (nM)					
inhibitor	CD74-ROS1 non mutant	G2032R	L2026M	L2086F	D2033N	G2032R-F2075V
crizotinib	16.0 ± 3.5	>2000	77 ± 9	745 ± 88	187 ± 39	>2000
entrectinib	2.4 ± 0.4	980 ± 77	56 ± 7	506 ± 97	132 ± 15	>2000
ceritinib	14.5 ± 2.2	821 ± 148	43 ± 5	215 ± 33	135 ± 27	>2000
brigatinib	13.2 ± 2.7	265 ± 73	48 ± 4	172 ± 24	114 ± 17	327 ± 43
lorlatinib	0.3 ± 0.1	338 ± 55	1.0 ± 0.4	>2000	7.6 ± 1.3	233 ± 31
repotrectinib	0.7 ± 0.3	10.6 ± 1.9	10.3 ± 2.1	>2000	101 ± 32	10.7 ± 1.8

a IC_50_ represents the concentration needed to inhibit cell growth by 50%.

### Structural Analysis of ROS1 Inhibitors

**Figure 2:**
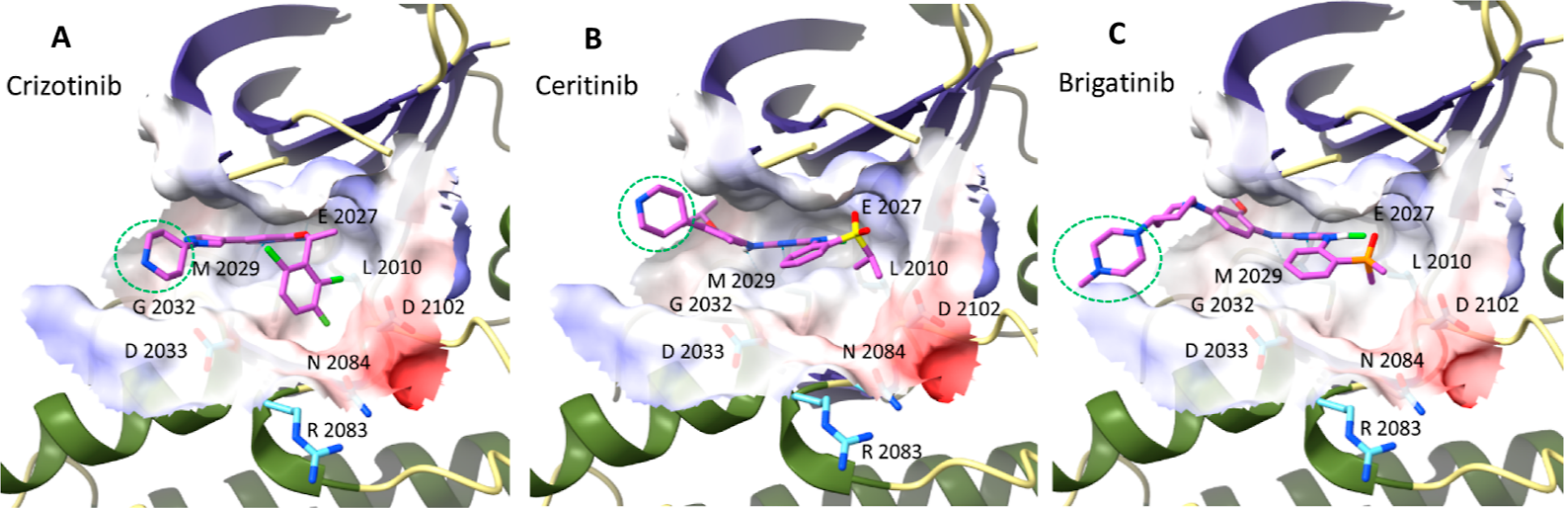
Binding modes of clinically validated ROS1 inhibitors with the wild-type ROS1 kinase domain. (A) Crystal structure of ROS1 complexed with crizotinib (PDB: 3ZBF), (B) docking pose of ceritinib in the binding site of ROS1 (PDB: 3ZBF), and (C) docking pose of brigatinib in the binding site of ROS1 (PDB: 3ZBF). The ligand molecules (purple) and several important residues are shown in stick. The binding pockets are shown in surface representation with electrostatic potential. The structural moiety protruding toward or out of the solvent front is marked in a dotted green circle.

To determine the linker sites on the ROS1 inhibitors utilized in this study to develop ROS1 degraders, detailed analyses of the crystal structure and/or in silico structural models were conducted. The binding modes of these inhibitors, (i.e., crizotinib, ceritinib, and brigatinib) in the active site of ROS1 are depicted in Figure 2. All three inhibitors bind similarly to the active site of ROS1 with their hinge binding cores (i.e., pyridinyl of crizotinib and the pyrimidine of ceritinib and brigatinib) anchored to the hinge region of the ROS1 kinase domain through hydrogen bonds with M2029 backbone. In addition, the amine group of pyridine-2-amine in crizotinib also forms a hydrogen bond with E2027 backbone. Similarly, the (2,6-dichloro-3-fluoro)phenyl moiety of crizotinib, the (propane-2yl sulphonyl)phenyl moiety of ceritinib, and the (dimethyl-phosphoryl)phenyl moiety of brigatinib were found to occupy the hydrophobic pocket formed by amino acid residues including C2085, L2086, and G2101. The piperidine ring of crizotinib and ceritinib and the *N*-methylpiperazine of brigatinib are largely protruded toward the solvent front region. Thus, we selected these protruding structural moieties for tethering a linker molecule as marked in Figure 2.

### ROS1 Degraders Based on Crizotinib as a Warhead

Crizotinib was the first ROS1 inhibitor approved as a front-line treatment for ROS1+ NSCLC. It has an IC_50_ of 9.8 nM against CD74-ROS1+ cells. Informed by the crystal structure of crizotinib in the ROS1 ATP binding domain where the piperidine ring is exposed to a solvent (Figure 2A), we decided on the cyclic nitrogen as the point of linker connection.

We designed and prepared the **7** series of molecules linking crizotinib as the ROS1-binding motif to thalidomide as the CRBN ligand (Table 2). When tested against CD74-ROS1+ cells, these crizotinib-based degraders showed modest activities of ROS1 degradation and cell growth inhibition (CGI). Linker connection at 5′ position of thalidomide appears to afford greater activities than 4′ position of thalidomide when comparing **7a** to **7e**, **7b** to **7f**, and **7c** to **7g**. An exception to this comparison is **7d** vs **7h** where linker connection at the 5′ position of thalidomide shows lower degradation potency than the 4′ position counterpart **7h**. Overall, optimal linker length is within the range explored, but no clear proportionality is present. Rather, steric factor appears to impact degrading activity more significantly as seen in the dramatic loss of activity in **7b**.

**Table 2:**
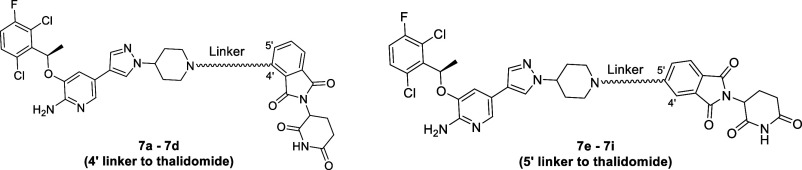

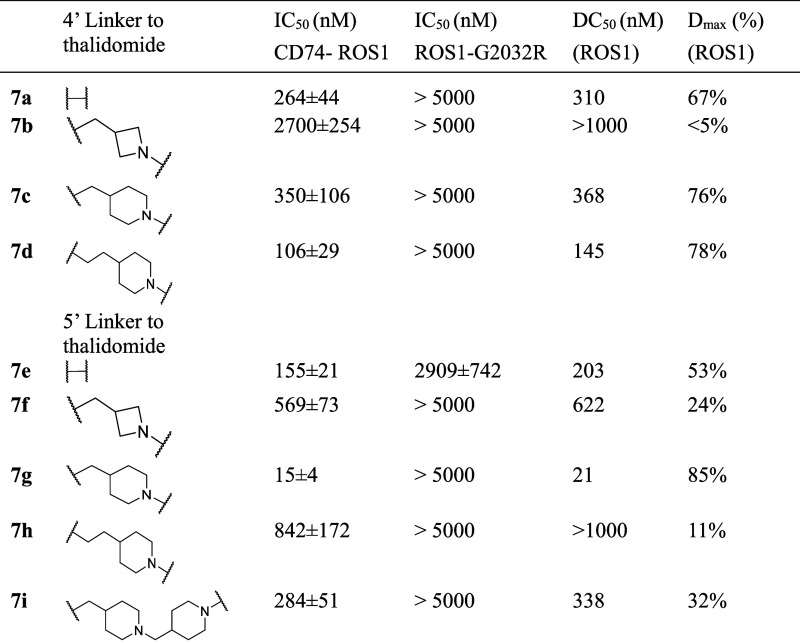
Antiproliferation and Degradation Activities of Crizotinib-Based Degraders **7a–7i** with Thalidomide as the E3 Binding Moiety^a^

a IC_50_ represents the concentration needed to inhibit cell growth by 50%. DC_50_ is the concentration needed to reduce ROS1 or *p*-ROS1 protein by 50%. *D*_max_ is the maximal degradation of ROS1 or *p*-ROS1 in Ba/F3 cells.

When tested against CD74-ROS1 cells with the resistance mutation G2032R, **7a–7h** showed no activity in inhibiting cell growth. This suggests that the binding affinity of crizotinib to the ROS kinase domain with this particular solvent front mutation (ROS1-G2032R) is insufficient to afford meaningful degradation activity to a degrader that requires sufficient engagement of the target protein.

### ROS1 Degraders Based on Ceritinib as the Warhead

We next turned to ceritinib, an effective first-line treatment for c-Ros oncogene 1 (ROS1)-rearranged nonsmall-cell lung cancer (NSCLC).^16,17^ Both crizotinib and ceritinib are recommended by the NCCN (National Comprehensive Cancer Network) guidelines as first-line treatment for patients with advanced ROS1+ NSCLC.^17^ Ceritinib inhibits ROS1 and has nanomolar-range half maximal inhibitory concentration (IC_50_) values in Ba/F3 cell lines engineered to express the oncogenic ROS1 rearrangement (IC_50_ = 180 nM) and in an NSCLC cell line HCC78 (IC_50_ = 50 nM).^16^

The in silico model of the ROS1-ceritinib complex (Figure 2B) shows that the apparent linker tethering site of ceritinib is ideally on the piperidine nitrogen. We first generated ceritinib degraders **8a**–**8g** that are tethered to 4′-thalidomide with varying linker lengths and compositions (Table 3). In nonmutant ROS1 cells, there is modest improvement in ROS1 degradation activity and CGI in Ba/F3 CD74-ROS1 cells with a narrow IC_50_ range of 100–600 nM. When tested against the Ba/F3 ROS1 cell line with a solvent front mutation G2032R, no significant activity was observed in ROS1-G2032R cells where neither CGI nor downregulation of ROS1 G2032R was effected by **8a**–**8g**.

**Table 3:**
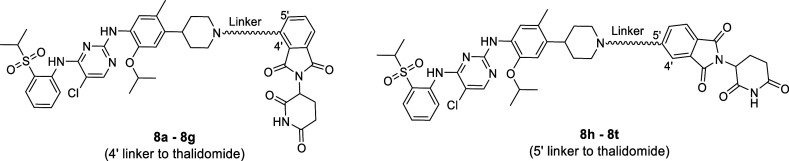

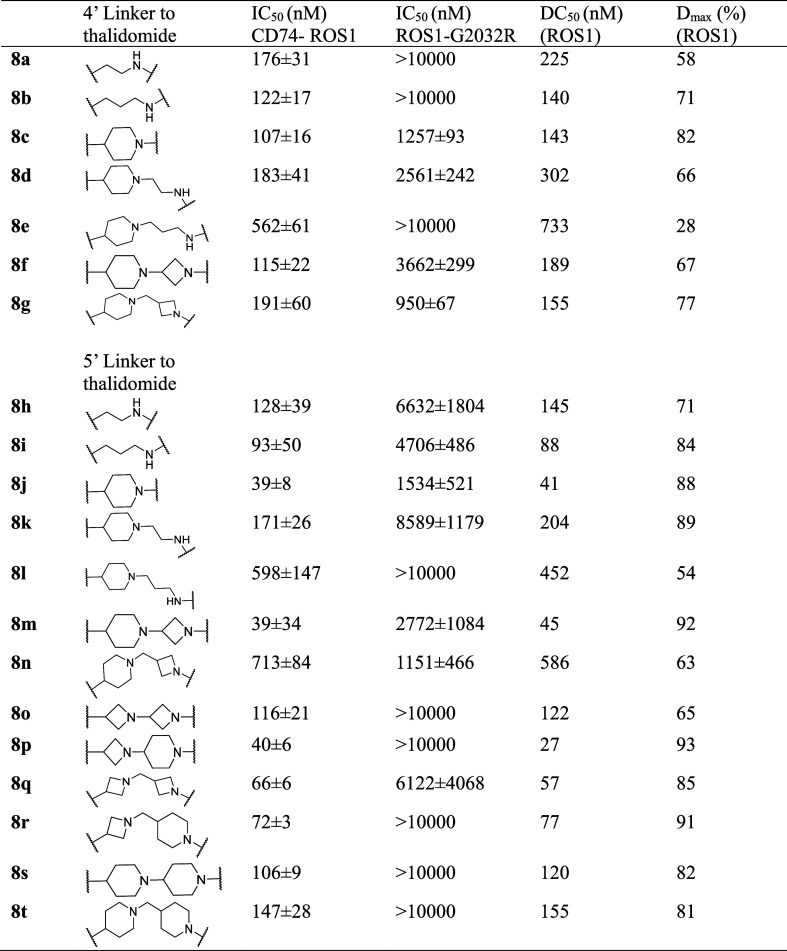
Antiproliferation and Degradation Activities of Ceritinib-Based Degraders **8a–8t** with Thalidomide as the E3 Binding Moiety

Connecting various linkers to 5′-thalidomide yielded **8h**–**8t** compounds (Table 3) that show overall improvement in activity against nonmutant ROS1 kinase as measured by degradation of ROS1 and inhibition of Ba/F3 cell growth. However, no activity was observed in ROS1-dependent Ba/F3 cells with resistant mutation (G2032R), likely due to diminished ceritinib binding affinity to ROS1 G2032R (Table 1).

### ROS1 Degraders Based on Brigatinib as the Warhead

Brigatinib is a potent ALK/ROS1 inhibitor with activity against a wide range of ROS1 kinase mutations. Brigatinib inhibits the phosphorylation of ROS1 and ERK in CD74–ROS1 and L2026 M mutant–transformed Ba/F3 cells, with less activity seen in the CD74–ROS1 G2032R-transformed Ba/F3 cells.^18,19^ Modeling of ROS1 in complex with brigatinib indicates the piperazine ring extends into the solvent front area and would be an ideal point of linker attachment (Figure 2C). We next generated the **9** series of brigatinib-based ROS1 degraders by conjugating the brigatinib core structure to CRBN binding variants through diverse linker compositions as shown in Table 4 (**9a**–**9k**) and Table 5 (**9l**–**9v**).

**Table 4:**
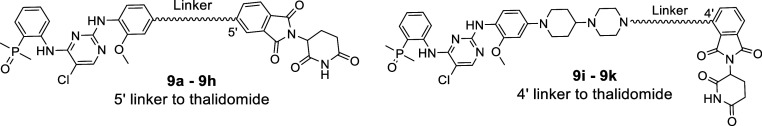

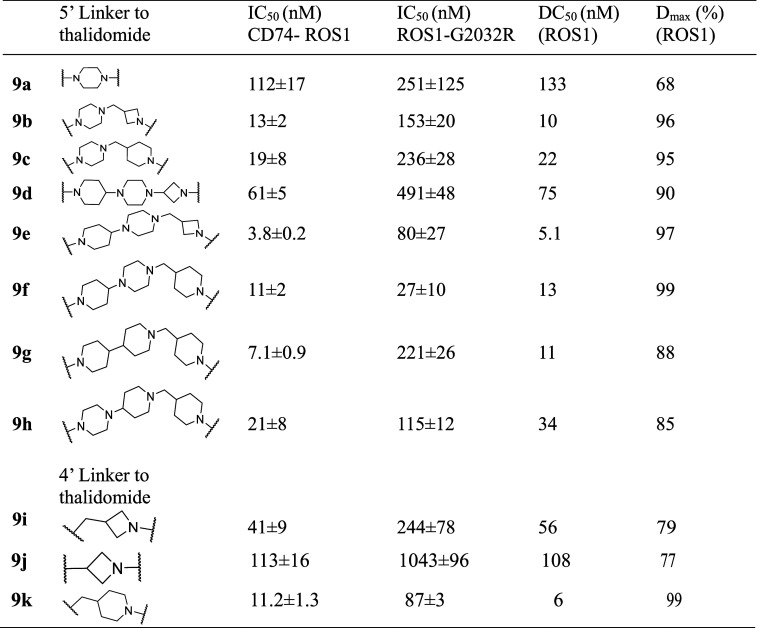
Antiproliferation and Degradation Activities of Brigatinib-Based Degraders **9a–9k** with Thalidomide as the E3 Binding Moiety

**Table 5:**
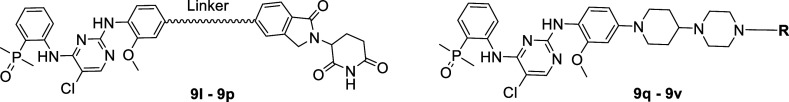

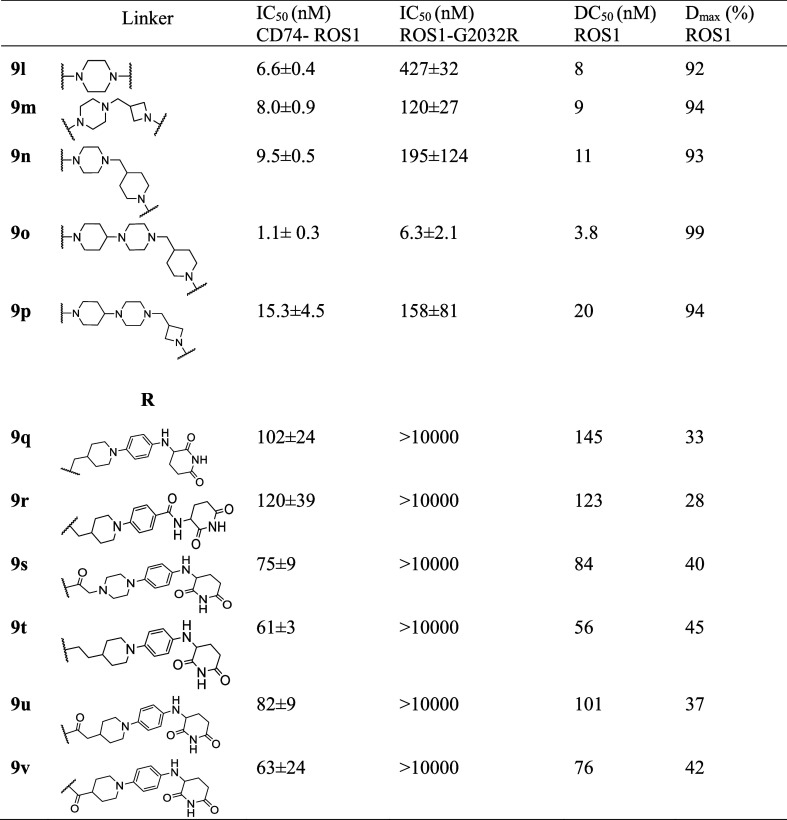
Antiproliferation and Degradation Activities of Brigatinib-Based Degraders **9l–9v** with Lenalidomide or Other Glutarimide Variants

In **9a**–**9h** where the linker is tethered to 5′-thalidomide, low nanomolar activities have been reached in several analogues (**9e**, **9f**, **9g**) against WT ROS1 cells. When the linker is tethered to 4′-thalidomide, the most active compound **9k** has the same linker and warhead composition as **9f** with comparable potency in CGI and ROS1 degradation. However, **9i** is ten times less active than the corresponding 5′ analogue **9e**; similarly, **9j** is also less potent than the corresponding 5′ analogue (**9d**). These observations suggest that 4′ attachment is generally inferior to 5′ connection in the brigatinib-based degrader construction.

In the resistant ROS1 G2032R mutant, these compounds have now gained moderate to potent activity in inhibiting cell growth. The most active compound, **9f**, achieved IC_50_ of 27 nM against the resistant mutant, followed by **9e** at 80 nM, a remarkable improvement over ceritinib- and crizotinib-based analogues.

We next tried to modify the E3 binding moiety in the most active analogues (**9a**, **9b**, **9c**, **9e**, and **9f**) to further improve the overall potency of the degraders. Switching from thalidomide to lenalidomide (deoxythalidomide) was found to enhance activity significantly in all cases but one where 5–20-fold increase in activity was achieved. For instance, from **9a** to **9l**, activity in ROS1 increased from 112 nM to 6.6 nM. More importantly, the lenalidomide analogue **9o** has gained low nanomolar activity in both nonmutant and solvent front mutant ROS1 driven cell growth (1.1 and 6.3 nM, respectively) compared to its thalidomide analogue **9f** (11 and 27 nM, respectively). In the exception case of switching from thalidomide in **9e** to lenalidomide in **9p**, instead of gain of activity, a 2-fold decrease was seen in the lenalidomide analogue **9p** (80 vs 158 nM in mutant ROS1).

However, changing the E3 ligand to other variants such as *N*-aryl glutarimide with or without accompanying linker modifications mostly resulted in the reduction or loss of activity, especially against mutant ROS1. In **9q**–**9v**, while moderate activities against nonmutant ROS1 were maintained, there was a complete loss of activity against the resistant ROS1 G2032R mutant.

The names, SMILES format, Molecular formulas, and biochemical/biological data of all the ROS1 degraders studied in this manuscript (series **7**, **8** and **9**) are listed in the Supporting Information (Table S2).

**Figure 3:**
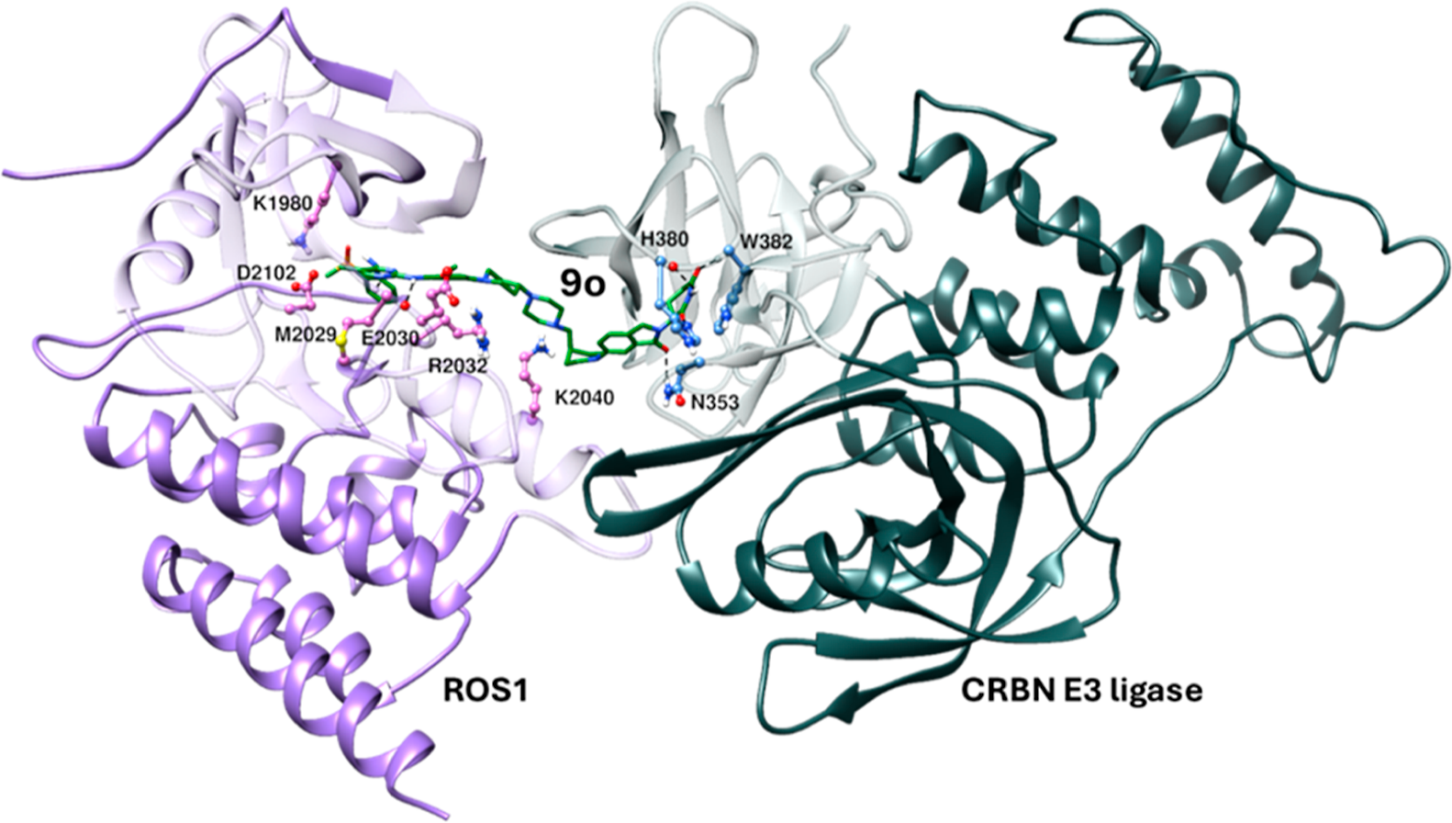
Putative in silico model of the most potent ROS1 degrader **9o**-based ternary complex involving ROS1 (PDB: 3ZBF) and CRBN E3 ligase (PDB: 4CI2): green–degrader **9o**, purple ribbon—ROS1, dark slate gray ribbon—CRBN E3 ligase. Important amino acid interactions with the degrader are shown in stick models.

A putative in silico model of the PROTAC mediated ternary structure consisting of the most active degrader **9o**, ROS1, and CRBN E3 ligase is depicted in Figure 3. Binding modes of the brigatinib part in **9o** to ROS1 and the lenalidomide part in **9o** to E3 ligase in the ternary complex are similar to when they are individually bound to their respective binding proteins; ROS1 and E3 ligase, respectively. These include the molecular interaction of the diaminopyrimidine group of brigatinib with the hinge residue M2029, and the DMPO moiety of brigatinib with K1980 and D2102 in ROS1, similarly the glutarimide group of lenalidomide with H380 and W382, and the isoindolinone group of lenalidomide with N353 in E3 ligase. In addition, in the ternary complex, the piperazine group of brigatinib also makes a hydrogen bond interaction with K2040. The ternary structure provided a detailed molecular interaction between the degrader **9o** and its two binding partners. Although we report no mutagenesis or X-ray crystallographic studies, binding of the degrader is more likely based on the ternary model obtained by the in silico studies. Conceptually, based on our molecular modeling studies, we should theoretically be able to optimize and design degraders that maintain high potency and efficacy against clinically relevant ROS1 mutants.

### Activity and Degradation of ROS1 and *p*-ROS1 in Ba/F3 Cells with Resistant Mutations

**Figure 4:**
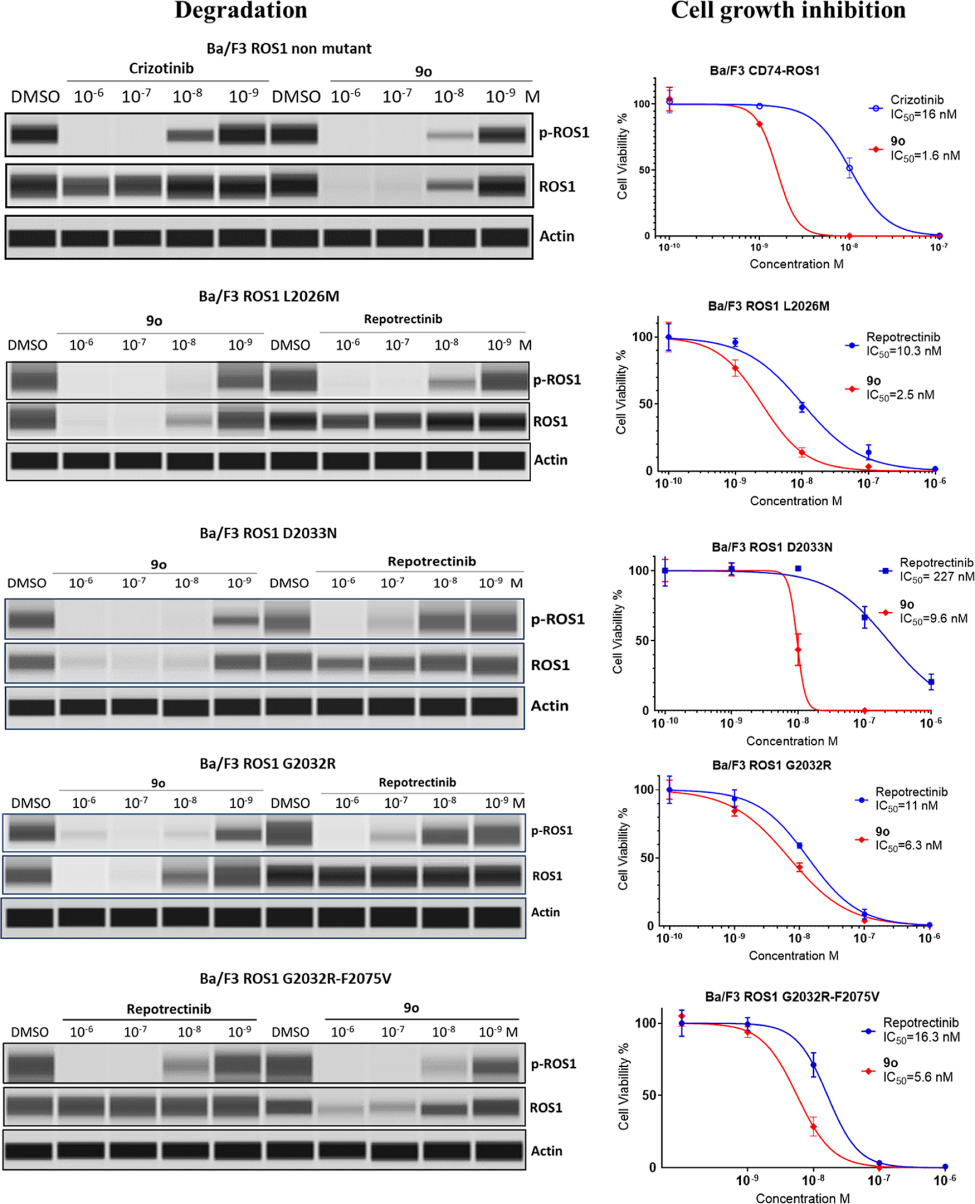
Degradation of ROS1 and *p*-ROS1 by **9o** and resulting CGI in Ba/F3 cells harboring ROS1 mutations.

Having identified the most potent ROS1 degrader (**9o**) in the three TKI based **7**, **8**, and **9** series, we next sought to evaluate its activity in clinically relevant ROS1 mutants that present resistance to current ROS inhibitors. These mutants are recapitulated in CD74-ROS1 and SLC34A2-ROS1 fusions, two prevailing ROS1 rearrangements in ROS1+ nonsmall cell lung cancers. As shown in Figure 4 and Table 6, **9o** degraded all mutant ROS1 and *p*-ROS1 dose-dependently with potency significantly greater than ROS1 inhibitors. In particular, in ROS1 mutations for which there are no available therapeutics, **9o** remained effective in degrading ROS1 and inhibiting cell growth. For instance, whereas **9o** maintained 1.9 to 66 nM potency against resistant mutations involving L2086F, S1986F/L2086F, S1986F/G2032R/L2086F, all inhibitors have lost activity against these mutant variants (Table 6).

**Table 6:** Activity of **9o** against an Expanded Set of Clinical ROS1 Mutations in Comparison with TKIs

	IC_50_ (nM)			
Ba/F3 cell line	brigatinib	lorlatinib	repotrectinib	**9o**
ROS1 (SLC34A2)	23.1 ± 1.9	0.4 ± 0.3	0.8 ± 0.3	1.1 ± 0.4
ROS1 (SLC34A2) D2033N	114.0 ± 25.3	7.6 ± 0.5	101 ± 27	9.7 ± 2.6
ROS1 (SLC34A2) L2026M	47.8 ± 10.2	1.1 ± 0.2	10.3 ± 1.9	2.5 ± 0.6
ROS1 (SLC34A2) V2098I	12.2 ± 3.1	1.5 ± 0.4	3.5 ± 0.6	4.6 ± 2.1
ROS1 (SLC34A2) G2032R	490.4 ± 38.4	244.0 ± 35.3	11.1 ± 2.0	6.3 ± 1.1
ROS1 (SLC34A2) G2032R-F2075 V	327.3 ± 44.8	340.9 ± 75.5	16.3 ± 2.9	5.6 ± 0.6
ROS1 (SLC34A2) F2075C-G1837E	8.1 ± 2.2	0.1 ± 0.1	0.4 ± 0.2	0.4 ± 0.3
ROS1 (SLC34A2) G2032R-D2113G	215.0 ± 50.3	142.1 ± 25.1	25.0 ± 6.8	2.9 ± 0.5
ROS1 (SLC34A2) G2032R-F2075C	869.4 ± 147.6	>2000	>2000	58.4 ± 11.4
ROS1 (CD74)	13.2 ± 2.9	0.3 ± 0.1	0.7 ± 0.2	1.6 ± 0.3
ROS1 (CD74) L2086F	172.2 ± 47.6	>2000	588 ± 76	6.9 ± 1.7
ROS1 (CD74) G2032R	512.9 ± 118.5	385.2 ± 76	23.5 ± 3.8	7.7 ± 2.0
ROS1 (CD74) G2032R-L2086F	693.5 ± 204.3	>2000	181.0 ± 22.5	66.2 ± 18.2
ROS1 (CD74) S1986F-L2086F	329.1 ± 85.5	>2000	>2000	1.9 ± 0.4
ROS1 (CD74) S1986F-G2032R	487.7 ± 96.8	503.4 ± 77.4	31.4 ± 7.3	49.5 ± 10.2
ROS1 (CD74) S1986F-G2032R-L2086F	>2000	>2000	>2000	37.8 ± 4.4

### Physicochemical Properties and Metabolic Stability Evaluation

An important physicochemical property for a PROTAC molecule is its lipophilicity that largely dictates its pharmaceutical performance such as absorption, permeability, and distribution. We measured Log *P* values of the best performing **9** compounds, as summarized in Table 7. These relatively large compounds show a narrow range of Log *P* between 2.5 and 3, indicating likely oral bioavailability, but not ideal oral drugs as Log *P* is not between 1.3 and 1.8 for a good oral drug.

**Table 7:** Physicochemical Properties and Metabolic Stability of Select ROS1 Degraders

		metabolic stability in rat microsomes		metabolic stability in human microsomes	
compound	log *P*	Cl_int_ (μL/min/mg)	*T*_1/2_ (min)	Cl_int_ (μL/min/mg)	*T*_1/2_ (min)
**9b**	2.84	13.8	100.5	119.5	29.0
**9c**	2.35	77.0	18.0	87.0	39.8
**9e**	2.50	12.8	108.3	57.0	60.8
**9f**	2.88	8.8	157.5	63.4	55.0
**9m**	3.07	9.6	144.4	94.5	36.9
**9o**	2.54	17.4	79.7	71.1	48.8

Metabolic stability of select **9** compounds were also determined in rat and human liver microsomes. As shown in Table 7, intrinsic clearance (Cl_int_) and half-life times of **9** compounds indicate moderate metabolic clearance, predicting some degree of oral bioavailability in rats and potentially humans.

We also analyzed in silico physicochemical and pharmaceutically significant properties of the six **9** compounds (see Supporting Information—Table S1), which determine their absorption, distribution, metabolism, excretion (ADME) properties. The predicted pharmacokinetic properties are within the acceptable range desired for human chemotherapeutic uses. However, the predicted apparent Caco2 cell permeability and apparent MDCK cell permeability are in the low range. This is expected because in general, the physicochemical property space of PROTAC falls beyond the rule of 5; and permeability drops off significantly with increasing molecular weight and total polar surface area.

### Pharmacokinetics and In Vivo Activity

We conducted pharmacokinetic profiling studies of the degraders that showed high potency against Ba/F3 cells with nonmutant oncogenic ROS1 and mutant ROS1. The best performing compounds were given to rats in a single oral dose to track plasma drug levels over the course of 24 h. As summarized in Table 8, at an oral dose of 10 mg/kg, **9b**, **9c**, and **9e** afforded 2345 to 4582 ng/mL *C*_max_ and area under the curve (AUC) of 25,437 to 41,622 h·ng/mL, demonstrating significant oral drug exposure in rats. The other three degraders, including two containing lenalidomide as E3-binding moiety (**9m**, **9o**, 5 mg/kg oral dose), gave moderate but sufficient oral drug exposure.

**Table 8:** Pharmacokinetic Profiles of Orally Bioavailable ROS1 Degraders

compound	dose (mg/kg, iv)	AUC (h·ng/mL)	dose (mg/kg, po)	*C*_max_ (ng/mL)	*T*_max_ (h)	*T*_1/2_ (h)	AUC (h·ng/mL)	% *F*
**9b**	2	50,451	10	4582	3	4.11	41,622	16.5
**9c**	2	29,380	10	2345	3	6.81	31,730	21.6
**9e**	1	20,850	10	3242	2	7.70	25,437	12.2
**9f**	1	3093	10	284	2	8.32	3031	9.8
**9m**	0.5	2151	5	145	1	8.88	1032	4.8
**9o**	0.5	1736	5	94	2	7.32	1267	7.3

**Figure 5:**
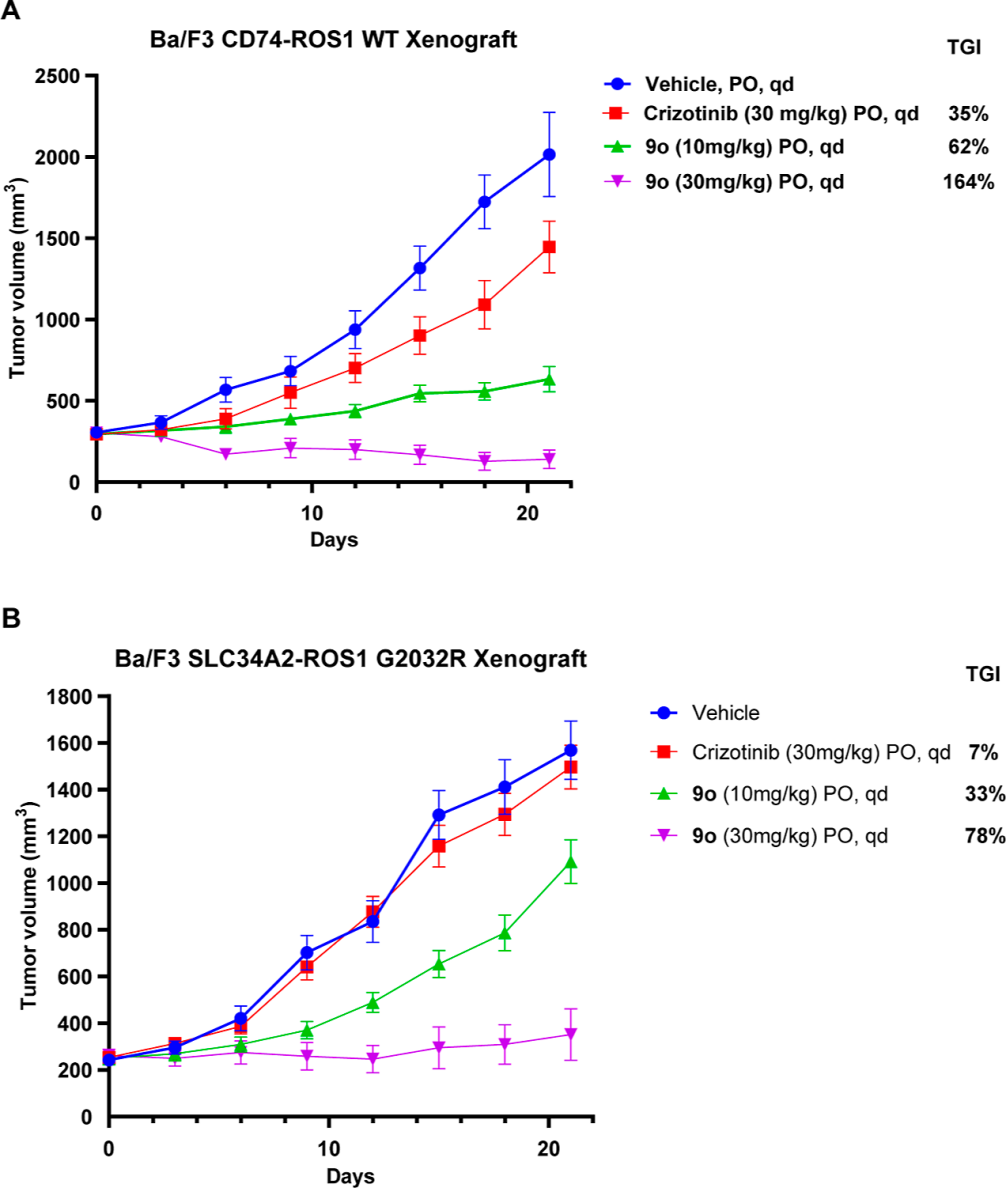
ROS degrader **9o** inhibits tumor growth in xenograft tumors harboring WT ROS1 as well as resistant ROS1 mutations. (A) Change in tumor volume of Ba/F3 CD74-ROS1 WT xenograft tumors. Mice were treated with vehicle (*n* = 5 mice), crizotinib 30 mg/kg QD (*n* = 5 mice), **9o** 10 mg/kg QD (*n* = 5 mice), or **9o** 30 mg/kg QD (*n* = 5 mice). Error bars indicate s.e.m. (B) Change in tumor volume of Ba/F3 CD74-ROS1-G2032R xenograft tumors. Mice were treated with crizotinib 30 mg/kg QD (*n* = 5 mice), **9o** 10 mg/kg QD (*n* = 5 mice), or **9o** 30 mg/kg QD (*n* = 5 mice). Error bars indicate s.e.m.

Having ascertained that the degrader molecules are orally bioavailable in rats, we next conducted in vivo efficacy studies by oral administration of **9o** in NOD/SCID mice bearing ROS1-dependent Ba/F3 xenografts. In the CD74-ROS1 xenograft (Figure 5A), as expected, crizotinib was modestly effective in inhibiting tumor growth, achieving 35% tumor growth inhibition (TGI). In contrast, once daily (q.d.) dosing with 10 or 30 mg/kg of **9o** resulted in TGI of 62% and 164%, respectively. In the TKI-resistant SLC34A2-ROS1 (G2032R) xenograft tumors (Figure 5B), **9o** at 10 and 30 mg/kg dosing achieved 33% and 78% TGI, respectively, while crizotinib treatment was ineffective (7% TGI).

### Kinase Binding and Degradation Selectivity

As the most active **9** compounds contain an intact brigatinib warhead, the kinase binding selectivity was profiled for **9o** against ROS1 kinase variants and a panel of kinases that show significant inhibition by brigatinib.^20^ Consistent with brigatinib binding selectivity, **9o** showed similar inhibition profile in some 20 kinases with IC_50_ < 100 nM (Table 9). However, the degradation selectivity of **9o** often contrasts with its kinase binding selectivity as determined in cells treated with **9o** where the kinase level was measurable by Western blot analysis. While **9o** showed potent degradation of ROS1, ALK, FAK, FER, consistent with its low nanomolar kinase inhibition, most of the kinases were not degraded with potency corresponding to their binding activity. For example, FLT3, CHEK2, and FES, all with kinase binding at <10 nM, were only moderately or minimally degraded, if at all (Table 9). Importantly, it is observed that **9o**′s reduced binding affinity to ROS1 mutants (IC_50_ = 161 nM for G2032R, IC_50_ = 218 nM for L2086F) also contrasts with its more potent degradation of the mutant ROS1 (DC_50_ = 13.2 nM for G2032R, DC_50_ = 5.4 nM for L2086F), providing evidence that with optimized ternary complex formation, moderate warhead binding may be sufficient to achieving high potency degradation.

**Table 9:** Inhibition and Degradation Activity of **9o** against Select Kinases^a^

kinase	kinase assay IC_50_, nM	degradation assay DC_50_, nM
ROS1	0.8	3.8
ROS1 (G2101A)	10.9	ND
ROS1 (G2032R)	161	13.2
ROS1 (G2101C)	83	ND
ROS1 (L2086F)	218	5.4
ALK	0.9	1.7
FAK/PTK2	2.4	1.5
FER	3.1	17
FLT3	3.5	>1000
CHEK2	3.6	187
FES	7.5	320
ERBB4/HER4	27	>1000
PTK2B	34	ND
CLK1	35	>1000
CAMK2D	39	264
CHEK1	40	ND
ERBB2	42	474
INSRR	45	ND
NUAK1	47	ND
CAMK2G	48	ND
LTK	54	>1000
EGFR	69	255
BRK (PTK6)	76	ND
RET	82	58
C-MET	95	>1000

a Data represent average of duplicate measurements. ND—not determined.

**Figure 6:**
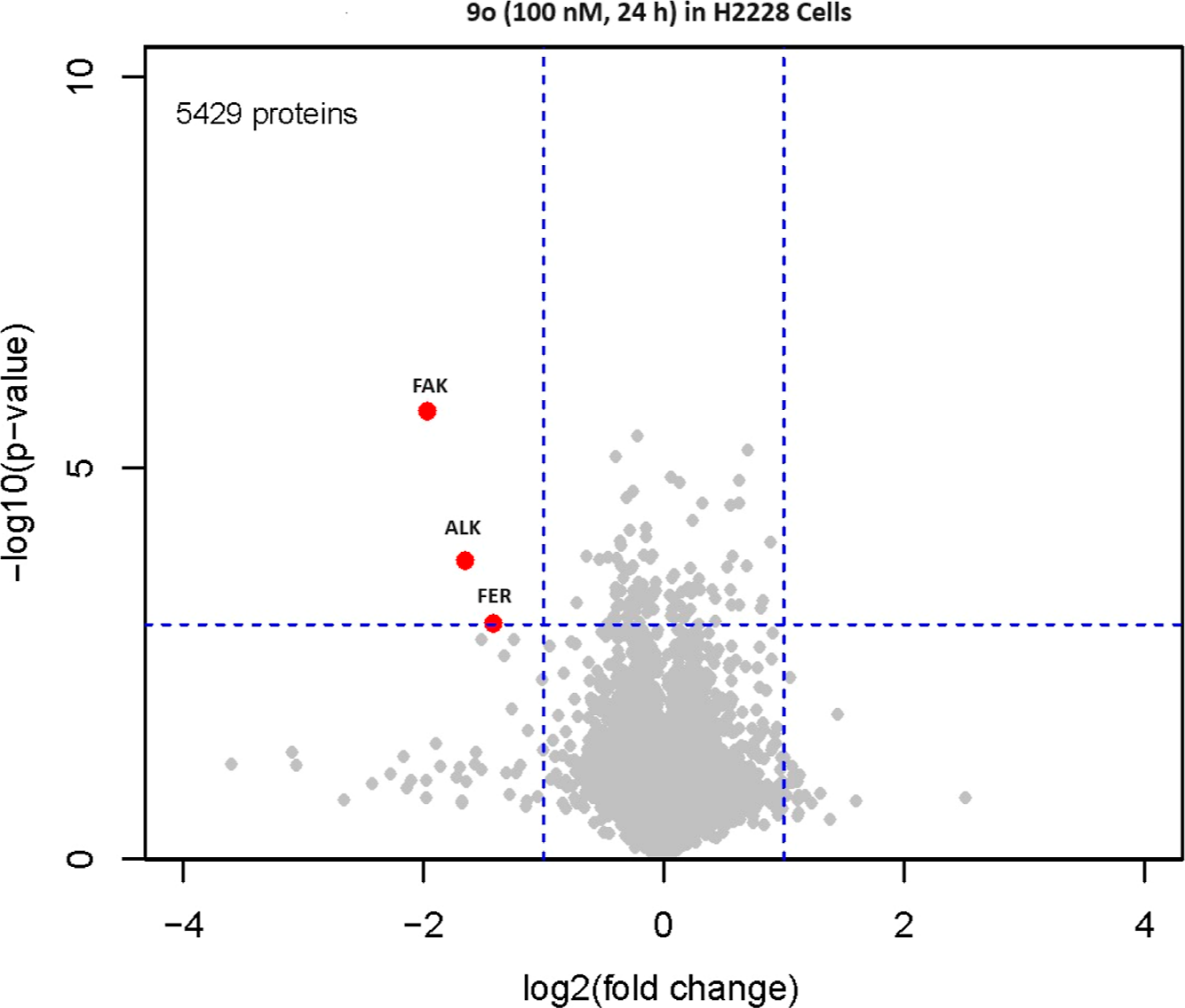
Whole cell proteomic analysis of H2228 cells treated with 100 nM **9o** for 24 h.

To profile the degradation selectivity of **9o**, we next conducted a proteomic study of a nonsmall cell lung cancer cell line (H2228) after treatment with **9o** (degrader) or brigatinib (inhibitor). To minimize the effect of signaling blockade on proteomic changes, we decided that a more accurate quantitative determination of proteomic alterations would be to compare degrader-treated to inhibitor-treated. As shown in Figure 6, only three kinases were significantly degraded after cells were treated with 100 nM **9o** for 24 h, consistent with degradation assay of **9o** in various cell systems (Table 9).

## Chemistry

**Scheme 1:**
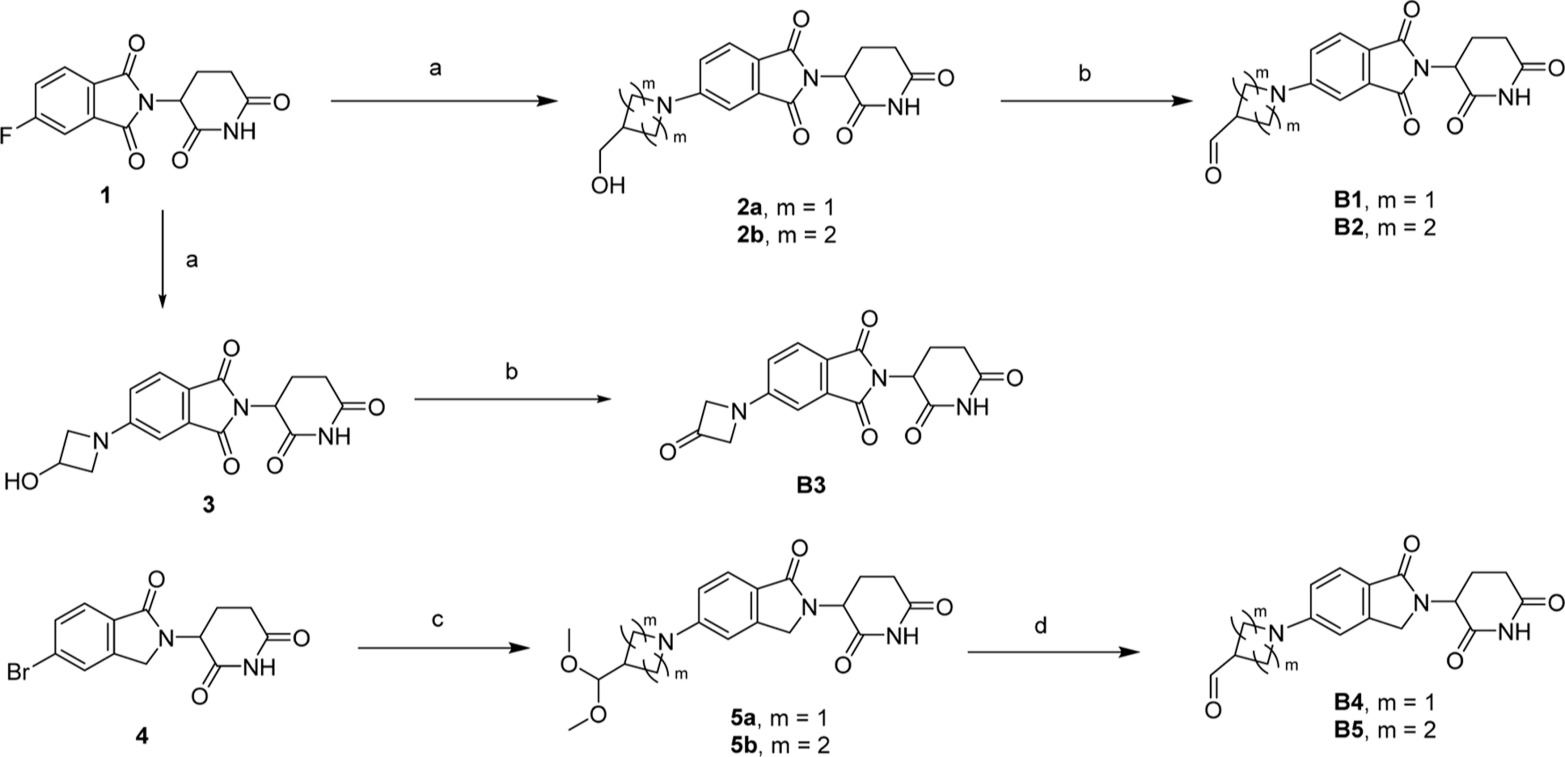
Synthesis of CRBN-Derived Building Blocks **B1–B5** Reagents and conditions: (a) amine, DIPEA, NMP, 120 °C, 1 h; (b) Dess–Martin’s reagent, DCM, rt, 1 h; (c) 3-(dimethoxymethyl)azetidine or 4-(dimethoxymethyl)piperidine, Pd-PEPPSI-IheptCl, Cs_2_CO_3_, DMSO, 80 °C, 16 h; (d) TFA, DCM, rt, 2 h or 2 M THF–H_2_O, rt.

The synthesis of the building block aldehydes/ketones is summarized in Scheme 1. Nucleophilic substitution of 2-(2,6-dioxopiperidin-3-yl)-5-fluoroisoindoline-1,3-dione (**1**) with the corresponding cycloalkylamines provides the alcohols (**2** and **3**), which were oxidized to be desired thalidomide-derived aldehydes/ketones (**B1–B3**) by Dess–Martin’s reagent. Pd-PEPPSI-IheptCl-catalyzed Buchwald–Hartwig C–N crossing coupling reaction of **4** provides acetals (**5**).^21^ TFA/DCM or 2 M HCl in THF-mediated deprotection of **5** gave the desired aldehydes (**B4** and **B5**), which were used directly as stating material for next reaction without further purification.

**Scheme 2:**
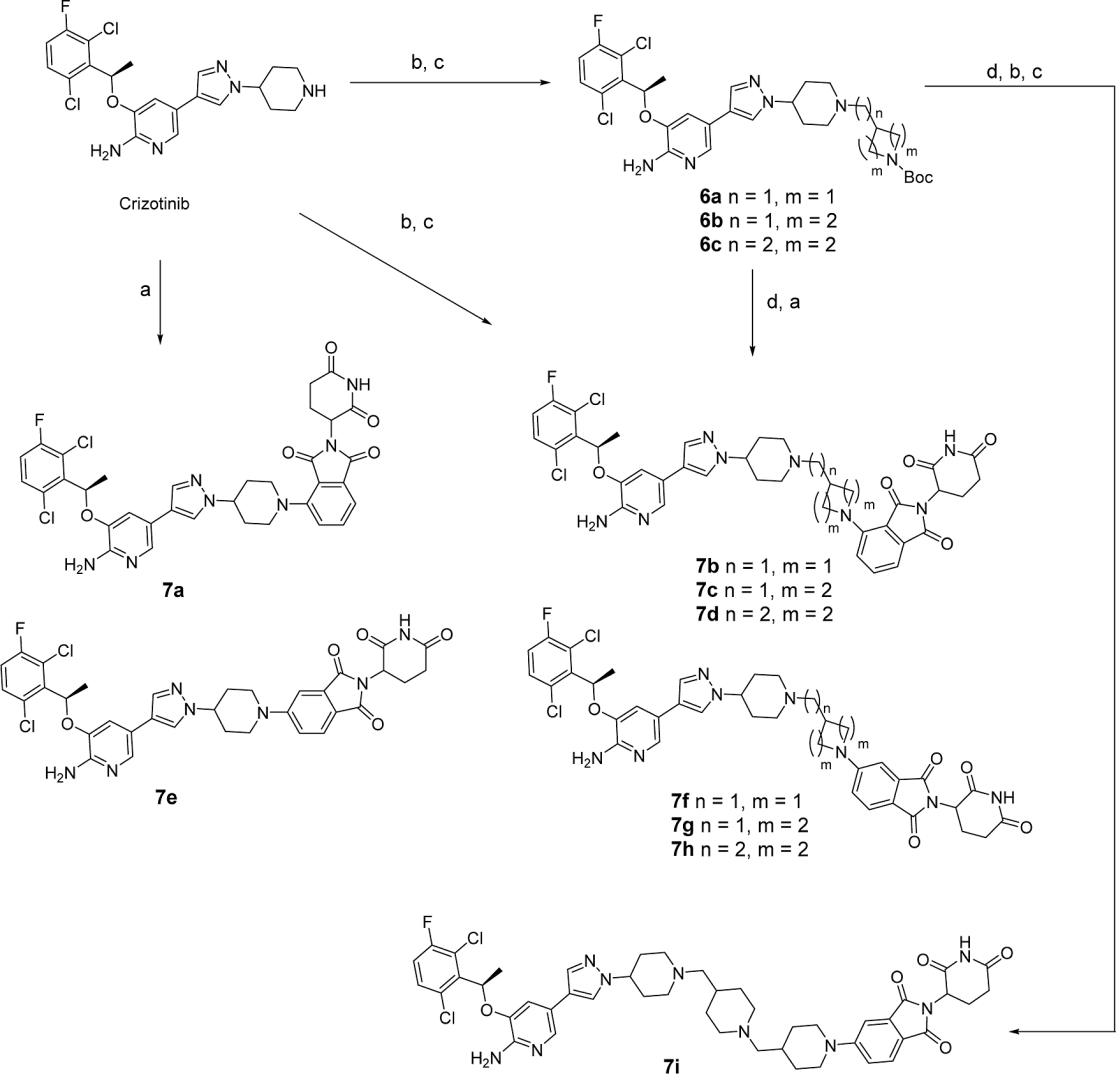
Synthesis of Compounds **7a–7i** in Table 2 Reagents and conditions: (a) **1** or **2**, DIPEA, NMP, MW, 100 °C, 1 h. (b) Et_3_N, DCM, aldehydes, rt, 1 h. (c) Na(OAc)_3_BH, rt, 2–3 h. (d) HCl, dioxane, rt, 1 h.

As shown in Scheme 2, the direct microwave-mediated condensation of crizotinib with 2-(2,6-dioxopiperidin-3-yl)-5-fluoroisoindoline-1,3-dione (**1**) or 2-(2,6-dioxopiperidin-3-yl)-4-fluoroisoindoline-1,3-dione (**2**) in NMP afforded **7a** and **7e**. The standard reductive amination (NaBH_3_CN as reductive agent) of amine-containing crizotinib with aldehyde **B2** gave **7g** at room temperature in moderate yield. Compounds **7b**–**7d**, **7f**, and **7h** were obtained from the microwave-mediated condensation of **1** or **2** with the deprotected intermediate amines from **6a**–**6c**, which were prepared by the standard reductive amination of crizotinib and aldehydes. Further reductive amination of the deprotected intermediate amines from **6b** with **B2** resulted in the formation of **7i**.

**Scheme 3:**
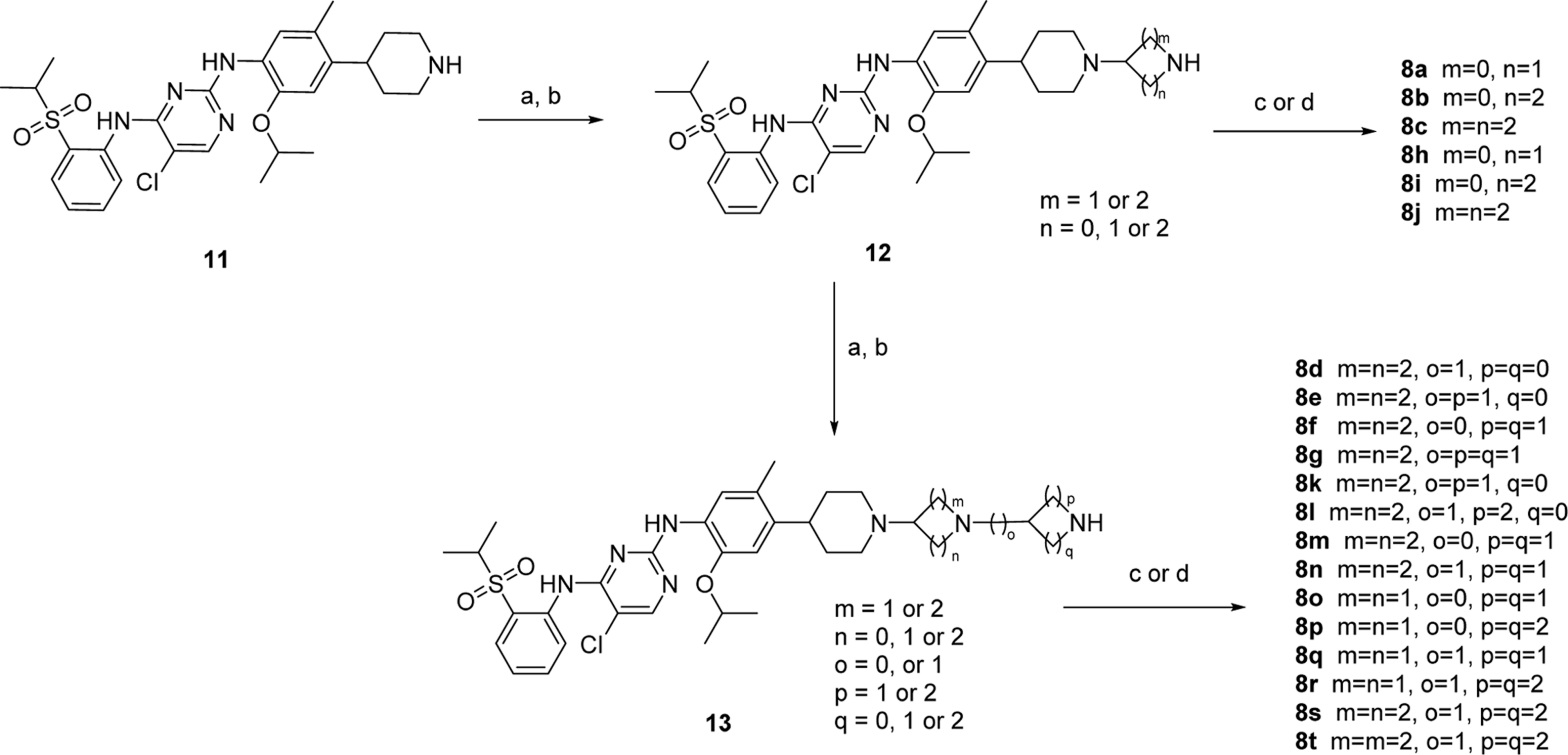
Synthesis of Compounds **8a–8t** in Table 3 Reagents and conditions: (a) ketones, STAB, NEt_3_, DCM, rt; (b) TFA, DCM or 4 M HCl in dioxane; (c) 2-(2,6-dioxopiperidin-3-yl)-5-fluoro isoindoline-1,3-dione, DIEA, NMP, MW, 120 °C; (d) 2-(2,6-dioxopiperidin-3-yl)-4-fluoroisoindoline-1,3-dione, DIEA, NMP, MW, 120 °C.

Ceritinib-based degraders were synthesized as shown in Scheme 3. Different linear or cyclic aldehydes/ketones reacted with ceritinib (**11**) via standard reductive amination to provide **12**. To vary the length of linkers, shorter linker degraders **8a–8c** and **8h–8j** were synthesized via microwave-mediated aromatic nucleoaddition reactions with 2-(2,6-dioxopiperidin-3-yl)-5-fluoroisoindoline-1,3-dione (**1**) or 2-(2,6-dioxopiperidin-3-yl)-4-fluoroisoindoline-1,3-dione (**2**). While amines (**12**) were first extended the linker by reductive amination with and Boc deprotection, then aromatic nucleoaddition to give different length linkers in degraders **8d–8g** and **8k–8t**.

**Scheme 4:**
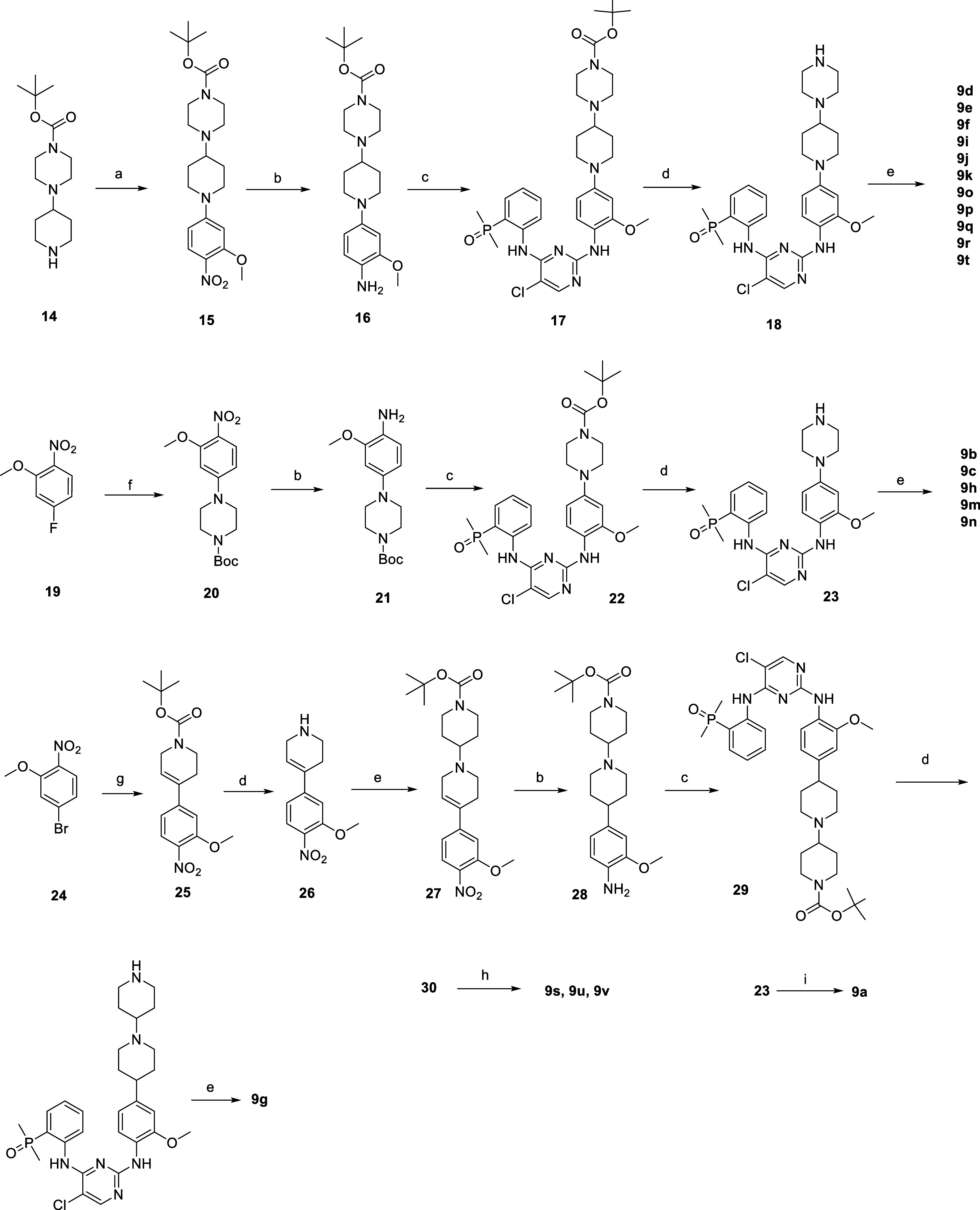
Synthesis of Compounds **9a–9v** in Tables 4 and 5 Reagents and conditions: (a) K_2_CO_3_, MeCN, reflux; (b) Pd/C, MeOH, H_2_; (c) (2-((2,5-dichloropyrimidin-4-yl)amino)phenyl)dimethylphosphine oxide, Pd_2_(dba)_3_, Xantphos, Cs_2_CO_3_, 1,4-dioxane, Ar, 100 °C; (d) 2 M HCl in MeOH; (e) ketone, STAB, NEt_3_, DCM, rt; (f) *tert*-butyl piperazine-1-carboxylate, K_2_CO_3_, DMF, 80 °C, 2 h; (g) *tert*-butyl 4-(4,4,5,5-tetramethyl-1,3,2-dioxaborolan-2-yl)-3,6-dihydropyridine-1(2*H*)-carboxylate, Pd(dppf)Cl_2_, 1,4-dioxane-H_2_O, Ar, 100 °C; (h) corresponding acids, HATU, DIEA, DMF, rt; (i) 2-(2,6-dioxopiperidin-3-yl)-5-fluoroisoindoline-1,3-dione, DIEA, NMP, DIEA, MW, 120 °C.

Brigatinib-based degraders were synthesized as shown in Scheme 4. Amine **14** reacted with 4-fluoro-2-methoxy-1-nitrobenzene to provide the nitro compound **15**, which was reduced by palladium on charcoal under hydrogen atmosphere to afford aromatic amine **16**. Buchwald–Hartwig C–N cross coupling of **16** introduced the phosphine oxide head with moderate yield to give **17**. After, Boc deprotection of **17** followed by standard reductive amination of **18** with corresponding aldehydes/ketones resulted in the formation of **9d**–**9f**, **9i**–**9k**, and **9o**–**9t**. The piperidine of above compounds are positioned to the solvent front, which could also be considered as part of linker in the overall structure. Replacing piperidine with piperazine by similar methods led to **9a**–**9c** and **9m**–**9n**.

## Discussion and Conclusions

To achieve effective degradation of a target protein, a PROTAC molecule must be able to sufficiently bind both target protein and E3 ligase, thereby forming a stable ternary complex to allow ubiquitination of the target protein. In the initial attempt of utilizing crizotinib as the target binding motif, moderate activity of degradation of CD74-ROS1 was obtained in several crizotinib-conjugated derivatives (**7d**, **7g**). However, none of the crizotinib-based degraders showed meaningful activity against ROS1 harboring G2032R mutation. The sharp contrast suggests that the much-reduced binding of crizotinib to ROS1 G2032R (Table 1, IC_50_ > 2000 nM) is insufficient for the degraders to engage the mutant target protein.

Using ceritinib as a ROS1 warhead, the ensuring PROTACs yielded some improvement in ROS1 degradation activity, with compounds **8h**–**8t** showing moderate to high activity in CGI and ROS1 degradation. However, no significant activity was obtained in ceritinib-based degraders against ROS1 G2032R, likely due to the low binding affinity of the warhead against mutant ROS1 (Table 1, IC_50_ = 821 nM).

While brigatinib is comparable to crizotinib and ceritinib in its activity against CD74-ROS1, degraders constructed from the brigatinib warhead showed greater degradation potency in Ba/F3 cells expressing CD74-ROS1. Several brigatinib-based molecules (**9e**, **9f**, **9g**, **9l**, **9m**, **9n**, **9o**) reached single digit nanomolar potency in CGI and degradation of ROS1 fusion protein. The enhanced potency is likely a result of the overall optimal configurations that allow more efficient ternary complex formation and subsequent ubiquitination of the target protein.

More importantly, as brigatinib retained a significantly higher activity in mutant ROS1 (e.g., G2032R and L2086F) than crizotinib and ceritinib, the series **9** degraders are now conferred with degradation activity against ROS1 G2032R. Upon further optimization with linker adjustment and E3 ligand switching, **9o** emerged as the most potent degrader against CD74-ROS1 and the G2032R mutant. Furthermore, the potency of **9o** extends to all other resistant ROS1 mutants that are clinically relevant and tested in this study (Table 6). In particular, **9o** is effective against mutants that are resistant to repotrectinib, a next generation ROS1 inhibitor recently approved by FDA, including L2086F and G2032R-F2075C. The broad activity spectrum of **9o** demonstrates that degraders can not only overcome mutational resistance to currently approved ROS1 inhibitors but also address the vulnerability of next generation inhibitors that are not effective against certain on-target mutations such as L2098F and related combinations.

As degrader molecules are in the space of beyond the rule of 5,^22−24^ oral bioavailability is a critical factor in our design of ROS1 degraders if they are to be used as small molecule therapeutics. We have used CRBN ligands and linker structures that satisfy spatial requirements for ternary complex formation and contribute to solubility and permeability of the degrader molecule as a whole. The pharmacokinetic profiles of the most active ROS1 degraders (**9b**, **9c**, **9e**, **9f**, **9m**, and **9o**) in rats demonstrate moderate to excellent oral drug exposure at levels well above their respective therapeutically effective IC_50_ values. With good oral bioavailability, in vivo efficacy studies confirm that oral administration of the most effective degrader could achieve greater therapeutic outcome than a ROS1 inhibitor, especially in the TKI-resistant ROS1 mutant xenograft tumor.

Our study has identified a potent, orally active ROS1 degrader with excellent pharmacokinetics and mutant profile. The degrader can effectively inhibit ROS1-dependent cell proliferation and tumor growth by degrading the ROS1 kinase, thereby eliminating the active phospho-ROS1. More importantly, the degradation-based therapeutic modality can overcome on-target mutation resistance to TKIs by efficient degradation of the mutated kinase where moderate binding by the degrader warhead appears to be sufficient to achieve high potency.

## Experimental Section

### General Synthetic Methods

Unless otherwise noted, all commercial materials were used as received. NMR spectra were recorded on a Bruker Ascend 400 MHz spectrometer and calibrated using residual solvent peaks as internal reference. In reported spectral data, the format (δ) chemical shift (multiplicity, *J* values in Hz, integration) was used with the following abbreviations: s = singlet, d = doublet, t = triplet, q = quartet, hept = heptet, dd = doublet of doublets, and m = multiplet. Low resolution mass spectrometric (MS) analysis was carried out with a Shimadzu LCMS-2020. High-resolution mass experiments were operated on a Q-Exactive mass spectrometer (Thermo Scientific Instrument) with electrospray ionization source. Flash column chromatography was performed by Teledyne CombiFlash RF+ using the Agela Rf silica gel flash column. The final compounds were all purified by a C18 reverse phase preparative HPLC column (HYPERSIL Prep HS C18 5 μm, 20 × 150 mm) with solvent A (0.5% formic acid in H2O) and solvent B (0.1% formic acid in MeCN) as eluents at 10 mL/min flow rate. The purity of all the final compounds was measured and confirmed to be >95% by UPLC–MS analysis (10–100% MeCN in H2O containing 0.1% formic acid in 5 min, 1.0 mL/min flow rate) with a C18 column (ACQUITY UPLC BEH C18 1.7 μm, 2.1 × 50 mm).

#### General Procedure A. Reductive Amination

Amine (1 equiv) was dissolved in dry DCM, followed by adding 2 equiv of corresponding ketone or aldehyde and 5 equiv of triethylamine at room temperature. After stirring for 10 min, 2 equiv of STAB was added portionwise. After completion, the mixture was concentrated and subjected to silica gel to provide the intermediate.

#### General Procedure B. Removing Boc Protection

Two M HCl in MeOH or TFA/DCM was added to the above obtained intermediate at room temperature. After completion, the solvent was removed in vacuo and used in the next step without further purification.

#### General Procedure C. C–N Coupling by Buchwald–Hartwig Reaction with (2-((2,5-Dichloropyrimidin-4-yl)amino)phenyl)dimethylphosphine Oxide

A mixture of 1 equiv of amine and 1 equiv of (2-((2,5-dichloropyrimidin-4-yl)amino)phenyl)dimethylphosphine oxide, 0.05 equiv of Pd_2_(dba)_3_, 0.1 equiv Xantphos, and 2 equiv of Cs_2_CO_3_ was stirred and heated to 100 °C in an oil bath under Argon for 16 h. After completion, the mixture was filtered through Celite. The filtrate was evaporated in vacuo and the residue was subjected onto silica gel column chromatography to provide the desired intermediate.

#### General Procedure D. C–N Coupling with SN_Ar_ Reaction

A mixture of 1 equiv of amine and 1 equiv of 2-(2,6-dioxopiperidin-3-yl)-5-fluoroisoindoline-1,3-dione or 2-(2,6-dioxopiperidin-3-yl)-4-fluoroisoindoline-1,3-dione, 5 equiv of DIEA in NMP was heated to 120 °C in a microwave reactor for 1 h. After completion, the reaction mixture was subjected to silica gel column chromatography to provide the desired compound.

### Synthesis of Intermediates **B1**

To a solution of 2-(2,6-dioxo-3-piperidyl)-5-fluoro-isoindoline-1,3-dione (**31**) (2.0 g, 7.2 mmol) and azetidin-3-ylmethanol (0.95 g, 7.7 mmol) in DMA (5.0 mL) was added DIPEA (3.7 g, 0.29 mmol, 5.0 mL) at room temperature. Then, the solution was stirred at 120 °C for 3 h. Upon the reaction completion, the above reaction solution was cooled to room temperature, and the filtrate was concentrated under reduced pressure. The residue was purified by column chromatography (SiO_2_, petroleum ether: ethyl acetate, 3 to 97%) to give 2-(2,6-dioxo-3-piperidyl)-5-[3-(hydroxymethyl)azetidin-1-yl]isoindoline-1,3-dione (**32a**) (1.9 g, 67% yield) as a yellow solid. LCMS: 344 [M + H]^+^, ESI+; 1H NMR: (400 MHz, DMSO-*d*_6_): δ 11.06 (s, 1H), 7.63 (d, *J* = 8.4 Hz, 1H), 6.76 (d, *J* = 2 Hz, 1H), 6.62 (d, *J* = 8.4 Hz, 1H), 5.07–5.03 (m, 1H), 4.83 (t, *J* = 5.2 Hz, 1H), 4.07–4.00 (m, 3H), 3.78–3.75 (m, 2H), 3.60 (t, *J* = 5.6 Hz, 2H), 2.91–2.83 (m, 2H), 2.02–2.00 (m, 1H), 1.17 (t, *J* = 7.2 Hz, 1H).

To solution of the above-obtained (**32a**) (1.9 g, 5.5 mmol) in DCM (20 mL) was added DMP (4.7 g, 11.1 mmol). The resulting mixture was stirred at room temperature for 1 h. Upon the reaction completion, the mixture was quenched with 10% Na_2_SO_3_ aqueous solution and extracted with DCM (60 mL × 3). The filtrate was concentrated in vacuum, and the desired product was obtained in an aqueous layer. Then, the aqueous layer was lyophilized to give a yellow solid. The crude product was triturated with EA (10 mL) at 20 °C for 10 min and filtered to provide 1-[2-(2,6-dioxo-3-piperidyl)-1,3-dioxo-isoind-olin-5-yl]azetidine-3- carbaldehyde (**B1**) (2.5 g, 86% yield) as a yellow solid. 1H NMR: (400 MHz, DMSO-*d*_6_): δ 11.06 (s, 1H), 7.62 (d, *J* = 8.4 Hz, 1H), 6.77 (s, 1H), 6.63 (d, *J* = 8.4 Hz, 1H), 6.32 (d, *J* = 7.6 Hz, 1H), 5.07–5.04 (m, 1H), 4.65 (t, *J* = 6.8 Hz, 1H), 4.11 (q, *J* = 5.2 Hz, 3H), 4.02 (br t, *J* = 8.4 Hz, 2H), 3.86–3.80 (m, 2H), 2.05–2.00 (m, 1H).

### Synthesis of Intermediates **B2**

To a solution of 2-(2,6-dioxo-3-piperidyl)-5-fluoro-isoindoline-1,3-dione (**31**) (3.0 g, 10.8 mmol) in NMP (22 mL) were added 4-piperidylmethanol (1.9 g, 16.3 mmol) and DIPEA (5.6 g, 43.4 mmol, 7.6 mL) at room temperature. The solution became a yellow suspension. Then, the suspension was stirred at 120 °C for 1 h to give a green solution. After completion, the solution was cooled to room temperature and poured into water (40 mL). Then, the solution was extracted with EA (40 mL × 3) and DCM (40 mL × 3). The organic layers were combined, dried over Na_2_SO_4_, filtered, and concentrated under reduced pressure. The residue was purified by column chromatography [SiO_2_ (120 g), PE/EA = 1:3] and concentrated under reduced pressure to provide 2-(2,6-dioxo-3-piperidyl)-5-[4-(hydroxymethyl)-1-piperidyl]isoindoline-1,3-dione (**32b**) (5.5 g, 82% yield) as a yellow solid. LC–MS: 372, [M + H]^+^, ESI; 1H NMR: (400 MHz, CDCl_3_): δ 8.64 (s, 1H), 7.59 (d, *J* = 8.50 Hz, 1H), 7.20 (d, *J* = 2.13 Hz, 1H), 6.97 (d, *J* = 8.6 Hz, 1H), 4.79–4.93 (m, 1H), 3.90 (br, 2H), 3.45 (d, *J* = 6.3 Hz, 2H), 2.91 (t, *J* = 12.6 Hz, 2H), 2.61–2.75 (m, 2H), 2.10–2.26 (m, 2H), 1.77–1.85 (m, 2), 1.65–1.77 (m, 1H), 1.22–1.36 (m, 2H).

To a yellow solution of 2-(2,6-dioxo-3-piperidyl)-5-[4-(hydroxymethyl)-1-piperidyl]isoindoline-1,3-dione (**32b**) (2.5 g, 4.0 mmol) in DCM (125 mL) was added DMP (3.4 g, 8.1 mmol) at 0 °C. Then, the solution was stirred at room temperature for 2 h. After completion, the mixture was quenched with Na_2_SO_3_ (∼3 g). Then, the suspension was filtered, and the filtrate was extracted with DCM (60 mL × 3). The combined organic layers were dried over Na_2_SO_4_ and concentrated under pressure. The residue was triturated with the solution (PE/EA = 1:1, 30 mL × 2). The solids were collected by filtration. Another crop was obtained by repeating trituration and filtration. In total, 1-[2-(2,6-dioxo-3-piperidyl)-1,3 -dioxo-isoindolin-5-yl]piperidine-4-carbaldehyde (**B2**) (1.7 g, 91% yield) was obtained as a yellow solid. LC–MS: 370.2, [M + H]^+^, ESI; 1H NMR: (400 MHz, DMSO-*d*_6_): δ 11.08 (s, 1H), 9.62 (s, 1H), 7.62–7.69 (m, 1H), 7.31–7.38 (m, 1H), 7.25 (d, *J* = 8.1 Hz, 1H), 4.99–5.15 (m, 1H), 3.94 (d, *J* = 13 Hz, 2H), 3.08–3.23 (m, 2H), 2.83–2.96 (m, 1H), 2.53–2.69 (m, 3H), 1.83–2.07 (m, 4H), 1.45–1.65 (m, 2H).

### Synthesis of Intermediates **B3**

To a solution of 2-(2,6-dioxo-3-piperidyl)-5-fluoro-isoindoline-1,3-dione (**1**) (2.0 g, 7.2 mmol) and azetidin-3-ol (0.79 g, 7.4 mmol) in DMA (20 mL) was added DIPEA (2.1 g, 21.2 mmol, 3.8 mL) at room temperature. Then, the reaction was stirred at 120 °C for 1 h. After completion, 50 mL of water was added to the reaction mixture. The product was extracted with ethyl acetate (100 mL × 3). The combined organic layers were washed with brine (5 mL), dried over Na_2_SO_4_, filtered, and concentrated under reduced pressure. The residue was purified by column chromatography (SiO_2_, petroleum ether/ethyl acetate = 1/1 to ethyl acetate) to give the product 2-(2,6-dioxopiperidin-3-yl)-5-(3-oxoazetidin-1-yl)isoindoline-1,3-dione (**3**) (2.0 g, 82% yield) as a yellow oil. LC–MS (ESI+): 372, [M + H]^+^, ESI+; ^1^H NMR: (400 MHz, DMSO-*d*_6_): δ = 11.19–10.97 (m, 1H), 7.64 (d, *J* = 8.3 Hz, 1H), 6.84–6.76 (m, 1H), 6.72–6.61 (m, 1H), 5.88–5.69 (m, 1H), 5.07 (d, *J* = 12.8 Hz, 1H), 4.70–4.52 (m, 1H), 4.33–4.18 (m, 2H), 3.83–3.66 (m, 2H), 2.63–2.56 (m, 2H), 2.05–1.99 (m, 2H).

To a solution of 2-(2,6-dioxo-3-piperidyl)-5-(3-hydroxyazetidin-1-yl)isoindoline-1,3-dione (**3**) (2.0 g, 6.1 mmol) in DCM (20 mL) was added DMP (3.9 g, 9.1 mmol) at room temperature. Then, the reaction mixture was stirred for 3 h under a N_2_ atmosphere. After completion, water was added to the reaction mixture (50 mL) and it was extracted with ethyl acetate (200 mL × 3) and dichloromethane (200 mL × 3). The combined organic layers were filtered, and the filter cake was washed with ethyl acetate (50 mL). Then, the filtrate was concentrated under reduced pressure to provide 2-(2,6-dioxo-3-piperidyl)-5-(3-oxoazetidin-1-yl)isoindoline-1,3-dione (**B3**) (0.4 g, 10% yield) as a yellow solid. LC–MS (ESI+): 328, [M + H]^+^; ^1^H NMR: (400 MHz, DMSO-*d*_6_): δ 11.16–11.00 (m, 1H), 7.77–7.67 (m, 1H), 7.72–7.67 (m, 1H), 7.06–7.00 (m, 1H), 6.93–6.83 (m, 1H), 5.14–5.04 (m, 1H), 4.94–4.92 (m, 3H), 2.97–2.82 (m, 1H), 2.62 (s, 2H), 2.09–2.00 (m, 1H).

### Synthesis of Intermediates **B4**

To a solution of 3-(5-bromo-1-oxo-isoindolin-2-yl)piperidine-2,6-dione (**4**) (1.0 g, 3.1 mmol) and 3-(dimethoxymethyl)azetidine (0.61 g, 4.6 mmol) in DMSO (10 mL) were added Cs_2_CO_3_ (2.0 g, 6.2 mmol) and Pd-PEPPSI-IPentCl (0.26 g, 0.3 mmol) at room temperature under a N_2_ atmosphere. Then, the mixture was stirred at 80 °C for 16 h. After completion, the solution was poured into 50 mL of water and the mixture was extracted with EtOAc (50 mL × 2). Combined organic layers were dried over Na_2_SO_4_, filtered, and evaporated. The intermediate was purified by flash chromatography on silica gel (PE/EtOAc = 1:2). 3-[5-[3-(dimethoxymethyl)azetidin-1-yl]-1-oxo-isoindolin-2-yl]piperidine-2,6-dione (**5a**) (0.55 g, 47.6% yield) was obtained as an off-white solid. LC–MS (ESI+): 374.2, [M + H]^+^.

To a solution of 3-[5-[3-(dimethoxymethyl)azetidin-1-yl]-1-oxo-isoindolin-2-yl]piperidine- 2,6-dione (**5a**) (0.1 g, 0.27 mmol) was added a solution of TFA (767 mg, 6.7 mmol, 0.5 mL) in DCM (2.5 mL) at 0 °C. Then, the solution was stirred at 40 °C for 2 h. After completion, the solution was concentrated under reduced pressure to give 1-[2-(2,6-dioxo-3-piperidyl)-1 -oxo-isoindolin-5-yl]azetidine-3-carbaldehyde (87 mg, 73% yield, TFA) as a brown oil, which is used in the next step without further purification. LC–MS (ESI+): 328.1, [M + H]^+^.

### Synthesis of Intermediates **B5**

To a solution of 3-(5-bromo-1-oxo-isoindolin-2-yl)piperidine-2,6-dione (**5**) (0.5 g, 1.6 mmol) and 4-(dimethoxymethyl)piperidine (493 mg, 3.1 mmol) in DMSO (10 mL) were added Cs_2_CO_3_ (1.1 g, 3.1 mmol) and Pd-PEPPSI-IPentCl (133 mg, 0.15 mmol) at room temperature under a N_2_ atmosphere. Then, the mixture was stirred at 80 °C for 16 h. After cooling to room temperature, the solution was poured into 60 mL of water and a lot of solids were formed, and then the solids were collected by filtration. Then, the crude product was taken up in 20 mL of ACN and stirred at 25 °C for 1 h. Then, the solid was collected by filtration and dried under high vacuum to provide 3-[5-[4-(dimethoxymethyl)-1- piperidyl]-1-oxo-isoindolin-2- yl]piperidine-2,6-dione (**5b**) (0.6 g, 96% yield) as a gray solid. LC–MS (ESI+): 402.2, [M + H]^+^.

To a solution of 3-[5-[4-(dimethoxymethyl)-1-piperidyl]-1-oxo-isoindolin-2-yl]piperidine- 2,6-dione (**5b**) (0.3 g, 0.75 mmol) in THF (6 mL) was added HCl (2 M, 6 mL) at 25 °C. The solution was stirred at 25 °C for a further 2 h. After completion, the solution was poured into 10 mL of ice–water and the pH was adjusted to ∼7 by progressively adding aq. NaHCO_3_. The solution was extracted with EtOAc (30 mL × 2). Combined organics were dried over Na_2_SO_4_. The mixture was filtered through Celite and the filtrate was evaporated to provide 1-[2-(2,6-dioxo-3-piperidyl)-1-oxo- isoindolin-5-yl]piperidine-4-carbaldehyde (**B5**) (0.2 g, 0.56 mmol, 75% yield) as a brown solid. LC–MS (ESI+): 374, [M + H + H_2_O]^+^; ^1^H NMR: (400 MHz, DMSO-*d*_6_): δ 9.63 (s, 1H), 7.51 (d, *J* = 4 Hz, 1H), 7.05–7.07 (m, 2H), 5.04 (d, *J* = 13.4 Hz, 1H), 4.33 (d, *J* = 16.8 Hz, 1H), 4.20 (d, *J* = 16.8 Hz, 1H), 3.69–3.81 (m, 1H), 2.82–3.75 (m, 3H), 2.56–2.62 (m, 2H), 2.26–2.71 (m, 1H), 1.82–1.93 (m, 3H), 1.50–1.63 (m, 2H).

### Synthesis of Intermediates **6a**, **6b**, and **6c**

#### *tert*-Butyl(*R*)-4-((4-(4-(6-amino-5-(1-(2,6-dichloro-3-fluorophenyl)ethoxy)pyridin-3-yl)-1*H*-pyrazol-1-yl)piperidin-1-yl)methyl)piperidine-1-carboxylate (**6b**)

The mixture of crizotinib (0.23 g, 0.5 mmol), trimethylamine, and *tert*-butyl 4-formylpiperidine-1-carboxylate (0.11 g, 0.5 mmol) in DCM was stirred at rt for 1 h. To the above reaction solution was added Na(OAc)_3_BH (0.16 g, 0.75 mmol) portionwise. Then, the resultant mixture was stirred at rt for 2 h. TLC and LC–MS were used to check that the reaction was finished and concentrated. The residue was purified by flash chromatography on silica gel with hexane-ethyl acetate as eluent to afford 0.18 g (55% yield) of **6b**. LC–MS (ESI+): 647 [M + H]^+^. ^1^H NMR (400 MHz, DMSO-*d*_6_): 7.95 (s, 1H), 7.75 (d, *J* = 2.0 Hz, 1H), 7.56 (m, 1H), 7.52 (d, *J* = 0.8 Hz, 1H), 7.44 (m, 1H), 6.90 (d, *J* = 1.6 Hz, 1H), 6.08 (q, *J* = 6.4 Hz, 1H), 5.62 (s, 2H), 4.08 (m, 1H), 3.92 (d, *J* = 12.4 Hz, 2H), 2.90 (d, *J* = 11.2 Hz, 2H), 2.67 (m, 2H), 2.15 (d, *J* = 6.8 Hz, 2H), 2.06–1.89 (m, 8H), 1.80 (d, *J* = 6.4 Hz, 3H), 1.67 (m, 3H), 1.39 (s, 9H). ^13^C NMR (100 MHz, DMSO-*d*_6_): 156.80 (d, *J* = 246 Hz), 153.89, 149.41, 138.78, 136.84, 135.48, 134.46, 130.51, 128.71 (d, *J* = 4 Hz), 123.46, 121.02 (d, *J* = 19 Hz), 119.06, 117.43 (d, *J* = 14 Hz), 114.49, 78.34, 71.98, 63.55, 58.65, 52.45, 33.05, 32.13, 30.28, 28.09, 18.57.

**6a** and **6c** were synthesized by the above-described method.

#### *tert*-Butyl(*R*)-3-((4-(4-(6-amino-5-(1-(2,6-dichloro-3-fluorophenyl)ethoxy)pyridin-3-yl)-1*H*-pyrazol-1-yl)piperidin-1-yl)methyl)azetidine-1-carboxylate (**6a**)

^1^H NMR (400 MHz, DMSO-*d*_6_): δ 7.94 (s, 1H), 7.75 (d, *J* = 2.0 Hz, 1H), 7.57 (m, 1H), 7.51 (d, *J* = 0.8 Hz, 1H), 7.44 (m, 1H), 6.90 (d, *J* = 1.6 Hz, 1H), 6.07 (q, *J* = 6.4 Hz, 1H), 5.62 (s, 2H), 4.07 (m, 1H), 3.90 (s, 2H), 3.48 (s, 2H), 2.87 (d, *J* = 11.6 Hz, 2H), 2.67 (m, 2H), 2.09 (m, 2H), 1.97–1.81(m, 5H), 1.79 (d, *J* = 6.4 Hz, 3H), 1.37 (s, 9H). ^13^C NMR (100 MHz, DMSO-*d*_6_): 156.77 (d, *J* = 252 Hz), 155.51, 149.42, 138.78, 136.84, 135.48, 134.47, 130.58, 128.71 (d, *J* = 4 Hz), 123.45, 121.02 (d, *J* = 19 Hz), 119.08, 117.52, 117.36 (d, *J* = 13 Hz), 114.48, 78.29, 71.98, 61.39, 58.45, 51.95, 48.56, 45.68, 32.01, 28.06, 26.21, 18.58.

#### *tert*-Butyl(*R*)-4-(2-(4-(4-(6-amino-5-(1-(2,6-dichloro-3-fluorophenyl)ethoxy)pyridin-3-yl)-1*H*-pyrazol-1-yl)piperidin-1-yl)ethyl)piperidine-1-carboxylate (**6c**)

^1^H NMR (400 MHz, DMSO-*d*_6_): 7.94 (s, 1H), 7.74 (d, *J* = 1.6 Hz, 1H), 7.55 (m, 1H), 7.51 (d, *J* = 0.8 Hz, 1H), 7.43 (m, 1H), 6.89 (d, *J* = 1.6 Hz, 1H), 6.08 (q, *J* = 6.4 Hz, 1H), 5.61 (s, 2H), 4.08 (m, 1H), 3.90 (d, *J* = 12.8 Hz, 2H), 2.93 (d, *J* = 11.2 Hz, 2H), 2.67 (m, 2H), 2.55 (m, 2H), 2.32 (m, 2H), 2.17 (m, 2H), 2.05–1.87 (m, 6H), 1.80 (d, *J* = 6.8 Hz, 3H), 1.64 (d, *J* = 12.8 Hz, 3H), 1.38 (s, 9H). ^13^C NMR (100 MHz, DMSO-*d*_6_): 156.83 (d, *J* = 245 Hz), 153.87, 149.43, 138.81, 136.86, 135.48, 134.48, 130.61, 128.73 (d, *J* = 3 Hz), 123.47, 121.05 (d, *J* = 19 Hz), 119.08, 117.55, 117.49 (d, *J* = 13 Hz), 114.51, 78.38, 72.00, 58.67, 55.07, 52.06, 48.59, 48.50, 45.71, 43.31, 33.55, 33.22, 32.33, 32.10, 30.11, 29.00, 28.11, 18.57, 17.22.

### Synthesis of **7a** and **7e**

The mixture of crizotinib (0.12 g, 0.25 mmol), DIEA, and 2-(2,6-dioxopiperidin-3-yl)-5-fluoroisoindoline-1,3-dione (**1**) or 2-(2,6-dioxopiperidin-3-yl)-4-fluoroisoindoline-1,3-dione (**2**) (0.13 g, 0.5 mmol) in NMP was irradiated at 100 °C for 1 h under microwave. TLC and LC–MS were used to check that the reaction was finished and concentrated. The residue was purified by flash chromatography on silica gel with hexane-ethyl acetate as eluent. The crude product was further purified by C18 reverse phase preparative HPLC to afford **7a** and **7e** as a yellow solid.

#### 4-(4-(4-(6-Amino-5-((*R*)-1-(2,6-dichloro-3-fluorophenyl)ethoxy)pyridin-3-yl)-1*H*-pyrazol-1-yl)piperidin-1-yl)-2-(2,6-dioxopiperidin-3-yl)isoindoline-1,3-dione (**7a**)

LC–MS(+): 706. HRMS (ESI) *m*/*z*: calcd, 706.1748 for C_34_H_31_Cl_2_FN_7_O_5_ [M + H]^+^; found, 706.1740; ^1^H NMR (400 MHz, DMSO-*d*_6_): δ 11.06 (s, 1H), 8.03 (s, 1H), 7.78 (s, 1H), 7.77–7.65 (m, 2H), 7.58 (t, *J* = 8.7 Hz, 1H), 7.56 (s, 1H), 7.46–7.36 (m, 4H), 6.93 (d, *J* = 1.6 Hz, 1H), 6.10 (q, *J* = 6.6 Hz, 1H), 5.62 (s, 2H), 5.11 (dd, J = 12.4 and 5.2 Hz, 1H), 4.39 (m, 1H), 3.81 (d, *J* = 11.6 Hz, 3H), 3.17–3.07 (m, 3H), 2.88 (m, 2H), 2.72–2.54 (m, 2H), 2.22–2.03 (m, 7H), 1.81 (d, *J* = 6.8 Hz, 3H). ^13^C NMR (100 MHz, DMSO-*d*_6_): 172.76, 169.97, 167.05, 166.35, 149.61, 149.46, 138.81, 136.88, 135.81, 135.57, 134.68, 133.65, 128.74 (d, *J* = 4 Hz), 124.08, 123.70, 121.05 (d, *J* = 19 Hz), 119.20, 117.42, 116.65, 114.79, 114.59, 72.12, 57.86, 49.81, 48.81, 32.07, 30.93, 22.05, 18.58.

#### 5-(4-(4-(6-Amino-5-((*R*)-1-(2,6-dichloro-3-fluorophenyl)ethoxy)pyridin-3-yl)-1*H*-pyrazol-1-yl)piperidin-1-yl)-2-(2,6-dioxopiperidin-3-yl)isoindoline-1,3-dione (**7e**)

LC–MS(+): 706. HRMS (ESI) *m*/*z*: calcd, 706.1748 for C_34_H_31_Cl_2_FN_7_O_5_ [M + H]^+^; found, 706.1752; ^1^H NMR (400 MHz, DMSO-*d*_6_): δ 11.06 (s, 1H), 7.98 (s, 1H), 7.75 (s, 1H), 7.69 (d, *J* = 8.8 Hz, 1H), 7.56 (m, 1H), 7.43 (t, *J* = 8.8 Hz, 1H), 7.41 (s, 1H), 7.32 (dd, J = 8.8 and 2.4 Hz, 1H), 6.90 (d, *J* = 1.2 Hz, 1H), 6.08 (q, *J* = 6.6 Hz, 1H), 5.62 (s, 2H), 5.07 (dd, J = 12.8 and 5.2 Hz, 1H), 4.48 (m, 1H), 3.22–3.16 (m, 3H), 2.89 (m, 2H), 2.69 (s, 1H), 2.69–2.54 (m, 2H), 2.19–1.88 (m, 5H), 1.80 (d, *J* = 6.4 Hz, 3H). ^13^C NMR (100 MHz, DMSO-*d*_6_): 172.80, 170.07, 167.58, 166.96, 154.62, 149.48, 138.81, 136.86, 135.54, 134.68, 134.08, 128.74 (d, *J* = 4 Hz), 125.06, 123.73, 121.05 (d, *J* = 19 Hz), 119.23, 118.01, 117.97, 117.55, 117.35, 117.32, 114.53, 108.11, 72.04, 58.05, 48.77, 48.49, 46.28, 31.12, 30.98, 30.11, 29.00, 22.19, 18.60, 17.22.

### Synthesis of **7g**

#### 5-(4-((4-(4-(6-Amino-5-((*R*)-1-(2,6-dichloro-3-fluorophenyl)ethoxy)pyridin-3-yl)-1*H*-pyrazol-1-yl)piperidin-1-yl)methyl)piperidin-1-yl)-2-(2,6-dioxopiperidin-3-yl)isoindoline-1,3-dione (**7g**)

The mixture of the amine (0.23 g, 0.5 mmol), triethylamine, and **B2** (0.19 g, 0.5 mmol) in DCM was stirred at rt for 1 h. To the above reaction solution was added Na(OAc)_3_BH (0.16 g, 0.75 mmol) portionwise. Then, the resultant mixture was stirred at rt for 2 h. TLC and LC–MS were used to check that the reaction was finished and concentrated. The residue was purified by flash chromatography on silica gel with hexane-ethyl acetate as eluent to afford 0.15 g (50% yield) of **7g**. LC–MS (ESI+): 803 [M + H]^+^. HRMS (ESI) *m*/*z*: calcd, 803.2639 for C_40_H_42_Cl_2_FN_8_O_5_ [M + H]^+^; found, 803.2636; ^1^H NMR (400 MHz, DMSO-*d*_6_): δ 11.06 (s, 1H), 8.17 (s, 2H), 7.96 (s, 1H), 7.75 (d, *J* = 2.0 Hz, 1H), 7.65 (d, *J* = 8.8 Hz, 1H), 7.57 (m, 1H), 7.52 (d, *J* = 0.4 Hz, 1H), 7.44 (t, *J* = 8.8 Hz, 1H), 7.31 (d, *J* = 2.0 Hz, 1H), 7.23 (dd, J = 8.8 and 2.0 Hz, 1H), 6.91 (d, J = 2.0, Hz, 1H), 6.09 (q, *J* = 6.8 Hz, 1H), 5.62 (s, 2H), 5.06 (dd, J = 12.8 and 5.2 Hz, 1H), 4.10 (m, 1H), 3.06–2.83 (m, 4H), 2.67–2.54 (m, 2H), 2.19 (d, *J* = 6.4 Hz, 2H), 2.09–1.81 (m, 8H), 1.82 (m, 3H), 1.80 (d, *J* = 6.4 Hz, 3H), 1.17–1.15 (m, 2H). ^13^C NMR (100 MHz, DMSO-*d*_6_): 172.76, 170.07, 167.62, 166.59, 156.88 (d, *J* = 238 Hz), 155.01, 149.42, 138.80, 136.85, 135.49, 134.48, 134.03, 131.03(m), 128.71 (d, *J* = 3 Hz), 124.98, 123.48, 121.02 (d, *J* = 19 Hz), 119.08, 117.57, 117.45, 117.31, 114.51, 107.69, 72.00, 63.45, 58.65, 52.45, 48.73, 47.25, 32.89, 32.15, 30.96, 29.62, 22.46, 22.17, 18.58.

### Synthesis of **7b–d**, **7f**, and **7h**

#### 4-(3-((4-(4-(6-Amino-5-((*R*)-1-(2,6-dichloro-3-fluorophenyl)ethoxy)pyridin-3-yl)-1*H*-pyrazol-1-yl)piperidin-1-yl)methyl)azetidin-1-yl)-2-(2,6-dioxopiperidin-3-yl)isoindoline-1,3-dione (**7b**)

To a solution of **6a** (0.24 g, 0.5 mmol) in dioxane was added 3 M HCl in cyclopentyl methyl ether. The mixture was stirred at rt for 1 h. The solvent was removed under vacuum to afford a yellow product without further purification for the next step reaction. To a solution of 2-(2,6-dioxopiperidin-3-yl)-4-fluoroisoindoline-1,3-dione (0.13 g, 0.47 mmol) and DIPEA (1.2 mL, 9.36 mmol) in 3 mL of NMP was added the deBoc-ed product. The mixture was irradiated at 100 °C for 2 h under microwave. After being cooled down, the reaction solution was diluted with satd. NH_4_Cl solution, extracted with DCM, dried over MgSO_4_, concentrated, and purified by a flash column on silica gel with hexane-ethyl acetate as eluent. The crude was further purified by C18 reverse phase preparative HPLC to afford 0.15 g of **7b** (yellow product, 42% yield). LC–MS: 775 HRMS (ESI) *m*/*z*: calcd, 775.2326 for C_38_H_38_Cl_2_FN_8_O_5_ [M + H]^+^; found, 775.2322; ^1^H NMR (400 MHz, DMSO-*d*_6_): δ ^1^H NMR (400 MHz, DMSO-*d*_6_): 11.05 (s, 1H), 8.17 (s, 2H), 7.97 (s, 1H), 7.75 (d, *J* = 1.6 Hz, 1H), 7.61–7.54 (m, 2H), 7.52 (d, *J* = 1 Hz, 1H), 7.44 (t, *J* = 8.8 Hz, 1H), 7.10 (d, *J* = 7.2 Hz, 1H), 6.90 (d, J = 1.6, Hz, 1H), 6.78 (d, *J* = 8.4 Hz, 1H), 6.09 (q, *J* = 6.8 Hz, 1H), 5.62 (s, 2H), 5.04 (dd, J = 12.8 and 5.6 Hz, 1H), 4.31 (m, 2H), 4.10 (m, 1H), 3.87–3.83 (m, 2H), 2.94–2.82 (m, 4H), 2.67–2.53 (m, 2H), 2.14 (m, 2H), 2.03–1.81 (m, 5H), 1.80 (d, *J* = 6.8 Hz, 3H). ^13^C NMR (100 MHz, DMSO-*d*_6_): δ 172.77, 170.00, 167.22, 166.49, 163.32, 156.81 (d, *J* = 246 Hz), 149.43, 148.03, 138.81, 136.86, 135.49, 134.89, 134.48, 133.29, 130.62(m), 128.73 (d, *J* = 2 Hz), 123.48, 121.03 (d, *J* = 19 Hz), 119.84, 119.12, 117.54, 117.45, 117.30, 114.52, 111.52, 109.88, 72.01, 61.56, 58.48, 52.03, 48.63, 32.02, 30.94, 27.08, 22.11, 18.59.

**7c**–**d**, **7f**, and **7g** were synthesized by the above-described method.

#### 4-(4-((4-(4-(6-Amino-5-((*R*)-1-(2,6-dichloro-3-fluorophenyl)ethoxy)pyridin-3-yl)-1*H*-pyrazol-1-yl)piperidin-1-yl)methyl)piperidin-1-yl)-2-(2,6-dioxopiperidin-3-yl)isoindoline-1,3-dione (**7c**)

LC–MS: 775 HRMS (ESI) *m*/*z*: calcd, 803.2639 for C_40_H_42_Cl_2_FN_8_O_5_ [M + H]^+^; found, 803.2633; ^1^H NMR (400 MHz, DMSO-*d*_6_): δ 11.07 (s, 1H), 8.17 (s, 2H), 8.00 (s, 1H), 7.75 (s, 1H), 7.66 (m, 1H), 7.62 (s, 1H), 7.57 (m, 1H), 7.53 (s, 1H), 7.46–7.23 (m, 4H), 6.91 (s, 1H), 6.09 (q, *J* = 6.6 Hz, 1H), 5.97 (s, 1H), 5.08 (dd, J = 12.8 and 5.6 Hz, 1H), 4.39 (m, 1H), 3.70 (d, *J* = 11.8 Hz, 2H), 3.17–2.83 (m, 6H), 2.61–2.53 (m, 1H), 2.31–2.01 (m, 6H), 1.85–1.75 (m, 8H).

#### 4-(4-(2-(4-(4-(6-Amino-5-((*R*)-1-(2,6-dichloro-3-fluorophenyl)ethoxy)pyridin-3-yl)-1*H*-pyrazol-1-yl)piperidin-1-yl)ethyl)piperidin-1-yl)-2-(2,6-dioxopiperidin-3-yl)isoindoline-1,3-dione (**7d**)

LC–MS (ESI+): 775 [M + H]^+^, HRMS (ESI) *m*/*z*: calcd, 817.2796 for C_41_H_44_Cl_2_FN_8_O_5_ [M + H]^+^; found, 817.2301; ^1^H NMR (400 MHz, DMSO-*d*_6_): δ 11.07 (s, 1H), 8.22 (s, 2H), 7.95 (s, 1H), 7.75 (s, 1H), 7.68 (m, 1H), 7.57 (m, 1H), 7.52 (s, 1H), 7.44 (t, *J* = 8.8 Hz, 1H), 7.32 (t, *J* = 7.2 Hz, 2H), 6.90 (d, *J* = 1.6 Hz, 1H), 6.08 (q, *J* = 6.4 Hz, 1H), 5.62 (s, 2H), 5.09 (dd, J = 12.8 and 5.2 Hz, 1H), 4.09 (m, 1H), 3.70 (dd, *J* = 8.4, 5.3 Hz, 2H), 3.17–2.83 (m, 4H), 2.74–2.11 (m, 5H), 2.08–1.88 (m, 5H), 1.80 (d, *J* = 8.0 Hz, 3H), 1.75 (m, 2H), 1.47–1.32 (m, 5H). ^13^C NMR (100 MHz, DMSO-*d*_6_): 173.26, 170.49, 167.59, 166.78, 157.50 (d, *J* = 250 Hz), 150.69, 149.93, 139.31, 137.36, 136.20, 135.99, 135.00, 134.18, 131.03(m), 129.22 (d, *J* = 3 Hz), 124.39, 123.99, 121.53 (d, *J* = 20 Hz), 119.59, 118.03, 117.95, 117.80, 116.78, 115.02, 114.83, 72.52, 59.07, 55.61, 52.51, 51.67, 49.27, 46.59, 33.68, 33.61, 32.50, 31.45, 22.97, 22.57, 19.09.

#### 5-(3-((4-(4-(6-Amino-5-((*R*)-1-(2,6-dichloro-3-fluorophenyl)ethoxy)pyridin-3-yl)-1*H*-pyrazol-1-yl)piperidin-1-yl)methyl)azetidin-1-yl)-2-(2,6-dioxopiperidin-3-yl)isoindoline-1,3-dione (**7f**)

LC–MS: 775 HRMS (ESI) *m*/*z*: calcd, 775.2326 for C_38_H_38_Cl_2_FN_8_O_5_ [M + H]^+^; found, 775.2322; ^1^H NMR (400 MHz, DMSO): δ 11.05 (s, 1H), 8.16 (s, 2H), 7.96 (s, 1H), 7.75 (d, *J* = 2.4 Hz, 1H), 7.63 (dd, J = 8.4 and 2.4 Hz, 1H), 7.57 (m, 1H), 7.53 (s, 1H), 7.44 (t, *J* = 8.8 Hz, 1H), 7.30 (m, 1H), 6.90 (d, *J* = 1.6 Hz, 1H), 6.78 (d, *J* = 2.0 Hz, 1H), 6.65 (dd, J = 8.4 and 2.0 Hz, 1H), 6.09 (q, *J* = 6.6 Hz, 1H), 5.62 (s, 1H), 5.05 (dd, J = 12.8 and 5.6 Hz, 1H), 4.16–4.08 (m, 3H), 3.70 (m, 2H), 3.17–2.84 (m, 4H), 2.67–2.52 (m, 3H), 2.40–2.12 (m, 2H), 2.04–1.88 (m, 4H), 1.80 (d, *J* = 6.6 Hz, 3H). ^13^C NMR (100 MHz, DMSO-*d*_6_): δ 172.76, 170.07, 167.48, 167.16, 163.21, 156.91 (d, *J* = 246 Hz), 155.19, 149.43, 147.99, 138.8, 133.81, 130.70(m), 128.83 (d, *J* = 4 Hz), 124.78, 121.13 (d, *J* = 19 Hz), 114.51, 114.05, 104.31, 72.18, 58.44, 55.72, 52.04, 48.70, 31.99, 30.96, 27.28, 22.46, 22.30, 22.19, 18.59.

#### 5-(4-(2-(4-(4-(6-Amino-5-((*R*)-1-(2,6-dichloro-3-fluorophenyl)ethoxy)pyridin-3-yl)-1*H*-pyrazol-1-yl)piperidin-1-yl)ethyl)piperidin-1-yl)-2-(2,6-dioxopiperidin-3-yl)isoindoline-1,3-dione (**7h**)

LC–MS (ESI+): 775, HRMS (ESI) *m*/*z*: calcd, 817.2796 for C_41_H_44_Cl_2_FN_8_O_5_ [M + H]^+^; found, 817.2301; ^1^H NMR (400 MHz, DMSO-*d*_6_): 11.07 (s, 1H), 8.19 (s, 2H), 7.93 (s, 1H), 7.74 (d, *J* = 1.6 Hz, 1H), 7.64 (d, *J* = 8.8 Hz, 1H), 7.56 (m, 1H), 7.53 (s, 1H), 7.41 (t, *J* = 8.8 Hz, 1H), 7.28 (s, 1H), 7.22 (d, *J* = 8.8 Hz, 1H), 6.89 (d, *J* = 1.6 Hz, 1H), 6.08 (q, *J* = 6.8 Hz, 1H), 5.61 (s, 2H), 5.04 (dd, J = 12.8 and 5.2 Hz, 1H), 4.14 (m, 1H), 3.05–2.84 (m, 4H), 2.61–2.54 (m, 2H), 2.32–1.94 (m, 10H), 1.79 (d, *J* = 6.8 Hz, 3H), 1.76–1.64 (m, 3H), 1.44–1.40 (m, 2H), 1.20–1.17 (m, 2H). ^13^C NMR (100 MHz, DMSO-*d*_6_): 173.56, 172.95, 170.21, 167.76, 167.09, 163.85, 156.91 (d, *J* = 246 Hz), 155.04, 149.52, 138.93, 136.92, 135.52, 134.71, 134.14, 130.70(m), 128.83 (d, *J* = 4 Hz), 125.13, 123.72, 121.13 (d, *J* = 19 Hz), 119.22, 117.69, 117.63, 117.50, 117.40, 114.61, 107.76, 83.51, 72.11, 58.07, 54.86, 51.77, 48.84, 47.52, 33.53, 32.58, 31.53, 31.13, 28.60, 27.56, 26.33, 22.53, 22.29, 18.68.

### Synthesis of **7i**

#### 5-(4-((4-((4-(4-(6-Amino-5-((*R*)-1-(2,6-dichloro-3-fluorophenyl)ethoxy)pyridin-3-yl)-1*H*-pyrazol-1-yl)piperidin-1-yl)methyl)piperidin-1-yl)methyl)piperidin-1-yl)-2-(2,6-dioxopiperidin-3-yl)isoindoline-1,3-dione (**7i**)

To a solution of **6b** (0.24 g, 0.5 mmol) in dioxane was added 3 M HCl in cyclopentyl methyl ether. The mixture was stirred at rt for 1 h. The solvent was removed under vacuum to afford a yellow product without further purification for the next step reaction. The mixture of deBoc-**6b** amine, trimethylamine, and **B2** (0.19 g, 0.5 mmol) in DCM was stirred at rt for 1 h. To the mixture was added Na(OAc)_3_BH (0.16 g, 0.75 mmol) portionwise. Then, the resultant mixture was stirred at rt for 2 h. TLC and LC–MS were used to check that the reaction was finished and concentrated. The residue was purified by flash chromatography on silica gel with hexane-ethyl acetate as eluent. The crude product was further purified by C18 reverse phase preparative HPLC to afford 0.27 g (60% yield) of **7i**. LC–MS: 775 HRMS (ESI) *m*/*z*: calcd, 900.3531 for C_46_H_53_Cl_2_FN_9_O_5_ [M + H]^+^; found, 900.3532; ^1^H NMR (400 MHz, DMSO-*d*_6_): δ ^1^H NMR (400 MHz, DMSO-*d*_6_): 11.07 (s, 1H), 8.20 (s, 2H), 7.95 (s, 1H), 7.75 (d, *J* = 1.6 Hz, 1H), 7.64 (d, *J* = 8.4 Hz, 1H), 7.56 (m, 1H), 7.52 (s, 1H), 7.43 (t, *J* = 8.8 Hz, 1H), 7.30 (d, *J* = 2.0 Hz, 1H), 7.22 (dd, J = 8.8 and 2.0 Hz, 1H), 6.90 (d, J = 1.6, Hz, 1H), 6.08 (q, *J* = 6.8 Hz, 1H), 5.62 (s, 2H), 5.06 (dd, J = 12.8 and 5.6 Hz, 1H), 4.13–4.01 (m, 1H), 2.98–2.83 (m, 7H), 2.68–2.54 (m, 2H), 2.28 (d, *J* = 5.6 Hz, 2H), 2.18 (d, *J* = 6.8 Hz, 2H), 2.09–1.80 (m, 9H), 1.79 (d, *J* = 6.8 Hz, 3H), 1.76–1.70 (m, 4H), 1.54 (s, 1H), 1.22–1.04 (m, 5H). ^13^C NMR (100 MHz, DMSO-*d*_6_): δ 172.77, 170.07, 167.62, 166.95, 163.66, 156.81 (d, *J* = 246 Hz), 154.96, 149.43, 138.81, 136.85, 135.46, 134.50, 134.03, 130.52(m), 128.72 (d, *J* = 3 Hz), 124.98, 123.49, 121.03 (d, *J* = 19 Hz), 119.09, 117.59, 117.52, 117.44, 117.38, 117.29, 114.50, 107.71, 72.00, 63.51, 58.53, 53.33, 52.40, 48.75, 47.15, 46.93, 46.78, 33.00, 32.60, 32.28, 32.01, 30.97, 29.80, 29.51, 29,33, 22.47, 22.19, 18.58.

### Synthesis of **8a−8c** and **8h−8j**

#### 4-(4-(4-((5-Chloro-4-((2-(isopropylsulfonyl)phenyl)amino)pyrimidin-2-yl)amino)-5-isopropoxy-2-methylphenyl)-[1,4′-bipiperidin]-1′-yl)-2-(2,6-dioxopiperidin-3-yl)isoindoline-1,3-dione (**8c**)

Following general procedures A, B, and D, ceritinib (837 mg, 1.5 mmol) was dissolved in 10 mL of dry DCM, followed by adding *tert*-butyl 4-oxopiperidine-1-carboxylate (606 mg, 3 mmol) and triethylamine (0.7 g, 7.5 mmol) at room temperature. After stirring for 10 min, STAB (636 mg, 3 mmol) was added portionwise. After stirring for 5 h, the mixture was concentrated and subjected to silica gel eluent with *n*-hexane/EA = 5:1 to 0:100. 1.10 g (yield, 99%) of *tert*-butyl 4-(4-((5-chloro-4-((2-(isopropylsulfonyl)phenyl)amino)pyrimidin-2-yl)amino)-5-isopropoxy-2-methylphenyl)-[1,4′-bipiperidine]-1′-carboxylate was obtained as a colorless oil. The obtained intermediate was stirred in 5 mL of 2 M HCl in MeOH for 2 h. The solvent was removed in vacuo to provide 1.1 g (yield, 99%) of *N*2-(4-([1,4′-bipiperidin]-4-yl)-2-isopropoxy-5-methylphenyl)-5-chloro-*N*4-(2-(isopropylsulfonyl)phenyl)pyrimidine-2,4-diamine HCl salt as a white solid. A mixture of *N*2-(4-([1,4′-bipiperidin]-4-yl)-2-isopropoxy-5-methylphenyl)-5-chloro-*N*4-(2-(isopropylsulfonyl)phenyl)pyrimidine-2,4-diamine HCl salt (100 mg, 0.15 mmol), DIEA (98 mg, 0.75 mmol), 2-(2,6-dioxopiperidin-3-yl)-5-fluoroisoindoline-1,3-dione (83 mg, 0.3 mmol), and 2 mL of NMP was stirred in a microwave reactor at 120 °C for 1 h. After cooling to rt, the mixture was subjected to silica gel eluent with *n*-hexane/EA = 5:1 to 0:100 and then EA/MeOH = 8:2. The crude product was further purified by C18 reverse phase preparative HPLC to give 33 mg (yield, 25%) of **8c** as a yellow solid. HRMS (ESI) *m*/*z*: calcd, 897.3524 for C_46_H_53_ClN_8_O_7_S [M + H]^+^; found, 897.3533; ^1^H NMR (400 MHz, DMSO-*d*_6_): δ 11.10 (s, 1H), 9.46 (s, 1H), 8.47 (d, *J* = 8.4 Hz, 1H), 8.25 (s, 1H), 8.06 (s, 1H), 7.84 (dd, *J* = 8.0, 1.7 Hz, 1H), 7.66 (dt, *J* = 24.4, 7.9 Hz, 3H), 7.52 (s, 1H), 7.36 (q, *J* = 6.8 Hz, 3H), 6.86 (s, 1H), 5.10 (dd, *J* = 12.9, 5.5 Hz, 1H), 4.59 (p, *J* = 6.1 Hz, 1H), 3.78 (d, *J* = 11.4 Hz, 2H), 3.48–3.40 (m, 5H), 3.04 (d, *J* = 10.3 Hz, 3H), 2.89 (q, *J* = 10.6 Hz, 4H), 2.62 (d, *J* = 3.6 Hz, 2H), 2.58 (s, 2H), 2.56 (s, 1H), 2.36 (d, *J* = 12.1 Hz, 2H), 2.13 (s, 3H), 1.89 (d, *J* = 12.0 Hz, 2H), 1.11–1.25 (d, 12H).

**8a**, **8b**, **8h**, **8i**, **8j** were synthesized similarly to **8c** with corresponding starting materials.

#### 4-((2-(4-(4-((5-Chloro-4-((2-(isopropylsulfonyl)phenyl)amino)pyrimidin-2-yl)amino)-5-isopropoxy-2-methylphenyl)piperidin-1-yl)ethyl)amino)-2-(2,6-dioxopiperidin-3-yl)isoindoline-1,3-dione (**8a**)

Yellow solid. HRMS (ESI) *m*/*z*: calcd, 857.3211 for C_43_H_49_ClN_8_O_7_S [M + H]^+^; found, 857.3214; ^1^H NMR (400 MHz, DMSO-*d*_6_): δ 11.09 (s, 1H), 9.46 (s, 1H), 8.47 (d, *J* = 8.3 Hz, 1H), 8.28 (d, *J* = 26.6 Hz, 2H), 8.07 (s, 1H), 7.84 (dd, *J* = 8.1, 1.6 Hz, 1H), 7.74–7.44 (m, 3H), 7.35 (t, *J* = 7.3 Hz, 1H), 7.12 (d, *J* = 8.5 Hz, 1H), 7.02 (dd, *J* = 16.0, 6.8 Hz, 1H), 6.80 (d, *J* = 21.8 Hz, 2H), 5.05 (dd, *J* = 12.7, 5.3 Hz, 1H), 4.60–4.38 (m, 1H), 3.05 (d, *J* = 10.8 Hz, 5H), 2.87 (s, 3H), 2.50 (p, *J* = 1.9 Hz, 3H), 2.13 (s, 7H), 1.69 (s, 2H), 1.32–1.04 (m, 12H).

#### 4-((3-(4-(4-((5-Chloro-4-((2-(isopropylsulfonyl)phenyl)amino)pyrimidin-2-yl)amino)-5-isopropoxy-2-methylphenyl)piperidin-1-yl)propyl)amino)-2-(2,6-dioxopiperidin-3-yl)isoindoline-1,3-dione (**8b**)

Yellow solid. HRMS (ESI) *m*/*z*: calcd, 871.3368 for C_44_H_51_ClN_8_O_7_S [M + H]^+^; found, 871.3378; ^1^H NMR (400 MHz, DMSO-*d*_6_): δ 11.09 (s, 1H), 9.46 (s, 1H), 8.46 (d, *J* = 8.3 Hz, 1H), 8.25 (s, 1H), 8.06 (s, 1H), 7.83 (dd, *J* = 7.9, 1.7 Hz, 1H), 7.66–7.55 (m, 2H), 7.51 (s, 1H), 7.39–7.29 (m, 1H), 7.16 (d, *J* = 8.6 Hz, 1H), 7.03 (d, *J* = 7.0 Hz, 1H), 6.86 (s, 1H), 6.70 (t, *J* = 6.0 Hz, 1H), 5.08–4.99 (m, 1H), 4.54 (p, *J* = 6.1 Hz, 1H), 3.48–3.35 (m, 3H), 3.04 (d, *J* = 10.6 Hz, 2H), 2.86–3.00 (m, 2H), 2.66–2.52 (m, 3H), 2.49–2.43 (m, 4H), 2.12 (s, 3H), 2.01–2.08 (m, 3H), 1.82–1.70 (m, 3H), 1.69 (s, 2H), 1.22 (d, *J* = 6.8 Hz, 6H), 1.16 (d, *J* = 6.8 Hz, 6H).

#### 5-((2-(4-(4-((5-Chloro-4-((2-(isopropylsulfonyl)phenyl)amino)pyrimidin-2-yl)amino)-5-isopropoxy-2-methylphenyl)piperidin-1-yl)ethyl)amino)-2-(2,6-dioxopiperidin-3-yl)isoindoline-1,3-dione (**8h**)

Yellow solid. HRMS (ESI) *m*/*z*: calcd, 857.3211 for C_43_H_49_ClN_8_O_7_S [M + H]^+^; found, 857.3218; ^1^H NMR (400 MHz, DMSO-*d*_6_): δ 11.08 (s, 1H), 9.46 (s, 1H), 8.46 (d, *J* = 8.3 Hz, 1H), 8.27 (d, 2H), 8.07 (s, 1H), 7.84 (dd, *J* = 8.1, 1.6 Hz, 1H), 7.74–7.44 (m, 3H), 7.35 (t, *J* = 7.3 Hz, 1H), 7.12 (d, *J* = 8.5 Hz, 1H), 7.02 (dd, *J* = 16.0, 6.8 Hz, 1H), 6.80 (d, *J* = 21.8 Hz, 2H), 5.05 (dd, *J* = 12.7, 5.3 Hz, 1H), 4.60–4.38 (m, 1H), 3.05 (d, *J* = 10.8 Hz, 5H), 2.87 (s, 3H), 2.50 (p, *J* = 1.9 Hz, 3H), 2.13 (s, 7H), 1.69 (s, 2H), 1.20 (d, *J* = 6.0 Hz, 6H), 1.17 (d, *J* = 6.8 Hz, 6H).

#### 5-((3-(4-(4-((5-Chloro-4-((2-(isopropylsulfonyl)phenyl)amino)pyrimidin-2-yl)amino)-5-isopropoxy-2-methylphenyl)piperidin-1-yl)propyl)amino)-2-(2,6-dioxopiperidin-3-yl)isoindoline-1,3-dione (**8i**)

Yellow solid. HRMS (ESI) *m*/*z*: calcd, 871.3368 for C_44_H_51_ClN_8_O_7_S [M + H]^+^; found, 871.3379; ^1^H NMR (400 MHz, DMSO-*d*_6_): δ 11.07 (s, 1H), 9.46 (s, 1H), 8.47 (d, *J* = 8.5 Hz, 1H), 8.30 (s, 1H), 8.25 (s, 2H), 8.06 (s, 1H), 7.87–7.80 (m, 2H), 7.68–7.49 (m, 4H), 7.40–7.31 (m, 2H), 7.22–7.08 (m, 2H), 6.99 (d, *J* = 2.1 Hz, 1H), 6.91–6.76 (m, 3H), 5.02 (dd, *J* = 12.9, 5.3 Hz, 1H), 4.58 (q, *J* = 6.1 Hz, 1H), 3.01 (d, *J* = 10.8 Hz, 4H), 2.85 (d, *J* = 13.4 Hz, 3H), 2.57 (d, *J* = 17.8 Hz, 7H), 2.43 (t, *J* = 7.0 Hz, 4H), 2.14 (s, 1H), 2.12 (s, 3H), 2.00 (dd, *J* = 15.3, 9.0 Hz, 4H), 1.80–1.70 (m, 4H), 1.68 (s, 3H), 1.22 (d, *J* = 6.0 Hz, 6H), 1.16 (d, *J* = 6.8 Hz, 6H).

#### 5-(4-(4-((5-Chloro-4-((2-(isopropylsulfonyl)phenyl)amino)pyrimidin-2-yl)amino)-5-isopropoxy-2-methylphenyl)-[1,4′-bipiperidin]-1′-yl)-2-(2,6-dioxopiperidin-3-yl)isoindoline-1,3-dione (**8j**)

Yellow solid. HRMS (ESI) *m*/*z*: calcd, 897.3524 for C_46_H_53_ClN_8_O_7_S [M + H]^+^; found, 897.3537; ^1^H NMR (400 MHz, DMSO-*d*_6_): δ 11.08 (s, 1H), 9.45 (s, 1H), 8.46 (d, *J* = 8.4 Hz, 1H), 8.24 (s, 1H), 8.05 (s, 1H), 7.83 (dd, *J* = 8.0, 1.7 Hz, 1H), 7.70–7.57 (m, 2H), 7.50 (s, 1H), 7.40–7.31 (m, 2H), 7.26 (dd, *J* = 8.7, 2.3 Hz, 1H), 6.83 (s, 1H), 5.07 (d, *J* = 12.9 1H), 4.57 (p, *J* = 6.0 Hz, 1H), 4.13 (s, 1H), 4.09 (s, 1H), 4.08–3.99 (m, 1H), 3.43 (p, *J* = 6.8 Hz, 3H), 3.01 (s, 1H), 3.00–2.82 (m, 4H), 2.65–2.52 (m, 3H), 2.32 (s, 1H), 2.20–2.12 (m, 1H), 2.11 (s, 2H), 2.06–1.97 (m, 2H), 1.87 (d, *J* = 12.1 Hz, 2H), 1.67 (s, 2H), 1.54 (d, *J* = 9.3 Hz, 3H), 1.39–1.29 (m, 1H), 1.29–1.23 (m, 1H), 1.21 (d, *J* = 6.0 Hz, 6H), 1.15 (d, *J* = 6.8 Hz, 6H).

### Synthesis of **8d−8g** and **8k−8t**

#### 5-(3-((4-(4-((5-Chloro-4-((2-(isopropylsulfonyl)phenyl)amino)pyrimidin-2-yl)amino)-5-isopropoxy-2-methylphenyl)-[1,4′-bipiperidin]-1′-yl)methyl)azetidin-1-yl)-2-(2,6-dioxopiperidin-3-yl)isoindoline-1,3-dione (**8n**)

Following general procedures A, B, and D, *N*2-(4-([1,4′-bipiperidin]-4-yl)-2-isopropoxy-5-methylphenyl)-5-chloro-*N*4-(2-isopropylsulfonyl)phenyl)pyrimidine-2,4-diamine (640 mg, 1 mmol) was dissolved in 5 mL of dry DCM, followed by adding *tert*-butyl 3-formylazetidine-1-carboxylate (370 mg, 2 mmol) and triethylamine (0.50 g, 5 mmol) at room temperature. After stirring for 10 min, STAB (424 mg, 2 mmol) was added portionwise. After stirring for 2 h, the mixture was concentrated and subjected to silica gel eluent with *n*-hexane/EA = 5:1 to 0:100. 760 mg (yield, 94%) of *tert*-butyl 3-((4-(4-((5-chloro-4-((2-(isopropylsulfonyl)phenyl)amino)pyrimidin-2-yl)amino)-5-isopropoxy-2-methylphenyl)-[1,4′-bipiperidin]-1′-yl)methyl)azetidine-1-carboxylate was obtained as a colorless oil. The above-obtained intermediate was treated with 2 M HCl in MeOH and stirred for 2 h. The solvent was removed in vacuo to provide 660 mg (yield, HCl salt, 99%) of *N*2-(4-(1′-(azetidin-3-ylmethyl)-[1,4′-bipiperidin]-4-yl)-2-isopropoxy-5-methylphenyl)-5-chloro-*N*4-(2-(isopropylsulfonyl)phenyl)pyrimidine-2,4-diamine as a white solid. A mixture of *N*2-(4-(1′-(azetidin-3-ylmethyl)-[1,4′-bipiperidin]-4-yl)-2-isopropoxy-5-methylphenyl)-5-chloro-*N*4-(2-(isopropylsulfonyl)phenyl)pyrimidine-2,4-diamine (106 mg, 0.15 mmol), DIEA (98 mg, 0.75 mmol), 2-(2,6-dioxopiperidin-3-yl)-5-fluoroisoindoline-1,3-dione (83 mg, 0.3 mmol), and 2 mL of NMP was stirred in a microwave reactor at 120 °C for 1 h. After cooling to rt, the mixture was subjected to silica gel eluent with *n*-hexane/EA = 5:1 to 0:100 and then EA/MeOH = 8:2. The crude product was further purified by C18 reverse phase preparative HPLC to give 23 mg (yield, 16%) of **8n** as a yellow solid. HRMS (ESI) *m*/*z*: calcd, 966.4103 for C_50_H_60_ClN_9_O_7_S [M + H]^+^; found, 966.4099; ^1^H NMR (400 MHz, DMSO-*d*_6_): δ 11.07 (s, 1H), 9.46 (s, 1H), 8.47 (d, *J* = 8.4 Hz, 1H), 8.25 (s, 1H), 8.05 (s, 1H), 7.84 (dd, *J* = 8.0, 1.6 Hz, 1H), 7.62 (t, *J* = 8.0 Hz, 2H), 7.51 (s, 2H), 7.41–7.29 (m, 1H), 6.91–6.72 (m, 2H), 6.72–6.49 (m, 1H), 5.05 (dd, *J* = 12.8, 5.4 Hz, 1H), 4.58 (p, *J* = 6.0 Hz, 1H), 4.12 (q, *J* = 6.4 Hz, 2H), 3.76–3.60 (m, 3H), 3.46–3.41 (m, 5H), 3.02 (d, *J* = 10.9 Hz, 4H), 2.96–2.80 (m, 4H), 2.32 (s, 4H), 2.12 (s, 4H), 2.06–1.89 (m, 3H), 1.75 (d, *J* = 11.7 Hz, 2H), 1.65 (d, *J* = 18.7 Hz, 4H), 1.48 (q, *J* = 11.4 Hz, 2H), 1.22 (d, *J* = 6.0 Hz, 6H), 1.16 (d, *J* = 6.8 Hz, 6H).

**8d**, **8e**, **8f**, **8g**, **8k**, **8l**, **8m**, **8o**, **8p**, **8q**, **8r**, **8s**, and **8t** were synthesized similarly to **8n** with corresponding starting materials.

#### 4-((2-(4-(4-((5-Chloro-4-((2-(isopropylsulfonyl)phenyl)amino)pyrimidin-2-yl)amino)-5-isopropoxy-2-methylphenyl)-[1,4′-bipiperidin]-1′-yl)ethyl)amino)-2-(2,6-dioxopiperidin-3-yl)isoindoline-1,3-dione (**8d**)

Yellow solid. HRMS (ESI) *m*/*z*: calcd, 940.3946 for C_48_H_58_ClN_9_O_7_S [M + H]^+^; found, 940.3956; ^1^H NMR (400 MHz, DMSO-*d*_6_): δ 11.10 (s, 1H), 9.46 (s, 1H), 8.46 (d, *J* = 8.5 Hz, 1H), 8.25 (d, *J* = 3.0 Hz, 1H), 8.18 (s, 1H), 8.07 (s, 1H), 7.83 (d, *J* = 8.2 Hz, 2H), 7.61 (d, *J* = 8.4 Hz, 2H), 7.55–7.45 (m, 2H), 7.35 (t, *J* = 7.7 Hz, 2H), 7.10 (d, *J* = 8.5 Hz, 1H), 7.07–6.97 (m, 2H), 6.84 (s, 1H), 6.75 (d, *J* = 16.8 Hz, 1H), 5.04 (s, 1H), 4.56 (s, 1H), 3.00 (s, 3H), 2.87 (d, *J* = 13.3 Hz, 4H), 2.56 (s, 5H), 2.13 (d, *J* = 9.7 Hz, 4H), 2.02 (s, 2H), 1.83 (s, 2H), 1.72 (s, 2H), 1.54 (s, 1H), 1.22 (t, *J* = 5.8 Hz, 6H), 1.16 (d, *J* = 6.8 Hz, 6H).

#### 4-((3-(4-(4-((5-Chloro-4-((2-(isopropylsulfonyl)phenyl)amino)pyrimidin-2-yl)amino)-5-isopropoxy-2-methylphenyl)-[1,4′-bipiperidin]-1′-yl)propyl)amino)-2-(2,6-dioxopiperidin-3-yl)isoindoline-1,3-dione (**8e**)

Yellow solid. HRMS (ESI) *m*/*z*: calcd, 954.4103 for C_49_H_60_ClN_9_O_7_S [M + H]^+^; found, 954.4112; ^1^H NMR (400 MHz, DMSO-*d*_6_): δ 11.10 (s, 1H), 9.46 (s, 1H), 8.47 (d, *J* = 8.3 Hz, 1H), 8.23 (d, *J* = 11.5 Hz, 2H), 8.08 (d, *J* = 21.8 Hz, 1H), 7.84 (d, *J* = 8.0 Hz, 1H), 7.71–7.55 (m, 1H), 7.54–7.42 (m, 1H), 7.36 (t, *J* = 7.7 Hz, 1H), 7.12 (d, *J* = 8.7 Hz, 1H), 7.07–6.94 (m, 1H), 6.94–6.71 (m, 1H), 5.05 (dd, *J* = 12.4, 5.4 Hz, 1H), 4.57 (q, *J* = 6.1 Hz, 1H), 3.14–2.80 (m, 10H), 2.43–2.59 (m, 5H), 2.23–1.98 (m, 4H), 1.91–1.97 (m, 3H), 1.73–1.90 (m, 8H), 1.21 (dd, *J* = 6.0 Hz, 6H), 1.16 (d, *J* = 6.8 Hz, 6H).

#### 4-(3-(4-(4-((5-Chloro-4-((2-(isopropylsulfonyl)phenyl)amino)pyrimidin-2-yl)amino)-5-isopropoxy-2-methylphenyl)-[1,4′-bipiperidin]-1′-yl)azetidin-1-yl)-2-(2,6-dioxopiperidin-3-yl)isoindoline-1,3-dione (**8f**)

Yellow solid. HRMS (ESI) *m*/*z*: calcd, 952.3946 for C_49_H_58_ClN_9_O_7_S [M + H]^+^; found, 952.3926; ^1^H NMR (400 MHz, DMSO-*d*_6_): δ 11.08 (s, 1H), 9.46 (s, 1H), 8.46 (d, *J* = 8.4 Hz, 1H), 8.22 (d, *J* = 19.1 Hz, 2H), 8.05 (s, 1H), 7.83 (dd, *J* = 8.0, 1.7 Hz, 1H), 7.74–7.54 (m, 2H), 7.50 (s, 1H), 7.35 (t, *J* = 7.6 Hz, 1H), 7.13 (d, *J* = 7.0 Hz, 1H), 6.93–6.70 (m, 2H), 5.05 (dd, *J* = 12.7, 5.5 Hz, 1H), 4.57 (p, *J* = 6.0 Hz, 1H), 4.28 (s, 2H), 3.94 (dd, *J* = 9.5, 5.2 Hz, 2H), 3.19 (q, *J* = 6.2 Hz, 3H), 3.01 (d, *J* = 10.5 Hz, 3H), 2.95–2.82 (m, 3H), 2.32 (d, *J* = 12.0 Hz, 4H), 2.11 (s, 3H), 2.06–1.94 (m, 1H), 1.83 (dd, *J* = 27.1, 13.6 Hz, 4H), 1.64 (d, *J* = 20.1 Hz, 4H), 1.48 (d, *J* = 12.0 Hz, 2H), 1.21 (d, *J* = 6.0 Hz, 6H), 1.16 (d, *J* = 6.8 Hz, 6H).

#### 4-(3-((4-(4-((5-Chloro-4-((2-(isopropylsulfonyl)phenyl)amino)pyrimidin-2-yl)amino)-5-isopropoxy-2-methylphenyl)-[1,4′-bipiperidin]-1′-yl)methyl)azetidin-1-yl)-2-(2,6-dioxopiperidin-3-yl)isoindoline-1,3-dione (**8g**)

Yellow solid. HRMS (ESI) *m*/*z*: calcd, 966.4103 for C_50_H_60_ClN_9_O_7_S [M + H]^+^; found, 966.4113; ^1^H NMR (400 MHz, DMSO-*d*_6_): δ 11.07 (s, 1H), 9.46 (s, 1H), 8.47 (d, *J* = 8.4 Hz, 1H), 8.25 (s, 1H), 8.20 (s, 1H), 8.05 (s, 1H), 7.84 (dd, *J* = 8.0, 1.6 Hz, 1H), 7.66–7.48 (m, 3H), 7.40–7.31 (m, 1H), 7.10 (d, *J* = 7.0 Hz, 1H), 6.84 (s, 1H), 6.78 (d, *J* = 8.5 Hz, 1H), 5.04 (dd, *J* = 12.7, 5.5 Hz, 1H), 4.57 (p, *J* = 6.0 Hz, 1H), 4.29 (s, 1H), 3.81 (d, *J* = 7.9 Hz, 2H), 3.47 (s, 4H), 3.03 (d, *J* = 10.5 Hz, 2H), 2.94–2.82 (m, 4H), 2.51 (s, 8H), 2.33 (d, *J* = 11.4 Hz, 3H), 2.12 (s, 3H), 2.04–1.90 (m, 3H), 1.76 (d, *J* = 11.7 Hz, 2H), 1.70 (s, 1H), 1.68 (s, 2H), 1.49 (q, *J* = 11.6 Hz, 2H), 1.22 (d, *J* = 6.0 Hz, 5H), 1.16 (d, *J* = 6.8 Hz, 6H).

#### 5-((2-(4-(4-((5-Chloro-4-((2-(isopropylsulfonyl)phenyl)amino)pyrimidin-2-yl)amino)-5-isopropoxy-2-methylphenyl)-[1,4′-bipiperidin]-1′-yl)ethyl)amino)-2-(2,6-dioxopiperidin-3-yl)isoindoline-1,3-dione (**8k**)

Yellow solid. HRMS (ESI) *m*/*z*: calcd, 940.3946 for C_48_H_58_ClN_9_O_7_S [M + H]^+^; found, 940.3960; ^1^H NMR (400 MHz, DMSO-*d*_6_): δ 11.06 (s, 1H), 9.45 (s, 1H), 8.46 (d, *J* = 8.3 Hz, 2H), 8.25 (d, *J* = 9.5 Hz, 4H), 8.08 (d, *J* = 21.9 Hz, 2H), 7.90–7.75 (m, 2H), 7.66–7.44 (m, 4H), 7.35 (t, *J* = 7.7 Hz, 2H), 7.09–6.92 (m, 2H), 6.92–6.72 (m, 3H), 5.03 (dd, *J* = 12.8, 5.6 Hz, 1H), 4.66–4.47 (m, 1H), 3.09–2.78 (m, 10H), 2.42–2.21 (m, 8H), 2.13 (d, *J* = 12.1 Hz, 7H), 2.00 (s, 3H), 1.90–1.41 (m, 8H), 1.22 (t, *J* = 6.5 Hz, 6H), 1.16 (d, *J* = 6.7 Hz, 6H).

#### 5-((3-(4-(4-((5-Chloro-4-((2-(isopropylsulfonyl)phenyl)amino)pyrimidin-2-yl)amino)-5-isopropoxy-2-methylphenyl)-[1,4′-bipiperidin]-1′-yl)propyl)amino)-2-(2,6-dioxopiperidin-3-yl)isoindoline-1,3-dione (**8l**)

Yellow solid. HRMS (ESI) *m*/*z*: calcd, 954.4103 for C_49_H_60_ClN_9_O_7_S [M + H]^+^; found, 954.4116; ^1^H NMR (400 MHz, DMSO-*d*_6_): δ 11.09 (s, 1H), 9.47 (s, 1H), 8.47 (d, *J* = 8.3 Hz, 1H), 8.23 (d, *J* = 11.5 Hz, 2H), 8.08 (d, *J* = 21.8 Hz, 1H), 7.84 (d, *J* = 8.0 Hz, 1H), 7.71–7.55 (m, 1H), 7.54–7.42 (m, 1H), 7.36 (t, *J* = 7.7 Hz, 1H), 7.12 (d, *J* = 8.7 Hz, 1H), 7.07–6.94 (m, 1H), 6.94–6.71 (m, 1H), 5.05 (dd, *J* = 12.4, 5.4 Hz, 1H), 4.57 (q, *J* = 6.1 Hz, 1H), 3.14–2.80 (m, 10H), 2.43–2.59 (m, 5H), 2.23–1.98 (m, 4H), 1.91–1.97 (m, 3H), 1.72–1.92 (m, 8H), 1.22 (d, *J* = 6.0 Hz, 6H), 1.15 (d, *J* = 6.8 Hz, 6H).

#### 5-(3-(4-(4-((5-Chloro-4-((2-(isopropylsulfonyl)phenyl)amino)pyrimidin-2-yl)amino)-5-isopropoxy-2-methylphenyl)-[1,4′-bipiperidin]-1′-yl)azetidin-1-yl)-2-(2,6-dioxopiperidin-3-yl)isoindoline-1,3-dione (**8m**)

Yellow solid. HRMS (ESI) *m*/*z*: calcd, 952.3946 for C_49_H_58_ClN_9_O_7_S [M + H]^+^; found, 952.3930; ^1^H NMR (400 MHz, DMSO-*d*_6_): δ 11.07 (s, 1H), 9.46 (s, 1H), 8.46 (d, *J* = 8.4 Hz, 1H), 8.25 (s, 1H), 8.17 (s, 1H), 8.06 (s, 1H), 7.84 (dd, *J* = 8.0, 1.6 Hz, 1H), 7.69–7.57 (m, 2H), 7.51 (s, 1H), 7.39–7.31 (m, 1H), 6.85–6.77 (m, 2H), 6.65 (d, *J* = 8.4 Hz, 1H), 5.06 (dd, *J* = 12.9, 5.4 Hz, 1H), 4.55 (h, *J* = 6.0 Hz, 1H), 4.10 (t, *J* = 7.7 Hz, 2H), 3.83–3.92 (m, 3H), 3.45 (h, *J* = 6.8 Hz, 8H), 3.36–3.27 (m, 4H), 2.95–2.81 (m, 4H), 2.51 (s, 6H), 2.50 (d, *J* = 1.8 Hz, 7H), 2.12 (s, 3H), 2.05–1.97 (m, 1H), 1.88 (t, *J* = 12.0 Hz, 4H), 1.73 (t, *J* = 5.0 Hz, 4H), 1.55 (t, *J* = 11.8 Hz, 2H), 1.22 (d, *J* = 6.0 Hz, 6H), 1.15 (d, *J* = 6.8 Hz, 6H).

#### 5-(3-(4-(4-((5-Chloro-4-((2-(isopropylsulfonyl)phenyl)amino)pyrimidin-2-yl)amino)-5-isopropoxy-2-methylphenyl)piperidin-1-yl)-[1,3′-biazetidin]-1′-yl)-2-(2,6-dioxopiperidin-3-yl)isoindoline-1,3-dione (**8o**)

Yellow solid. HRMS (ESI) *m*/*z*: calcd, 924.3633 for C_47_H_54_ClN_9_O_7_S [M + H]^+^; found, 924.3629; ^1^H NMR (400 MHz, DMSO-*d*_6_): δ 11.07 (s, 1H), 9.45 (s, 1H), 8.46 (d, *J* = 8.5 Hz, 1H), 8.24 (d, *J* = 6.3 Hz, 1H), 8.05 (s, 1H), 7.83 (dd, *J* = 8.1, 1.7 Hz, 1H), 7.73–7.56 (m, 1H), 7.51 (s, 1H), 7.35 (t, *J* = 7.7 Hz, 1H), 6.93–6.72 (m, 1H), 6.66 (d, *J* = 8.4 Hz, 1H), 5.05 (dd, *J* = 12.9, 5.5 Hz, 1H), 4.59 (p, *J* = 6.1 Hz, 1H), 4.05 (t, *J* = 7.8 Hz, 2H), 3.80 (d, *J* = 9.0 Hz, 2H), 2.92 (d, *J* = 10.1 Hz, 2H), 2.84 (d, *J* = 8.4 Hz, 2H), 2.11 (s, 2H), 1.95 (d, 2H), 1.65 (s, 3H), 1.22 (d, *J* = 6.0 Hz, 6H), 1.16 (d, *J* = 6.7 Hz, 6H).

#### 5-(4-(3-(4-(4-((5-Chloro-4-((2-(isopropylsulfonyl)phenyl)amino)pyrimidin-2-yl)amino)-5-isopropoxy-2-methylphenyl)piperidin-1-yl)azetidin-1-yl)piperidin-1-yl)-2-(2,6-dioxopiperidin-3-yl)isoindoline-1,3-dione (**8p**)

Yellow solid. HRMS (ESI) *m*/*z*: calcd, 952.3946 for C_49_H_58_ClN_9_O_7_S [M + H]^+^; found, 952.3928; ^1^H NMR (400 MHz, DMSO-*d*_6_): δ 11.07 (s, 1H), 9.45 (s, 1H), 8.46 (d, *J* = 8.3 Hz, 1H), 8.27 (d, *J* = 19.3 Hz, 1H), 8.05 (s, 1H), 7.94–7.75 (m, 1H), 7.75–7.58 (m, 1H), 7.50 (s, 1H), 7.41–7.12 (m, 2H), 6.85 (s, 1H), 5.06 (dd, *J* = 12.9, 5.4 Hz, 1H), 4.71–4.39 (m, 1H), 3.82 (s, 2H), 3.14 (s, 2H), 2.94–2.71 (m, 4H), 2.29 (s, 2H), 2.11 (s, 3H), 1.88 (s, 2H), 1.68 (d, *J* = 24.7 Hz, 3H), 1.22 (d, *J* = 6.0 Hz, 6H), 1.16 (d, *J* = 6.8 Hz, 6H).

#### 5-(3-((3-(4-(4-((5-Chloro-4-((2-(isopropylsulfonyl)phenyl)amino)pyrimidin-2-yl)amino)-5-isopropoxy-2-methylphenyl)piperidin-1-yl)azetidin-1-yl)methyl)azetidin-1-yl)-2-(2,6-dioxopiperidin-3-yl)isoindoline-1,3-dione (**8q**)

Yellow solid. HRMS (ESI) *m*/*z*: calcd, 938.3790 for C_48_H_56_ClN_9_O_7_S [M + H]^+^; found, 938.3789; ^1^H NMR (400 MHz, DMSO-*d*_6_): δ 11.06 (s, 1H), 9.45 (s, 1H), 8.46 (d, *J* = 8.5 Hz, 1H), 8.24 (s, 1H), 8.18 (s, 1H), 8.04 (s, 1H), 7.83 (d, *J* = 7.9 Hz, 1H), 7.63 (d, *J* = 8.3 Hz, 2H), 7.50 (s, 1H), 7.35 (t, *J* = 7.7 Hz, 1H), 6.84 (s, 1H), 6.78–6.73 (m, 1H), 6.62 (dd, *J* = 8.4, 2.1 Hz, 1H), 5.05 (dd, *J* = 12.8, 5.5 Hz, 1H), 4.58 (p, *J* = 6.1 Hz, 1H), 4.07 (t, *J* = 8.0 Hz, 2H), 3.80 (s, 1H), 3.69 (dd, *J* = 8.4, 5.0 Hz, 6H), 3.47 (s, 6H), 2.92–2.78 (m, 6H), 2.70 (d, *J* = 7.0 Hz, 2H), 2.61–2.51 (m, 4H), 2.10 (s, 3H), 2.00 (d, *J* = 12.4 Hz, 1H), 1.88 (s, 1H), 1.65 (s, 3H), 1.22 (d, *J* = 6.0 Hz, 6H), 1.16 (d, *J* = 6.8 Hz, 6H).

#### 5-(4-((3-(4-(4-((5-Chloro-4-((2-(isopropylsulfonyl)phenyl)amino)pyrimidin-2-yl)amino)-5-isopropoxy-2-methylphenyl)piperidin-1-yl)azetidin-1-yl)methyl)piperidin-1-yl)-2-(2,6-dioxopiperidin-3-yl)isoindoline-1,3-dione (**8r**)

Yellow solid. HRMS (ESI) *m*/*z*: calcd, 966.4103 for C_50_H_60_ClN_9_O_7_S [M + H]^+^; found, 966.4098; ^1^H NMR (400 MHz, DMSO-*d*_6_): δ 11.07 (s, 1H), 9.45 (s, 1H), 8.46 (d, *J* = 8.4 Hz, 1H), 8.25 (s, 1H), 8.16 (s, 1H), 8.05 (s, 1H), 7.84 (dd, *J* = 8.0, 1.7 Hz, 1H), 7.74–7.55 (m, 2H), 7.51 (s, 1H), 7.45–7.09 (m, 3H), 6.85 (s, 1H), 5.06 (dd, *J* = 12.9, 5.4 Hz, 1H), 4.59 (p, *J* = 6.1 Hz, 1H), 4.03 (d, *J* = 12.9 Hz, 2H), 3.46–3.35 (m, 2H), 3.09–2.73 (m, 9H), 2.50 (p, *J* = 1.9 Hz, 4H), 2.36 (d, *J* = 6.6 Hz, 3H), 2.11 (s, 3H), 2.05–1.46 (m, 9H), 1.22 (d, *J* = 6.0 Hz, 6H), 1.16 (d, *J* = 6.8 Hz, 6H).

#### 5-(4-(4-((5-Chloro-4-((2-(isopropylsulfonyl)phenyl)amino)pyrimidin-2-yl)amino)-5-isopropoxy-2-methylphenyl)-[1,4′:1′,4″-terpiperidin]-1″-yl)-2-(2,6-dioxopiperidin-3-yl)isoindoline-1,3-dione (**8s**)

Yellow solid. HRMS (ESI) *m*/*z*: calcd, 980.4259 for C_51_H_62_ClN_9_O_7_S [M + H]^+^; found, 980.4250; ^1^H NMR (400 MHz, DMSO-*d*_6_): δ 11.07 (s, 1H), 9.46 (s, 1H), 8.46 (d, *J* = 8.4 Hz, 1H), 8.24 (s, 1H), 8.17 (s, 1H), 8.05 (s, 1H), 7.84 (dd, *J* = 8.0, 1.7 Hz, 1H), 7.69–7.57 (m, 2H), 7.51 (s, 1H), 7.39–7.30 (m, 2H), 7.25 (dd, *J* = 8.7, 2.3 Hz, 1H), 6.83 (s, 1H), 5.06 (dd, *J* = 12.9, 5.4 Hz, 1H), 4.56 (p, *J* = 6.1 Hz, 1H), 4.09 (d, *J* = 12.5 Hz, 2H), 3.45 (d, *J* = 6.8 Hz, 13H), 3.07 (d, *J* = 10.5 Hz, 4H), 2.97 (d, *J* = 11.2 Hz, 6H), 2.89 (s, 1H), 2.63–2.52 (m, 6H), 2.40 (s, 3H), 2.18 (s, 2H), 2.12 (s, 3H), 2.00 (s, 1H), 1.81 (d, *J* = 12.3 Hz, 3H), 1.69 (d, *J* = 8.5 Hz, 4H), 1.48 (d, *J* = 12.3 Hz, 3H), 1.22 (d, *J* = 6.0 Hz, 6H), 1.16 (d, *J* = 6.8 Hz, 6H).

#### 5-(4-((4-(4-((5-Chloro-4-((2-(isopropylsulfonyl)phenyl)amino)pyrimidin-2-yl)amino)-5-isopropoxy-2-methylphenyl)-[1,4′-bipiperidin]-1′-yl)methyl)piperidin-1-yl)-2-(2,6-dioxopiperidin-3-yl)isoindoline-1,3-dione (**8t**)

Yellow solid. HRMS (ESI) *m*/*z*: calcd, 994.4416 for C_52_H_64_ClN_9_O_7_S [M + H]^+^; found, 994.4402; ^1^H NMR (400 MHz, DMSO-*d*_6_): δ 11.07 (s, 1H), 9.46 (s, 1H), 8.46 (d, *J* = 8.4 Hz, 1H), 8.25 (s, 1H), 8.16 (s, 1H), 8.05 (s, 1H), 7.84 (d, *J* = 8.0 Hz, 1H), 7.69–7.58 (m, 3H), 7.51 (s, 1H), 7.40–7.28 (m, 3H), 7.28–7.19 (m, 2H), 6.83 (s, 1H), 5.06 (dd, *J* = 12.9, 5.4 Hz, 2H), 4.56 (p, *J* = 6.0 Hz, 1H), 4.04 (d, *J* = 12.9 Hz, 2H), 3.97 (d, *J* = 13.2 Hz, 1H), 3.10 (d, *J* = 11.1 Hz, 7H), 3.02–2.91 (m, 7H), 2.90–2.81 (m, 3H), 2.63–2.51 (m, 9H), 2.16 (d, *J* = 6.6 Hz, 2H), 2.12 (s, 3H), 2.06–1.97 (m, 2H), 1.90 (d, *J* = 12.1 Hz, 4H), 1.79 (d, *J* = 11.7 Hz, 5H), 1.71 (s, 3H), 1.56–1.69 (m, 4H), 1.22 (d, *J* = 6.0 Hz, 6H), 1.16 (d, *J* = 6.8 Hz, 6H).

### Synthesis of Intermediate **18**

#### ((2-((5-Chloro-2-((2-methoxy-4-(4-(piperazin-1-yl)piperidin-1-yl)phenyl)amino)pyrimidin-4-yl)amino)phenyl)dimethylphosphine oxide)

##### *tert*-Butyl 4-(1-(3-Methoxy-4-nitrophenyl)piperidin-4-yl)piperazine-1-carboxylate (**15**)

To a solution of *tert*-butyl 4-(4-piperidyl) piperazine-1-carboxylate (**14**) (4.0 g, 14.9 mmol) and 4-fluoro-2-methoxy-1-nitro-benzene (2.5 g, 14.9 mmol) in CH_3_CN (60 mL) was added K_2_CO_3_ (4.1 g, 29.7 mmol) at 25 °C under a N_2_ atmosphere. Then, the mixture was stirred at 90 °C for 16 h. TLC (PE/EtOAc = 3:1) showed that all the starting material was consumed and one new main spot (*R*_f_ = 0.5) was detected. The white suspension was poured into water (250 mL) to give a colorless solution. The solution was extracted with EA (150 mL × 3). The organic layer was combined, dried over Na_2_SO_4_, and concentrated under reduced pressure to give the residue. The residue was triturated with EtOAc at 25 °C for 1 h. Then, the solids were collected by filtration and dried under high vacuum to give *tert*-butyl 4-[1-(3-methoxy-4-nitro-phenyl)-4-piperidyl]piperazine-1-carboxylate (**15**) (5.2 g, 83% yield) as a light-yellow solid. LC–MS (ESI+): 421 [M + H]^+^.

##### *tert*-Butyl 4-(1-(4-Amino-3-methoxyphenyl)piperidin-4-yl)piperazine-1-carboxylate (**16**)

To a solution of *tert*-butyl 4-[1-(3-methoxy-4-nitro-phenyl)-4-piperidyl]piperazine-1-carboxylate (**15**) (5.2 g, 12.4 mmol) in THF (50 mL) and EtOH (50 mL) was added Pd/C (1.3 g, 1.2 mmol, 10 wt %) at 25 °C. Then, the black suspension was stirred at 50 °C for 16 h under H_2_ (50 psi). TLC (PE/EA = 3:1) showed that all the starting material was consumed and one new main spot (*R*_f_ = 0.2) was detected. The suspension was filtered through a pad of Celite and the cake was washed with DCM (400 mL). Then, the filtrate was concentrated under reduced pressure to give *tert*-butyl 4-[1-(4-amino-3-methoxy-phenyl)-4-piperidyl] piperazine-1-carboxylate (16) (4.8 g, 11.1 mmol, 89% yield) as a brown gum. LC–MS (ESI+): 391 [M + H]^+^.

##### *tert*-Butyl 4-(1-(4-((5-Chloro-4-((2-(dimethylphosphoryl)phenyl)amino)pyrimidin-2-yl)amino)-3-methoxyphenyl)piperidin-4-yl)piperazine-1-carboxylate (**17**)

A mixture of *tert*-butyl 4-[1-(4-amino-3-methoxy-phenyl)-4-piperidyl] piperazine-1-carboxylate (**16**) (3.9 g, 10.0 mmol), (2-((2,5-dichloropyrimidin-4-yl)amino)phenyl)dimethylphosphine oxide (3.2 g, 10.0 mmol), Pd_2_(dba)_3_ (0.23 g, 0.25 mmol), Xantphos (0.29 g, 0.50 mmol), and Cs_2_CO_3_ (6.5 g, 20 mmol) in 50 mL of 1,4-dioxane was stirred and heated to 100 °C in an oil bath under Argon for 16 h. After cooling to room temperature, the mixture was filtered through Celite. The filtrate was evaporated to dryness. The residue was subjected to silica gel column chromatography eluent with *n*-hexane/EA = 5:1 to 0:100 and then EA/MeOH = 80:20. 3.8 g (yield, 57%) of **17** was obtained as a brown foam. LC–MS (ESI+): 670 [M + H]^+^.

##### (2-((5-Chloro-2-((2-methoxy-4-(4-(piperazin-1-yl)piperidin-1-yl)phenyl)amino)pyrimidin-4-yl)amino)phenyl)dimethylphosphine Oxide (**18**)

A solution of *tert*-butyl 4-(1-(4-((5-chloro-4-((2-(dimethylphosphoryl)phenyl)amino)pyrimidin-2-yl)amino)-3-methoxyphenyl)piperidin-4-yl)piperazine-1-carboxylate (3.0 g, 4.5 mmol) in DCM (18 mL) was added TFA (9.2 g, 80.8 mmol, 6 mL) at 25 °C. Then, it was stirred at 25 °C for 2 h. The mixture was concentrated under vacuum to give the residue. The residue was dissolved in MeOH (20 mL) and adjusted pH to 7–8 with anionic resin, filtered, and concentrated under vacuum to give product 5-chloro-*N*4-(2-dimethylphosphorylphenyl)-*N*2-[2-methoxy-4-(4-piperazin-1-yl-1-piperidyl)phenyl]pyrimidine-2,4-diamine (**18**) (3.1 g, 97% yield) as a brown solid. LC–MS (ESI+): 670 [M + H]^+^.

### Synthesis of Intermediate **23**

#### ((2-((5-Chloro-2-((2-methoxy-4-(piperazin-1-yl)phenyl)amino)pyrimidin-4-yl)amino)phenyl)dimethylphosphine oxide)

##### *tert*-Butyl 4-(3-Methoxy-4-nitrophenyl)piperazine-1-carboxylate (**20**)

To a solution of *tert*-butyl piperazine-1-carboxylate (2.7 g, 14.6 mmol) and 4-fluoro-2-methoxy-1-nitro-benzene (**19**) (2.5 g, 14.6 mmol) in DMSO (30 mL) was added K_2_CO_3_ (4.0 g, 29.2 mmol) at 25 °C under an Argon atmosphere. Then, the mixture was stirred at 80 °C for 16 h. The white suspension was poured into water (100 mL) to give a colorless solution. The solution was extracted with EA (100 mL × 3). The organic layers were combined, dried over Na_2_SO_4_, filtered, and concentrated under reduced pressure to give the crude product. The crude product was triturated, filtrated, and dried under high vacuum to give *tert*-butyl 4-(3-methoxy-4-nitro-phenyl)piperazine-1-carboxylate (**20**) (3.9 g, 71.2% yield) as a light yellow solid. ^1^H NMR (400 MHz, DMSO-*d*_6_): δ 7.89 (d, *J* = 9.2 Hz, 1H), 6.57 (dd, *J* = 2.4, 9.6 Hz, 1H), 6.52 (d, *J* = 2.0 Hz, 1H), 3.91 (s, 3H), 3.47 (s, 8H), 1.43 (s, 9H)

##### *tert*-Butyl 4-(4-Amino-3-methoxyphenyl)piperazine-1-carboxylate (**21**)

To a solution of *tert*-butyl 4-(3-methoxy-4-nitro-phenyl)piperazine-1-carboxylate (**20**) (2.0 g, 5.9 mmol) in MeOH (30 mL) was added Pd/C (630 mg, 0.59 mmol, 10 wt %) at 25 °C. The black suspension was stirred under H_2_ (50 psi) at 25 °C for 16 h. After completion, the suspension was filtered through a pad of Celite, and the cake was washed with DCM (100 mL) and MeOH (20 mL). The filter liquor was concentrated under reduced pressure to give *tert*-butyl 4-(4-amino-3-methoxy-phenyl)piperazine-1-carboxylate (**21**) (2.0 g, 98% yield) as a brown gum. LC–MS (ESI+): 308 [M + H]^+^. ^1^H NMR (400 MHz, CDCl_3_): δ 6.67 (d, *J* = 8.4 Hz, 1H), 6.53 (d, *J* = 2.5 Hz, 1H), 6.43 (dd, *J* = 8.4, 2.5 Hz, 1H), 3.86 (s, 3H), 3.71–3.45 (m, 4H), 3.13–2.76 (m, 4H), 1.50 (s, 9H)

##### *tert*-Butyl 4-(4-((5-Chloro-4-((2-(dimethylphosphoryl)phenyl)amino)pyrimidin-2-yl)amino)-3-methoxyphenyl)piperazine-1-carboxylate (**22**)

A mixture of *tert*-butyl 4-(4-amino-3-methoxy-phenyl)piperazine-1-carboxylate (**21**) (2.6 g, 8.4 mmol), (2-((2,5-dichloropyrimidin-4-yl)amino)phenyl)dimethylphosphine oxide (2.7 g, 8.4 mmol), Pd_2_(dba)_3_ (0.20 g, 0.21 mmol), Xantphos (0.29 g, 0.42 mmol), and Cs_2_CO_3_ (5.5 g, 16.8 mmol) in 100 mL of 1,4-dioxane was stirred and heated to 100 °C in an oil bath under Argon for 16 h. After cooling to room temperature, the mixture was filtered through Celite. The filtrate was evaporated to dryness. The residue was subjected to silica gel column chromatography eluent with *n*-hexane/EA = 5:1 to 0:100 and then EA/MeOH = 80:20. 1.8 g (yield, 38%) of **22** was obtained as a brown foam. LC–MS (ESI+): 587 [M + H]^+^. ^1^H NMR (400 MHz, CDCl_3_): δ 10.83 (s, 1H), 8.64 (d, *J* = 8.5 Hz, 1H), 8.19–8.09 (m, 2H), 7.51 (d, *J* = 8.6 Hz, 1H), 7.26–7.29 (m, 2H), 7.15 (t, *J* = 7.5 Hz, 1H), 6.57 (d, *J* = 2.5 Hz, 1H), 6.50 (d, *J* = 8.8 Hz, 1H), 3.89 (s, 3H), 3.66–3.59 (m, 4H), 3.10 (t, *J* = 5.1 Hz, 4H), 1.85 (d, *J* = 13.1 Hz, 6H), 1.51 (s, 9H).

##### (2-((5-Chloro-2-((2-methoxy-4-(piperazin-1-yl)phenyl)amino)pyrimidin-4-yl)amino)phenyl)dimethylphosphine Oxide (**23**)

To a solution of *tert*-butyl 4-[4-[[5-chloro-4-(2-dimethyl phosphorylanilino)pyrimidin-2-yl] amino]-3-methoxy-phenyl]piperazine-1-carboxylate (**22**) (600 mg, 1.02 mmol) in DCM (6 mL) was added TFA (3.1 g, 27 mmol, 2 mL) at 25 °C. The mixture was stirred at 25 °C for 2 h. After completion, the mixture was concentrated under vacuum to give the residue. The residue was dissolved in MeOH (20 mL), adjusted to pH = 7–8 with anionic resin, filtered, and concentrated under vacuum to provide 5-chloro-*N*4-(2-dimethyl phosphorylphenyl)-*N*2-(2-methoxy-4-piperazin-1-yl-phenyl)pyrimidine-2,4-diamine (**23**) (540 mg, 87% yield) as a brown gum.

### Synthesis of **9a−9v**

#### 5-(4-((4-(4-((5-Chloro-4-((2-(dimethylphosphoryl)phenyl)amino)pyrimidin-2-yl)amino)-3-methoxyphenyl)-[1,4′-bipiperidin]-1′-yl)methyl)piperidin-1-yl)-2-(2,6-dioxopiperidin-3-yl)isoindoline-1,3-dione (**9g**)

##### *tert*-Butyl 4-(3-Methoxy-4-nitrophenyl)-3,6-dihydropyridine-1(2*H*)-carboxylate (**25**)

A mixture of 4-bromo-2-methoxy-1-nitrobenzene (**24**) (2.3 g, 10 mmol), *tert*-butyl 4-(4,4,5,5-tetramethyl-1,3,2-dioxaborolan-2-yl)-3,6-dihydropyridine-1(2*H*)-carboxylate (3.7 g, 12 mmol), Pd(dppf)Cl_2_ (0.35 g, 0.50 mmol), and potassium carbonate (2.8 g, 20 mmol) in 1,4-dioxane/water (50 mL/50 mL) was stirred at 100 °C for 3 h under an Argon atmosphere. After cooling to room temperature, the mixture was extracted with EA (3 × 50 mL). The combined extracts were dried over Na_2_SO_4_, filtered, and evaporated to dryness to provide **25** (3.0 g, 89%) as a yellow solid. ^1^H NMR (400 MHz, CDCl_3_): δ 7.89 (d, *J* = 8.4 Hz, 1H), 7.03 (d, *J* = 8.0 Hz, 2H), 6.19 (s, 1H), 4.21–4.08 (m, 2H), 4.00 (s, 3H), 3.68 (t, *J* = 5.6 Hz, 2H), 2.54 (s, 2H), 1.52 (s, 9H)

##### *tert*-Butyl 4-(4-(3-Methoxy-4-nitrophenyl)-3,6-dihydropyridin-1(2*H*)-yl)piperidine-1-carboxylate (**27**)

Following general procedure B, 4-(3-methoxy-4-nitrophenyl)-1,2,3,6-tetrahydropyridine (**26**) was obtained and used in the next step without further purification. Following general procedure A, with *tert*-butyl 4-oxopiperidine-1-carboxylate (3.0 g, 15 mmol), *tert*-butyl 4-(4-(3-methoxy-4-nitrophenyl)-3,6-dihydropyridin-1(2*H*)-yl)piperidine-1-carboxylate (**27**) (3.6 g, 96% in two steps) was obtained as a yellow oil. LC–MS (ESI+): 418 [M + H]^+^. ^1^H NMR (400 MHz, CDCl_3_): δ 7.87 (d, *J* = 8.3 Hz, 1H), 7.12–6.97 (m, 2H), 6.24 (t, *J* = 3.7, 1H), 4.18 (d, *J* = 14.6 Hz, 2H), 3.98 (s, 3H), 3.36 (q, *J* = 3.0 Hz, 2H), 2.83 (t, *J* = 5.6 Hz, 2H), 2.79–2.66 (m, 2H), 2.64–2.53 (m, 3H), 1.89 (d, *J* = 12.5 Hz, 2H), 1.66 (d, *J* = 20.0 Hz, 1H), 1.48 (s, 9H).

##### *tert*-Butyl 4-(4-Amino-3-methoxyphenyl)-[1,4′-bipiperidine]-1′-carboxylate (**28**)

A suspension of the above-obtained (**27**) and 0.5 g of Pd/C in MeOH was stirred at room temperature under a hydrogen atmosphere for 2 h. After completion, the mixture was filtered through Celite. The filtrate was evaporated in vacuo to provide (**28**) (3.3 g, quant.) as a brown oil. LC–MS (ESI+): 390 [M + H]^+^.

##### *tert*-Butyl 4-(4-((5-Chloro-4-((2-(dimethylphosphoryl)phenyl)amino)pyrimidin-2-yl)amino)-3-methoxyphenyl)-[1,4′-bipiperidine]-1′-carboxylate (**29**)

Following general procedure C, by using 2.0 g of (**28**), the title compound (**29**) (2.0 g, 58%) was obtained as a brown oil. LC–MS (ESI+): 669 [M + H]^+^. ^1^H NMR (400 MHz, CDCl_3_): δ 10.82 (s, 1H), 8.66–8.58 (m, 1H), 8.24 (d, *J* = 8.5 Hz, 1H), 8.12 (s, 1H), 7.52 (t, *J* = 7.3 Hz, 1H), 7.47 (s, 1H), 7.29–7.32 (m, 1H), 7.16 (t, *J* = 7.5 Hz, 1H), 6.80 (d, *J* = 7.5 Hz, 2H), 4.20 (s, 1H), 3.89 (s, 3H), 3.74 (q, *J* = 7.0 Hz, 1H), 3.51 (s, 3H), 3.08 (d, *J* = 10.9 Hz, 2H), 2.73 (s, 1H), 2.47 (d, *J* = 11.5 Hz, 2H), 2.31 (d, *J* = 11.5 Hz, 2H), 1.86 (s, 6H), 1.57–1.49 (m, 3H), 1.48 (s, 9H), 1.35–1.20 (m, 2H).

Following general procedure B, (2-((2-((4-([1,4′-bipiperidin]-4-yl)-2-methoxyphenyl)amino)-5-chloropyrimidin-4-yl)amino)phenyl)dimethylphosphine oxide (**30**) was obtained as a white solid. LC–MS (ESI+): 569 [M + H]^+^. Following general procedure A, (**30**) (110 mg, 0.2 mmol) was used. The mixture was subjected to silica gel eluent with *n*-hexane/EA = 5:1 to 0:100 and then EA/MeOH = 8:2. The crude product was further purified by C18 reverse phase preparative HPLC to provide 5-(4-((4-(4-((5-chloro-4-((2-(dimethylphosphoryl)phenyl)amino)pyrimidin-2-yl)amino)-3-methoxyphenyl)-[1,4′-bipiperidin]-1′-yl)methyl)piperidin-1-yl)-2-(2,6-dioxopiperidin-3-yl)isoindoline-1,3-dione (**9g**) (54 mg, 30%) as a yellow solid. HRMS (ESI) *m*/*z*: calcd, 922.3936 for C_48_H_57_ClN_9_O_6_P [M + H]^+^; found, 922.3928. ^1^H NMR (400 MHz, DMSO-*d*_6_): δ 11.16 (s, 1H), 11.07 (s, 1H), 8.46 (d, *J* = 8.4 Hz, 1H), 8.20 (s, 2H), 8.11 (d, *J* = 12.8 Hz, 2H), 7.65 (d, *J* = 8.3 Hz, 2H), 7.56 (d, *J* = 13.9 Hz, 1H), 7.40 (t, *J* = 7.9 Hz, 1H), 7.30 (d, *J* = 2.3 Hz, 1H), 7.22 (d, *J* = 8.7 Hz, 1H), 7.14 (t, *J* = 7.7 Hz, 1H), 6.92 (d, *J* = 1.9 Hz, 1H), 6.77 (d, *J* = 8.2 Hz, 1H), 5.06 (d, *J* = 12.9 Hz, 1H), 4.03 (d, *J* = 12.9 Hz, 2H), 3.80 (s, 4H), 3.08 (d, *J* = 10.6 Hz, 3H), 3.01–2.81 (m, 7H), 2.63–2.52 (m, 4H), 2.51 (d, *J* = 1.8 Hz, 5H), 2.43–2.33 (m, 3H), 2.15 (d, *J* = 6.6 Hz, 2H), 2.07–1.97 (m, 2H), 1.90 (t, *J* = 11.2 Hz, 3H), 1.78 (d, *J* = 13.3 Hz, 17H), 1.58–1.45 (m, 2H), 1.14 (d, *J* = 12.2 Hz, 2H).

#### 5-(4-(4-((5-Chloro-4-((2-(dimethylphosphoryl)phenyl)amino)pyrimidin-2-yl)amino)-3-methoxyphenyl)piperazin-1-yl)-2-(2,6-dioxopiperidin-3-yl)isoindoline-1,3-dione (**9a**)

General procedure D was followed with **23**. Yellow solid. HRMS (ESI) *m*/*z*: calcd, 729.2469 for C_36_H_38_ClN_8_O_5_P [M + H]^+^; found, 729.2469. ^1^H NMR (400 MHz, DMSO-*d*_6_): δ 11.11 (s, 1H), 8.41 (s, 1H), 8.08 (s, 1H), 7.73 (d, *J* = 8.5 Hz, 1H), 7.61–7.25 (m, 2H), 7.09 (t, *J* = 7.3 Hz, 1H), 6.72 (d, *J* = 2.5 Hz, 1H), 6.54 (d, *J* = 8.61H), 5.08 (dd, *J* = 12.9, 5.5 Hz, 1H), 3.79 (s, 4H), 2.87 (d, *J* = 17.4 Hz, 2H), 2.03 (d, *J* = 11.0 Hz, 1H), 1.76 (d, *J* = 13.5 Hz, 6H).

#### 5-(3-((4-(4-((5-Chloro-4-((2-(dimethylphosphoryl)phenyl)amino)pyrimidin-2-yl)amino)-3-methoxyphenyl)piperazin-1-yl)methyl)azetidin-1-yl)-2-(2,6-dioxopiperidin-3-yl)isoindoline-1,3-dione (**9b**)

General procedures D and A were followed. Yellow solid. HRMS (ESI) *m*/*z*: calcd, 812.2840 for C_40_H_43_ClN_9_O_6_P [M + H]^+^; found, 812.2819. ^1^H NMR (400 MHz, DMSO-*d*_6_): δ 11.15 (s, 1H), 11.07 (s, 1H), 8.50 (s, 1H), 8.07 (s, 2H), 7.65 (d, *J* = 8.4 Hz, 1H), 7.54–7.49 (m, 1H), 7.41 (d, *J* = 8.8 Hz, 1H), 7.36–7.30 (m, 1H), 7.15–7.08 (m, 1H), 6.79 (d, *J* = 2.0 Hz, 1H), 6.69–6.63 (m, 2H), 6.48 (dd, *J* = 2.4, 8.8 Hz, 1H), 5.06 (dd, *J* = 5.2, 12.8 Hz, 1H), 4.17 (t, *J* = 8.4 Hz, 2H), 3.77 (s, 3H), 3.75–3.69 (m, 2H), 3.16 (s, 4H), 3.07–2.87 (m, 2H), 2.68 (m, 2H), 2.57–2.50 (m, 6H), 2.06–1.97 (m, 1H), 1.79 (s, 3H), 1.75 (s, 3H).

#### 5-(4-((4-(4-((5-Chloro-4-((2-(dimethylphosphoryl)phenyl)amino)pyrimidin-2-yl)amino)-3-methoxyphenyl)piperazin-1-yl)methyl)piperidin-1-yl)-2-(2,6-dioxopiperidin-3-yl)isoindoline-1,3-dione (**9c**)

General procedures D and A were followed. Yellow solid. HRMS (ESI) *m*/*z*: calcd, 840.3153 for C_42_H_47_ClN_9_O_6_P [M + H]^+^; found, 840.3152. ^1^H NMR (400 MHz, DMSO-*d*_6_): δ 11.15 (s, 1H), 11.08 (s, 1H), 8.52 (s, 1H), 8.07 (s, 2H), 7.66 (d, *J* = 8.8 Hz, 1H), 7.54 (m, 1H), 7.41 (d, *J* = 8.4 Hz, 1H), 7.38–7.30 (m, 2H), 7.24 (dd, *J* = 2.0, 8.8 Hz, 1H), 7.11–7.05 (m, 1H), 6.64 (d, *J* = 2.0 Hz, 1H), 6.48 (dd, *J* = 2.4, 8.8 Hz, 1H), 5.07 (dd, *J* = 5.2, 12.8 Hz, 1H), 4.11–4.02 (m, 2H), 3.77 (s, 3H), 3.19–3.13 (m, 4H), 3.05–2.82 (m, 4H), 2.68–2.55 (m, 4H), 2.22 (d, *J* = 6.8 Hz, 2H), 2.07–1.98 (m, 1H), 1.91–1.80 (m, 4H), 1.79 (s, 3H), 1.75 (s, 3H), 1.26–1.11 (m, 2H).

#### 5-(3-(4-(1-(4-((5-Chloro-4-((2-(dimethylphosphoryl)phenyl)amino)pyrimidin-2-yl)amino)-3-methoxyphenyl)piperidin-4-yl)piperazin-1-yl)azetidin-1-yl)-2-(2,6-dioxopiperidin-3-yl)isoindoline-1,3-dione (**9d**)

Yellow solid. HRMS (ESI) *m*/*z*: calcd, 881.3419 for C_44_H_50_ClN_10_O_6_P [M + H]^+^; found, 881.3409. ^1^H NMR (400 MHz, DMSO-*d*_6_): δ 11.17 (s, 1H), 11.07 (s, 1H), 8.49 (s, 1H), 8.07 (d, *J* = 2.3 Hz, 2H), 7.66 (d, *J* = 8.3 Hz, 1H), 7.53 (d, *J* = 14.0 Hz, 1H), 7.44–7.29 (m, 3H), 7.14–7.05 (m, 1H), 6.80 (d, *J* = 2.1 Hz, 1H), 6.70–6.62 (m, 2H), 6.48 (d, *J* = 8.8 Hz, 1H), 5.06 (dd, *J* = 12.9, 5.4 Hz, 1H), 4.15–4.07 (m, 2H), 3.87 (dd, *J* = 8.9, 4.8 Hz, 2H), 3.82–3.77 (m, 2H), 3.77 (s, 3H), 2.88 (d, *J* = 17.5 Hz, 4H), 2.68 (t, *J* = 11.8 Hz, 4H), 2.51 (s, 6H), 2.06–1.92 (m, 3H), 1.77 (d, *J* = 13.5 Hz, 6H), 1.64–1.56 (m, 2H).

#### 5-(3-((4-(1-(4-((5-Chloro-4-((2-(dimethylphosphoryl)phenyl)amino)pyrimidin-2-yl)amino)-3-methoxyphenyl)piperidin-4-yl)piperazin-1-yl)methyl)azetidin-1-yl)-2-(2,6-dioxopiperidin-3-yl)isoindoline-1,3-dione (**9e**)

Yellow solid. HRMS (ESI) *m*/*z*: calcd, 895.3575 for C_45_H_52_ClN_10_O_6_P [M + H]^+^; found, 895.3560. ^1^H NMR (400 MHz, DMSO-*d*_6_): δ 11.17 (s, 1H), 11.07 (s, 1H), 8.49 (s, 1H), 8.15 (s, 1H), 8.06 (d, *J* = 5.0 Hz, 2H), 7.63 (d, *J* = 8.2 Hz, 1H), 7.53 (d, *J* = 14.0 Hz, 1H), 7.36 (d, *J* = 22.1 Hz, 2H), 7.10 (t, *J* = 7.5 Hz, 1H), 6.77 (d, *J* = 2.1 Hz, 1H), 6.64 (d, *J* = 8.2 Hz, 2H), 6.47 (d, *J* = 8.7 Hz, 1H), 5.05 (dd, *J* = 12.8, 5.4 Hz, 1H), 4.13 (t, *J* = 8.1 Hz, 2H), 3.78–3.64 (m, 9H), 3.03–2.95 (m, 2H), 2.88 (d, *J* = 17.5 Hz, 2H), 2.72–2.52 (m, 11H), 2.44 (s, 2H), 2.06–1.96 (m, 1H), 1.87 (d, *J* = 12.1 Hz, 2H), 1.77 (d, *J* = 13.5 Hz, 6H), 1.55 (td, *J* = 12.0, 3.8 Hz, 2H).

#### 5-(4-((4-(1-(4-((5-Chloro-4-((2-(dimethylphosphoryl)phenyl)amino)pyrimidin-2-yl)amino)-3-methoxyphenyl)piperidin-4-yl)piperazin-1-yl)methyl)piperidin-1-yl)-2-(2,6-dioxopiperidin-3-yl)isoindoline-1,3-dione (**9f**)

Yellow solid. HRMS (ESI) *m*/*z*: calcd, 923.3888 for C_47_H_56_ClN_10_O_6_P [M + H]^+^; found, 923.3875. ^1^H NMR (400 MHz, DMSO-*d*_6_): δ 11.17 (s, 1H), 11.07 (s, 1H), 8.48 (s, 1H), 8.16 (s, 1H), 8.06 (d, *J* = 6.4 Hz, 2H), 7.65 (d, *J* = 8.6 Hz, 1H), 7.53 (d, *J* = 14.1 Hz, 1H), 7.42–7.28 (m, 3H), 7.22 (d, *J* = 8.8 Hz, 1H), 7.14–7.05 (m, 1H), 6.63 (d, *J* = 2.6 Hz, 1H), 6.47 (d, *J* = 8.7 Hz, 1H), 5.06 (dd, *J* = 12.9, 5.4 Hz, 1H), 4.03 (d, *J* = 12.9 Hz, 2H), 3.76 (s, 6H), 3.01–2.82 (m, 4H), 2.72–2.58 (m, 4H), 2.56 (q, *J* = 4.4 Hz, 5H), 2.37 (d, *J* = 12.1 Hz, 3H), 2.14 (d, *J* = 6.7 Hz, 2H), 2.02 (d, *J* = 7.5 Hz, 1H), 1.87 (d, *J* = 11.9 Hz, 2H), 1.77 (d, *J* = 13.6 Hz, 7H), 1.54 (t, *J* = 12.9, 2H), 1.21–1.08 (m, 2H).

#### 5-(4-((4-(4-(4-((5-Chloro-4-((2-(dimethylphosphoryl)phenyl)amino)pyrimidin-2-yl)amino)-3-methoxyphenyl)piperazin-1-yl)piperidin-1-yl)methyl)piperidin-1-yl)-2-(2,6-dioxopiperidin-3-yl)isoindoline-1,3-dione (**9h**)

Yellow solid. HRMS (ESI) *m*/*z*: calcd, 923.3888 for C_47_H_56_ClN_10_O_6_P [M + H]^+^; found, 923.3885. ^1^H NMR (400 MHz, DMSO-*d*_6_): δ 11.16 (s, 1H), 11.08 (s, 1H), 8.48 (s, 1H), 8.20 (s, 1H), 8.06 (s, 2H), 7.65 (d, *J* = 8.4 Hz, 1H), 7.53 (d, *J* = 14.1 Hz, 1H), 7.40 (d, *J* = 8.5 Hz, 1H), 7.36–7.28 (m, 2H), 7.22 (d, *J* = 8.8 Hz, 1H), 7.10 (t, *J* = 7.6 Hz, 1H), 6.62 (d, *J* = 2.5 Hz, 1H), 6.46 (d, *J* = 8.7 Hz, 1H), 5.06 (dd, *J* = 12.9, 5.5 Hz, 1H), 4.03 (d, *J* = 12.9 Hz, 3H), 3.76 (s, 9H), 3.72 (s, 7H), 3.13 (s, 4H), 2.97 (d, *J* = 13.6 Hz, 2H), 2.94–2.81 (m, 4H), 2.69–2.59 (m, 5H), 2.55 (dd, *J* = 9.2, 3.3 Hz, 2H), 2.26 (d, *J* = 11.7 Hz, 1H), 2.18 (d, *J* = 6.6 Hz, 2H), 2.05–1.88 (m, 4H), 1.78 (t, *J* = 12.3 Hz, 6H), 1.47 (d, *J* = 12.6 Hz, 2H), 1.15 (s, 1H), 1.14–1.09 (m, 1H).

#### 4-(3-((4-(1-(4-((5-Chloro-4-((2-(dimethylphosphoryl)phenyl)amino)pyrimidin-2-yl)amino)-3-methoxyphenyl)piperidin-4-yl)piperazin-1-yl)methyl)azetidin-1-yl)-2-(2,6-dioxopiperidin-3-yl)isoindoline-1,3-dione (**9i**)

Yellow solid. HRMS (ESI) *m*/*z*: calcd, 895.3575 for C_45_H_52_ClN_10_O_6_P [M + H]^+^; found, 895.3572. ^1^H NMR (400 MHz, DMSO-*d*_6_): δ 11.17 (s, 1H), 11.06 (s, 1H), 8.48 (s, 1H), 8.15 (s, 1H), 8.06 (d, *J* = 7.8 Hz, 2H), 7.60–7.48 (m, 2H), 7.36 (d, *J* = 22.5 Hz, 2H), 7.10 (d, *J* = 7.7, 2H), 6.78 (d, *J* = 8.6 Hz, 1H), 6.63 (d, *J* = 2.5 Hz, 1H), 6.47 (dd, *J* = 8.8, 2.5 Hz, 1H), 5.04 (dd, *J* = 12.8, 5.4 Hz, 1H), 4.29 (s, 1H), 3.83 (t, *J* = 7.5 Hz, 2H), 3.76 (s, 6H), 2.87–2.91 (m, 3H), 2.67 (t, *J* = 11.8 Hz, 3H), 2.51 (s, 9H), 2.42 (s, 2H), 2.05–1.96 (m, 1H), 1.87 (d, *J* = 12.2 Hz, 2H), 1.77 (d, *J* = 13.6 Hz, 6H), 1.54 (q, *J* = 11.0 Hz, 2H).

#### 4-(3-(4-(1-(4-((5-Chloro-4-((2-(dimethylphosphoryl)phenyl)amino)pyrimidin-2-yl)amino)-3-methoxyphenyl)piperidin-4-yl)piperazin-1-yl)azetidin-1-yl)-2-(2,6-dioxopiperidin-3-yl)isoindoline-1,3-dione (**9j**)

Yellow solid. HRMS (ESI) *m*/*z*: calcd, 881.3419 for C_44_H_50_ClN_10_O_6_P [M + H]^+^; found, 881.3424. ^1^H NMR (400 MHz, DMSO-*d*_6_): δ 11.16 (s, 1H), 11.07 (s, 1H), 8.48 (s, 1H), 8.25–7.92 (m, 2H), 7.68–7.45 (m, 2H), 7.44 (s, 2H), 7.26–6.97 (m, 2H), 6.80 (d, *J* = 8.5 Hz, 1H), 6.63 (d, *J* = 2.5 Hz, 1H), 6.47 (d, *J* = 8.7 Hz, 1H), 5.05 (dd, *J* = 12.7, 5.4 Hz, 1H), 4.27 (s, 2H), 3.96 (d, *J* = 9.4 Hz, 2H), 3.76 (s, 6H), 3.20 (t, *J* = 6.3 Hz, 3H), 2.87 (d, *J* = 17.0 Hz, 2H), 2.75–2.62 (m, 2H), 2.38 (s, 4H), 2.10–1.90 (m, 1H), 1.87 (d, *J* = 12.0 Hz, 2H), 1.76 (d, *J* = 13.5 Hz, 6H), 1.54 (q, *J* = 12.0 Hz, 2H).

#### 4-(4-((4-(1-(4-((5-Chloro-4-((2-(dimethylphosphoryl)phenyl)amino)pyrimidin-2-yl)amino)-3-methoxyphenyl)piperidin-4-yl)piperazin-1-yl)methyl)piperidin-1-yl)-2-(2,6-dioxopiperidin-3-yl)isoindoline-1,3-dione (**9k**)

Yellow solid. HRMS (ESI) *m*/*z*: calcd, 923.3888 for C_47_H_56_ClN_10_O_6_P [M + H]^+^; found, 923.3869. ^1^H NMR (400 MHz, DMSO-*d*_6_): δ 11.17 (s, 1H), 11.08 (s, 1H), 8.48 (s, 1H), 8.16 (s, 1H), 8.06 (d, *J* = 7.9 Hz, 2H), 7.67 (d, *J* = 8.5 Hz, 1H), 7.53 (d, *J* = 14.0 Hz, 1H), 7.38 (t, *J* = 7.6 Hz, 1H), 7.32 (d, *J* = 7.9 Hz, 3H), 7.14–7.05 (m, 1H), 6.63 (d, *J* = 2.5 Hz, 1H), 6.47 (dd, *J* = 8.8, 2.5 Hz, 1H), 5.08 (dd, *J* = 12.8, 5.4 Hz, 1H), 3.74 (d, *J* = 21.6 Hz, 9H), 2.89–2.93 (m, 4H), 2.72–2.60 (m, 4H), 2.60–2.55 (m, 3H), 2.41 (s, 2H), 2.20 (d, *J* = 7.0 Hz, 2H), 2.07–1.98 (m, 1H), 1.88 (d, *J* = 12.0 Hz, 2H), 1.77 (d, *J* = 13.5 Hz, 6H), 1.61–1.48 (m, 2H), 1.31 (d, *J* = 11.9 Hz, 2H).

#### 3-(5-(4-(4-((5-Chloro-4-((2-(dimethylphosphoryl)phenyl)amino)pyrimidin-2-yl)amino)-3-methoxyphenyl)piperazin-1-yl)-1-oxoisoindolin-2-yl)piperidine-2,6-dione (**9l**)

White solid. HRMS (ESI) *m*/*z*: calcd, 729.2469 for C_36_H_38_ClN_8_O_5_P [M + H]^+^; found, 729.2469. ^1^H NMR (400 MHz, DMSO-*d*_6_): δ 11.14 (s, 1H), 10.94 (s, 1H), 8.47 (s, 1H), 8.07 (s, 1H), 7.51 (m, 1H), 7.41–6.94 (m, 2H), 6.72 (s, 1H), 6.55 (d, *J* = 8.8 Hz, 1H), 5.07 (t, *J* = 14.0 Hz, 1H), 4.46–4.16 (m, 2H), 3.79 (s, 3H), 2.90 (s, 1H), 1.99 (s, 1H), 1.76 (d, *J* = 13.5 Hz, 6H), 1.23 (m, 1H), 0.84 (m, 1H).

#### 3-(5-(3-((4-(4-((5-Chloro-4-((2-(dimethylphosphoryl)phenyl)amino)pyrimidin-2-yl)amino)-3-methoxyphenyl)piperazin-1-yl)methyl)azetidin-1-yl)-1-oxoisoindolin-2-yl)piperidine-2,6-dione (**9m**)

White solid. HRMS (ESI) *m*/*z*: calcd, 798.3048 for C_40_H_45_ClN_9_O_5_P [M + H]^+^; found, 798.3047. ^1^H NMR (400 MHz, DMSO-*d*_6_): δ 11.13 (s, 1H), 10.93 (d, *J* = 11.6 Hz, 1H), 8.47 (s, 1H), 8.06 (d, *J* = 6.3 Hz, 2H), 7.58–7.46 (m, 2H), 7.38 (dt, *J* = 23.0, 8.3 Hz, 2H), 7.11 (t, *J* = 7.5 Hz, 1H), 6.64 (d, *J* = 2.6 Hz, 1H), 6.54–6.43 (m, 2H), 5.05 (ddd, *J* = 18.6, 13.2, 5.1 Hz, 1H), 4.31 (dd, *J* = 16.8, 4.8 Hz, 1H), 4.19 (dd, *J* = 16.8, 4.6 Hz, 1H), 4.03 (dt, *J* = 16.4, 7.6 Hz, 2H), 3.76 (s, 3H), 3.63–3.49 (m, 9H), 3.15 (t, *J* = 4.8 Hz, 5H), 3.00 (dd, *J* = 13.1, 6.3 Hz, 3H), 2.90 (ddt, *J* = 17.5, 13.2, 5.9 Hz, 3H), 2.66 (d, *J* = 7.2 Hz, 2H), 2.58 (q, *J* = 8.1 Hz, 6H), 2.33 (s, 3H), 1.94 (dq, *J* = 13.0, 7.1 Hz, 2H), 1.76 (d, *J* = 13.5 Hz, 6H), 1.14 (t, *J* = 7.3 Hz, 1H).

#### 3-(5-(4-((4-(4-((5-Chloro-4-((2-(dimethylphosphoryl)phenyl)amino)pyrimidin-2-yl)amino)-3-methoxyphenyl)piperazin-1-yl)methyl)piperidin-1-yl)-1-oxoisoindolin-2-yl)piperidine-2,6-dione (**9n**)

White solid. HRMS (ESI) *m*/*z*: calcd, 826.3361 for C_42_H_49_ClN_9_O_5_P [M + H]^+^; found, 826.3343. ^1^H NMR (400 MHz, DMSO-*d*_6_): δ 11.13 (s, 1H), 10.92 (s, 1H), 8.47 (s, 1H), 8.05 (d, *J* = 8.2 Hz, 2H), 7.61 (s, 1H), 7.51 (d, *J* = 8.1 Hz, 2H), 7.41 (d, *J* = 8.4 Hz, 1H), 7.35 (s, 1H), 7.10 (s, 1H), 7.05 (d, *J* = 8.2 Hz, 2H), 6.63 (s, 1H), 6.47 (d, *J* = 8.5 Hz, 1H), 4.32 (d, *J* = 16.3 Hz, 3H), 3.86 (s, 3H), 3.76 (s, 6H), 3.01–3.15 (m, 6H), 2.22 (s, 2H), 1.76 (d, *J* = 13.5 Hz, 6H), 1.23 (s, 2H).

#### 3-(5-(4-((4-(1-(4-((5-Chloro-4-((2-(dimethylphosphoryl)phenyl)amino)pyrimidin-2-yl)amino)-3-methoxyphenyl)piperidin-4-yl)piperazin-1-yl)methyl)piperidin-1-yl)-1-oxoisoindolin-2-yl)piperidine-2,6-dione (**9o**)

White solid. HRMS (ESI) *m*/*z*: calcd, 909.4096 for C_47_H_58_ClN_10_O_5_P [M + H]^+^; found, 909.4102. ^1^H NMR (400 MHz, DMSO-*d*_6_): δ 11.15 (s, 1H), 10.91 (s, 1H), 8.49 (d, *J* = 5.1 Hz, 1H), 8.19 (s, 2H), 8.07 (s, 1H), 8.02 (s, 1H), 7.58–7.46 (m, 3H), 7.44–7.31 (m, 3H), 7.11 (d, *J* = 7.2 Hz, 1H), 7.07 (d, *J* = 6.9 Hz, 1H), 7.03 (d, *J* = 7.7 Hz, 2H), 6.63 (d, *J* = 2.6 Hz, 1H), 6.47 (dd, *J* = 8.8, 2.5 Hz, 1H), 5.03 (dd, *J* = 13.2, 5.1 Hz, 1H), 4.32 (d, *J* = 16.8 Hz, 2H), 4.20 (d, *J* = 16.8 Hz, 2H), 4.06 (s, 1H), 3.86 (d, *J* = 12.8 Hz, 5H), 3.76 (s, 12H), 2.97–2.77 (m, 6H), 2.73–2.66 (m, 3H), 2.61 (dd, *J* = 16.4, 12.6 Hz, 5H), 2.56 (s, 3H), 2.40 (s, 3H), 2.36 (dd, *J* = 12.4, 5.5 Hz, 4H), 2.16 (d, *J* = 6.6 Hz, 3H), 2.00–1.92 (m, 2H), 1.87 (d, *J* = 11.9 Hz, 3H), 1.77 (d, *J* = 13.5 Hz, 6H), 1.55 (q, *J* = 11.9 Hz, 3H), 1.22–1.09 (m, 3H).

#### 3-(5-(3-((4-(1-(4-((5-Chloro-4-((2-(dimethylphosphoryl)phenyl)amino)pyrimidin-2-yl)amino)-3-methoxyphenyl)piperidin-4-yl)piperazin-1-yl)methyl)azetidin-1-yl)-1-oxoisoindolin-2-yl)piperidine-2,6-dione (**9p**)

White solid. HRMS (ESI) *m*/*z*: calcd, 881.3782 for C_45_H_54_ClN_10_O_5_P [M + H]^+^; found, 881.3791. ^1^H NMR (400 MHz, DMSO-*d*_6_): δ 11.16 (s, 1H), 10.92 (s, 1H), 8.48 (s, 1H), 8.18 (s, 1H), 8.05 (d, *J* = 11.7 Hz, 2H), 7.58–7.44 (m, 2H), 7.36–7.40 (m, 2H), 7.10 (t, *J* = 7.5 Hz, 1H), 6.63 (d, *J* = 2.5 Hz, 1H), 6.52–6.43 (m, 3H), 5.02 (dd, *J* = 13.3, 5.1 Hz, 1H), 4.29 (d, *J* = 16.8 Hz, 2H), 4.17 (d, *J* = 16.8 Hz, 2H), 4.01 (t, *J* = 7.7 Hz, 4H), 3.75 (d, *J* = 10.6 Hz, 8H), 2.91–2.99 (m, 3H), 2.72–2.56 (m, 8H), 2.55 (s, 3H), 2.42 (s, 3H), 2.39–2.28 (m, 4H), 1.98–1.91 (m, 2H), 1.91–1.83 (m, 3H), 1.76 (d, *J* = 13.4 Hz, 6H), 1.61–1.49 (m, 2H).

#### 3-((4-(4-((4-(1-(4-((5-Chloro-4-((2-(dimethylphosphoryl)phenyl)amino)pyrimidin-2-yl)amino)-3-methoxyphenyl)piperidin-4-yl)piperazin-1-yl)methyl)piperidin-1-yl)phenyl)amino)piperidine-2,6-dione (**9q**)

Brown solid. HRMS (ESI) *m*/*z*: calcd, 869.4147 for C_45_H_58_ClN_10_O_4_P [M + H]^+^; found, 869.4173. ^1^H NMR (400 MHz, DMSO-*d*_6_): δ 11.16 (s, 1H), 10.74 (s, 1H), 8.52–8.46 (m, 1H), 8.14 (s, 2H), 8.06 (d, *J* = 6.7 Hz, 2H), 7.53 (d, *J* = 14.1 Hz, 1H), 7.45–7.32 (m, 2H), 7.10 (t, *J* = 7.1 Hz, 1H), 6.76 (d, *J* = 8.5 Hz, 2H), 6.67–6.57 (m, 3H), 6.50 (d, *J* = 8.7 Hz, 2H), 4.18 (dd, *J* = 11.2, 4.7 Hz, 2H), 3.81 (s, 3H), 3.77 (s, 5H), 2.94 (s, 2H), 2.85 (d, *J* = 11.9 Hz, 3H), 2.79–2.67 (m, 6H), 2.67–2.57 (m, 4H), 2.39 (d, *J* = 7.0 Hz, 2H), 2.10 (dt, *J* = 13.7, 4.7 Hz, 2H), 2.00 (d, *J* = 11.8 Hz, 2H), 1.77 (d, *J* = 13.5 Hz, 6H), 1.67 (d, *J* = 11.8 Hz, 2H), 1.63 (s, 2H), 1.28–1.22 (m, 2H).

#### 4-(4-((4-(1-(4-((5-Chloro-4-((2-(dimethylphosphoryl)phenyl)amino)pyrimidin-2-yl)amino)-3-methoxyphenyl)piperidin-4-yl)piperazin-1-yl)methyl)piperidin-1-yl)-*N*-(2,6-dioxopiperidin-3-yl)benzamide (**9r**)

White solid. HRMS (ESI) *m*/*z*: calcd, 897.4096 for C_46_H_58_ClN_10_O_5_P [M + H]^+^; found, 897.4107. ^1^H NMR (400 MHz, DMSO-*d*_6_): δ 11.16 (s, 1H), 10.82 (s, 1H), 8.49 (d, *J* = 8.4 Hz, 1H), 8.44 (d, *J* = 8.3 Hz, 1H), 8.18 (s, 2H), 8.06 (d, *J* = 5.3 Hz, 2H), 7.74 (d, *J* = 8.4 Hz, 2H), 7.53 (d, *J* = 14.1 Hz, 1H), 7.43–7.30 (m, 2H), 7.10 (t, *J* = 7.8 Hz, 1H), 6.95 (d, *J* = 8.8 Hz, 2H), 6.63 (d, *J* = 2.5 Hz, 1H), 6.47 (d, *J* = 8.8 Hz, 1H), 4.73 (d, *J* = 12.9 Hz, 1H), 3.93 (s, 4H), 3.89–3.81 (m, 8H), 3.76 (s, 6H), 3.73 (s, 3H), 2.85–2.76 (m, 2H), 2.72 (dd, *J* = 20.7, 4.0 Hz, 3H), 2.66 (s, 3H), 2.63 (s, 2H), 2.55 (dd, *J* = 6.2, 2.7 Hz, 2H), 2.47 (s, 4H), 2.20 (d, *J* = 6.5 Hz, 2H), 2.11 (t, *J* = 13.0, Hz, 2H), 1.98–1.87 (m, 3H), 1.76 (d, *J* = 13.5 Hz, 6H), 1.57 (td, *J* = 12.9, 6.0 Hz, 3H), 1.16 (d, *J* = 12.3 Hz, 2H).

#### 3-((4-(4-(2-(4-(1-(4-((5-Chloro-4-((2-(dimethylphosphoryl)phenyl)amino)pyrimidin-2-yl)amino)-3-methoxyphenyl)piperidin-4-yl)piperazin-1-yl)-2-oxoethyl)piperazin-1-yl)phenyl)amino)piperidine-2,6-dione (**9s**)

Brown solid. HRMS (ESI) *m*/*z*: calcd, 898.4048 for C_45_H_57_ClN_11_O_5_P [M + H]^+^; found, 898.4052. ^1^H NMR (400 MHz, DMSO-*d*_6_): δ 11.17 (s, 1H), 10.76 (s, 1H), 8.49 (s, 1H), 8.19–7.86 (m, 2H), 7.46–7.81 (m, 5H), 7.25–6.86 (m, 4H), 6.78 (d, *J* = 8.3 Hz, 1H), 6.72–6.54 (m, 2H), 6.49 (d, *J* = 8.8 Hz, 1H), 5.44 (s, 1H), 4.20 (s, 3H), 3.67–3.51 (m, 6H), 3.02 (s, 9H), 2.79 (d, *J* = 15.6 Hz, 7H), 2.05 (d, *J* = 19.4 Hz, 5H), 1.76 (d, *J* = 13.5 Hz, 6H), 1.21 (d, *J* = 18.5 Hz, 2H).

#### 3-((4-(4-(2-(4-(1-(4-((5-Chloro-4-((2-(dimethylphosphoryl)phenyl)amino)pyrimidin-2-yl)amino)-3-methoxyphenyl)piperidin-4-yl)piperazin-1-yl)ethyl)piperidin-1-yl)phenyl)amino)piperidine-2,6-dione (**9t**)

Brown solid. HRMS (ESI) *m*/*z*: calcd, 883.4303 for C_46_H_60_ClN_10_O_4_P [M + H]^+^; found, 883.4344. ^1^H NMR (400 MHz, DMSO-*d*_6_): δ 11.16 (s, 1H), 8.49 (s, 1H), 8.22–8.11 (m, 1H), 8.07 (s, 1H), 7.53 (d, *J* = 13.8 Hz, 1H), 7.47–7.19 (m, 2H), 7.13–6.98 (m, 1H), 6.88 (d, *J* = 9.0 Hz, 1H), 6.75 (d, *J* = 8.2 Hz, 1H), 6.69–6.52 (m, 1H), 6.48 (d, *J* = 8.7 Hz, 1H), 4.18 (s, 1H), 3.75 (d, *J* = 6.8 Hz, 4H), 3.59 (d, *J* = 11.8 Hz, 3H), 3.04 (t, *J* = 7.2 Hz, 3H), 2.76–2.57 (m, 6H), 1.94 (s, 2H), 1.76 (d, *J* = 13.6 Hz, 6H), 1.55 (m, 4H), 1.29–1.07 (m, 3H).

#### 3-((4-(4-(2-(4-(1-(4-((5-Chloro-4-((2-(dimethylphosphoryl)phenyl)amino)pyrimidin-2-yl)amino)-3-methoxyphenyl)piperidin-4-yl)piperazin-1-yl)-2-oxoethyl)piperidin-1-yl)phenyl)amino)piperidine-2,6-dione (**9u**)

Brown solid. HRMS (ESI) *m*/*z*: calcd, 897.4096 for C_46_H_58_ClN_10_O_5_P [M + H]^+^; found, 897.4085. ^1^H NMR (400 MHz, DMSO-*d*_6_): δ 11.16 (s, 1H), 9.66 (d, *J* = 10.9 Hz, 1H), 8.48 (s, 1H), 8.06 (s, 2H), 7.95 (d, *J* = 8.4 Hz, 2H), 7.68 (d, *J* = 8.3 Hz, 2H), 7.58–7.24 (m, 12H), 7.13–7.20 (m, 3H), 6.87 (t, *J* = 11.4 Hz, 2H), 6.75 (d, *J* = 8.6 Hz, 1H), 6.66–6.56 (m, 3H), 6.47 (d, *J* = 8.7 Hz, 2H), 3.76 (s, 8H), 3.48 (s, 8H), 2.75–2.57 (m, 10H), 2.55 (s, 4H), 2.28 (d, *J* = 6.5 Hz, 3H), 2.22 (d, *J* = 6.9 Hz, 2H), 1.98 (d, *J* = 3.3 Hz, 1H), 1.90–1.79 (m, 4H), 1.76 (d, *J* = 13.5 Hz, 6H), 1.59–1.51 (m, 2H), 1.29 (s, 2H).

#### 3-((4-(4-(4-(1-(4-((5-Chloro-4-((2-(dimethylphosphoryl)phenyl)amino)pyrimidin-2-yl)amino)-3-methoxyphenyl)piperidin-4-yl)piperazine-1-carbonyl)piperidin-1-yl)phenyl)amino)piperidine-2,6-dione (**9v**)

Brown solid. HRMS (ESI) *m*/*z*: calcd, 883.3939 for C_45_H_56_ClN_10_O_5_P [M + H]^+^; found, 883.3927. ^1^H NMR (400 MHz, DMSO-*d*_6_): δ 11.17 (s, 1H), 10.76 (s, 1H), 9.68 (d, *J* = 8.6 Hz, 1H), 8.49 (s, 1H), 8.21–7.86 (m, 3H), 7.72 (d, *J* = 8.4 Hz, 2H), 7.63–7.21 (m, 7H), 7.21–6.97 (m, 2H), 6.97–6.73 (m, 4H), 6.73–6.32 (m, 5H), 5.43 (s, 1H), 4.20 (s, 2H), 3.83 (s, 10H), 2.69 (s, 13H), 2.50 (p, *J* = 1.8 Hz, 51H), 2.13–1.90 (m, 5H), 1.75 (t, *J* = 15.7 Hz, 6H).

### Molecular Modeling

To build in silico models of ROS1 in complex with ceritinib and brigatinib, the initial structure of the protein was retrieved from the crystal structure of the ROS1 kinase domain in complex with crizotinib (PDB code 3ZBF).^7^ The protein structure was then prepared using the protein preparation wizard of Schrodinger Suite 2023-4 by removing water molecules and ions.^25^ Hydrogen atoms were added to the protein consistent with the physiological pH. The protein–ligand complex was energy minimized with an rmsd cutoff value of 0.3 Å for all heavy atoms. Structures of ceritinib and brigatinib molecules were prepared using the builder module of the Schrodinger Suite followed by energy minimization. The crystal ligand binding site was selected as the ligand binding site for all the compounds. The receptor grids were generated for the prepared protein structure encompassing the active site, and the center of the receptor grids was placed at the geometric center of the crystal ligand in a cubic grid box. All the docking calculations were done in the extra precision mode of Glide with the default parameters followed by post docking minimization. Drummond et al.^26,27^ have recently developed an in silico tool that includes multiple protocols for modeling a PROTAC-mediated ternary complex. We used their most successful protocol [Method 4B]^26^ to predict the ternary structure using MOE. The initial structures for ROS1 and the CRBN E3 ligase were obtained from the crystal structures; PDB codes: 3ZBF(7) and 4CI2(28) for ROS1 and CRBN E3 ligase, respectively. We chose the best degrader **9o** as the representative structure to build the ternary complex. Solvent front mutation G2032R was considered for ROS1. As reported previously,^26,27^ ternary structures were built in different phases. Finally, PROTACs based on the maximum double cluster population was chosen as the representative model of the ternary structure.

### Cell Lines and Reagents

Ba/F3 immortalized murine bone marrow–derived pro-B cells were obtained from Creative Biogene (Shirley, NY) and cultured in RPMI supplemented with 10% FBS and IL3 (0.5 ng/mL). Ba/F3 cells expressing ROS1 variants were maintained in RPMI supplemented with 10% FBS and puromycin (0.7 mg/mL). Crizotinib, ceritinib, brigatinib, repotrectinib, and lorlatinib were purchased from MedChem Express. Each compound was dissolved in DMSO and serially diluted in RPMI for cell culture experiments.

### Antiproliferation Assays

To assess the 72 h effect of drug treatment on cell proliferation, 2000 to 3000 cells were plated in replicates of three in 96-well plates. The plates were placed in an incubator at 37 °C, 5% CO_2_ for 72 h. Following drug treatments, the cells were incubated with the Alamar Blue Assay reagent (BioRad), 5 μL each well for 4 h at 37 °C and 5% CO_2_. Luminescence was measured on a multiplate reader and analyzed. The data were graphically displayed using GraphPad Prism version 10.0 (GraphPad Software). IC_50_ values were determined using a nonlinear regression model with a sigmoidal dose response in GraphPad. The data were collected based on triplicate experiments, and presented as mean ± SD.

### Cloning and Creation of Stable Ba/F3 Cell Lines

cDNA encoding CD74-ROS1 or mutant CD74–ROS1 was cloned into retroviral expression vectors (pLenti), and viruses were generated in HEK293T cells by transfecting with packaging plasmids. The resulting virus was used to infect Ba/F3 cells. After retroviral infection, Ba/F3 cells were selected by incubation with puromycin (0.7 mg/mL) for 2 weeks. For Ba/F3 cells infected by the CD74–ROS1 variants, IL3 was withdrawn from the culture medium at least 2 weeks before the experiments.

### Immunoblotting for Cellular Expression of the ROS1 Kinase and ROS1 Phosphorylation

Protein levels were analyzed using a WES instrument according to the WES User Guide from ProteinSimple in duplicate experiments. The samples were mixed with the fluorescent 5 master mix (ProteinSimple) and then heated at 95 °C for 5 min. Boiled samples, biotinylated protein ladder, antibody diluent, Streptavidin-HRP, primary antibodies, ProteinSimple horseradish peroxidase conjugated antirabbit or antimouse secondary antibodies, luminol-peroxide, and wash buffer were loaded into the WES plate (Wes 12–230 kDa Prefilled Plates with Split Buffer, ProteinSimple). The plates and capillary cartridges were loaded into the WES instrument, and protein separation, antibody incubation, and imaging were performed according to manufacturer’s instructions. Compass software (ProteinSimple) was used to acquire the data and to generate the virtual blot image reconstruction and chemiluminescence signal intensity electropherograms. The electropherogram shows the intensity detected along the length of the capillaries and shows automatically detected peaks that can be quantified by calculation of the AUC. Protein levels are expressed as the AUC of the peak chemiluminescence intensity. Antibodies against phospho-ROS1(Tyr2274/2334) (E8F5J), ROS1 (D4D6(R)) were obtained from Cell Signaling Technology.

### Proteomics Study

H2228 cells were treated with brigatinib or **9o** at 100 nM for 24 h. Cell pellets were dissolved in 1 mL of RIPA buffer, 10 μL of phosphatase inhibitor, and 10 μL of protease inhibitor. Proteins were extracted and concentration was determined by the BCA assay. The protein samples were digested with sequencing grade modified trypsin (Promega Corp; Madison, WI) according to the manufacturer’s instructions. The peptides were labeled using the TMT Isobaric Mass Tagging kit (Thermo Fisher Scientific; # 90,060–90,061; Waltham, MA). The labeled peptide aliquots were then pooled together for subsequent fractionation and analysis. The TMT-labeled peptide mixtures were separated by an automated Easy-nLC1000 system coupled with a Q-Exactive spectrometer (Thermo Finnigan, Waltham, MA). The identification of proteins was performed using Proteome Discoverer software v2.3 (Thermo Fisher Scientific; Waltham, MA) and Mascot v2.6 (Matrix Science, Boston, MA), based on “Uniprot Human” database. The proteins that were identified on the basis of at least one unique peptide were included in the data. The ratios of TMT reporter ion abundance in **9o**-treated cells and inhibitor-treated cells were used to calculate protein relative abundance.

### Metabolic Stability Studies

Pooled human and rat microsomes were prepared and stored at −80 °C prior to use. A master-mix containing microsome, phosphate buffer, and test compound (**9** series) solution was made as follows: (1) 3 μL of microsome (20 mg/mL) was diluted with mixed 30 μL of potassium phosphate buffer (pH 7.4; 10×) and 246 μL of water; (2) 3 μL of 25 μM drug solution was added to the microsome; (3) the master solution was prewarmed at 37 °C for 5 min. NADPH solution A (15 μL) and NADPH solution B (3 μL) from Promega were added to the above-mentioned master solution to initiate the reaction. The final concentration of **9** compounds in the reaction system was 250 nM. An aliquot of 20 μL was pipetted from the reaction solution and stopped by the addition of 180 μL of cold MeCN containing 25 ng of Tamoxifen-(*N*,*N*-dimethyl-13C2)-15N as an internal standard at the designated time points (0, 5, 15, 30, 45, and 60 min). The incubation solution was vortexed-mixed (800 rpm/10 min) and centrifuged at 3500 rpm for 10 min to precipitate proteins. The supernatant was collected and used for the LC/MS/MS analysis. The natural log of the remaining percentage of the test compound was plotted against time and the gradient of the line was determined.

### Pharmacokinetics and In Vivo Xenograft Studies

#### Mouse Efficacy Study

All the procedures related to animal handling, care, and treatment in this study were performed according to guidelines approved by the Institutional Animal Care and Use Committee (IACUC) of Xavier University of Louisiana following the guidance of the AAALAC. Ba/F3-ROS1 and Ba/F3-ROS1-G2032R cells were maintained in RPMI 1640 medium supplemented with 10% fetal bovine serum. The cells growing in an exponential growth phase were harvested and counted for tumor inoculation. Female NOD/SCID mice 6–8 weeks of age were allowed to adapt to laboratory conditions for 10 days prior to dosing and were allowed food and water ad libitum. Each mouse was inoculated subcutaneously in the right flank with Ba/F3 CD74-ROS1 or Ba/F3 SL34A2-ROS1 G2032R cells (1 × 10^6^) in 0.2 mL of PBS mixed with Matrigel (50:50) for tumor development. Tumor-bearing mice were dosed with the compound at 10 or 30 mg/kg QD po or vehicle (Propylene glycol/Solutol/20% HP-β-CD in water (20:5:75) when the tumor volume reached 200–300 mm^3^ for 3 weeks. Body weight and tumor volume were measured every 2 days using a caliper and other clinical observations for treatment and tumor burden effects. At the end point of the efficacy study, the mice were euthanized according to the IACUC guidelines. Terminal samples that were collected were blood for PK, and tumor for PK and PD analysis (Western blot of total ROS1 and *p*-ROS1). Tumor volume collected over the course of the study were used to calculate TGI using the formula: TGI % = [1 – Δ*T*/Δ*C*] × 100, where Δ*T* is the volume change for treated tumors and Δ*C* is the volume change for control tumors.

#### Pharmacokinetics Study

Four to 6 weeks old Sprague–Dawley rats [Crl: CD (SD)] (SPF/VAF) were purchased from Charles River (Wilmington MA). Animals were quarantined/acclimated for 2–6 weeks prior to dose initiation. Sprague–Dawley rats were given oral administration of test compounds (20% PG, 5% Solutol, and 75% of 20% HP-β-CD in water) at different dose levels. After treatment with the compounds, whole blood was collected from each study animal by puncture of the tail vein (0.4 mL of whole blood from rats) into tubes containing K2EDTA at each time point. The plasma samples were divided into 2 aliquots (approximately equal in volume) and stored frozen at −75 ± 15 °C within 2 h of the collection until analysis.

The stock solution of the test compound (0.01 M) in DMSO was prepared and stored at −20 ± 5 °C. The desired serial concentrations of working reference analyte solutions were achieved by diluting a stock analyte solution with 50% acetonitrile (0.1% formic acid) in water solution. The bioanalytical LC–MS/MS method was developed for quantitative determination of each test compound in Sprague–Dawley rat plasma with acceptable selectivity, sensitivity, calibration curve, precision, and accuracy. The analytical method is applicable for the determination of test compounds in Sprague–Dawley rat plasma with a lower limit of quantification of 10.0 ng/mL and an upper limit of quantification of 5000 ng/mL. Five microliters of diluted supernatant was injected into the LC–MS/MS system (TSQ Quantis) and a SynergiTM 4 μm Fusion-RP 80 Å (50 × 2 mm) column using gradient elution and a Triple Quad mass spectrometer) for quantitative analysis. The mobile phases used were 0.1% formic acid in deionized water and 0.1% formic acid in acetonitrile. The pharmacokinetic parameters *T*_1/2_ (the biological half-life), *C*_max_ (maximal concentration), *T*_max_ (time at which *C*_max_ is observed), and AUC (area under the plasma concentration–time curve) were calculated from the plasma concentration versus time data using WinNonlin (PhoenixTM, version 8.1, Mountain View, CA, USA).

## Supporting Information Available

Pharmacokinetic studies in rat, HPLC traces, in silico physicochemical and pharmacokinetics properties; and names, SMILES, molecular formulas, and biochemical/biological data of compounds (PDF)
Molecular formula strings and related data (CSV)

## Abbreviations

ALK: anaplastic lymphoma kinase
AUC: area under the curve
Boc: *t*-butyloxy carbonyl
CGI: cell growth inhibition
*C*_max_: highest concentration of a drug in the blood or target organ
CRBN: cereblon
DC_50_: the half-maximal degradation concentration
DCM: dichloromethane
DIEA (DIPEA): *N*,*N*-diisopropylethylamine
DMA: dimethylacetamide
*D*_max_: the maximal degradation rate
DMP: Dess–Martin periodinane
DMPO: dimethyl phosphine oxide
EA (EtOAc): ethyl acetate
LC–MS: liquid chromatography–mass spectrometry
MeCN: methyl cyanide
MeOH: methyl alcohol
NaBH_3_CN: sodium cyanoborohydride
NMP: *N*-methyl-2-pyrrolidone
NMR: nuclear magnetic resonance
NSCLC: nonsmall cell lung cancer
PE: petroleum ether
PROTAC: proteolysis targeting chimera
rmsd: root-mean-square deviation
ROS1: c-ros oncogene 1
SLC34A2: solute carrier family 34 member 2
TFA: trifluoroacetic acid
TGI: tumor growth inhibition
THF: tetrahydrofuran
TKI: tyrosine kinase inhibitor
*T*_max_: the time taken for a drug to reach the maximum concentration (*C*_max_)
