# Supplementary material for: Discovery of Oral Degraders of the ROS1 Fusion Protein with Potent Activity against Secondary Resistance Mutations
Source: J Med Chem. 2024 Oct 3;67(20):18098–123. doi: 10.1021/acs.jmedchem.4c01205 (PMC11513893; doi:10.1021/acs.jmedchem.4c01205)
Supplement: Supplementary file 1 — jm4c01205_si_001.pdf [file jm4c01205_si_001.pdf]

Supporting Information

Discovery of Oral Degraders of ROS1 Fusion Protein with Potent Activity against Secondary Resistance Mutations

Xianyou Peng[1,2], Shanchun Guo[1,2], Shilong Zheng[1,2], Ahamed Hossain[1,2], Changde Zhang[1,2], Madhusoodanan Mottamal[1,2], Elena Skripnikova[2,3], Peng Ma[2,3], Kindy Martinez-Carter[2,3], Qiang Zhang[1,2], Faisal Abedin[4], Thomas Huckaba[4], Guangdi Wang[1,2] *

1. Department of Chemistry, Xavier University of Louisiana, New Orleans, LA 70125
2. RCMI Cancer Research Center, Xavier University of Louisiana, New Orleans, LA 70125
3. College of Pharmacy, Xavier University of Louisiana, New Orleans, LA 70125
4. Department of Biology, Xavier University of Louisiana, New Orleans, LA. 70125

Corresponding author: gwang@xula.edu

**Contents**	**Page**
Pharmacokinetic studies in rat	S2
HPLC Traces	S8
In Silico physicochemical and pharmacokinetics properties	S14
Names, SMILES, molecular formula and biochemical/biological data of compounds	S15

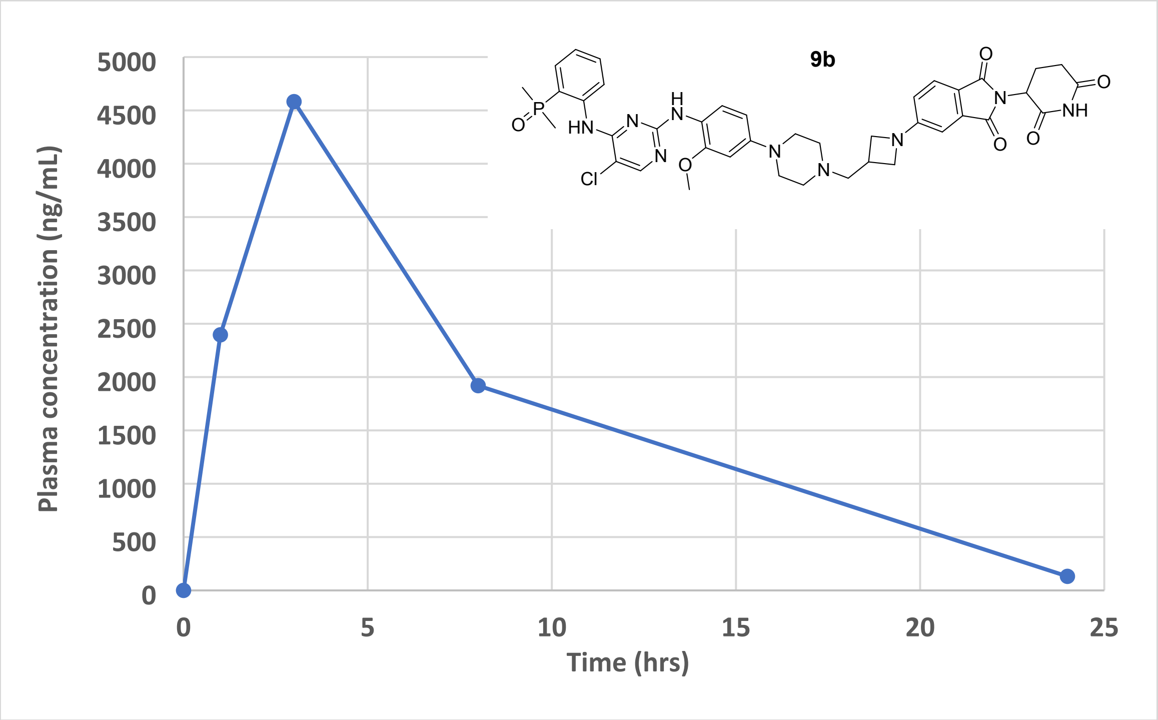

Figure S1. Pharmacokinetic profile of compound **9b** in rat at an oral dose of 10 mg/kg

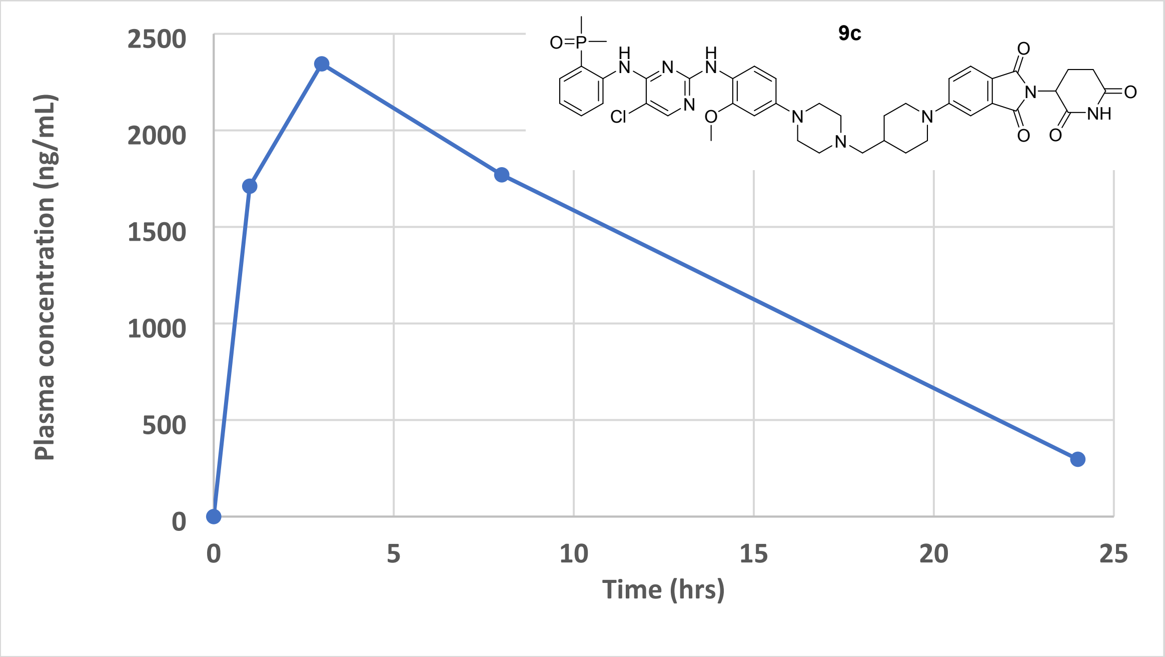

Figure S2. Pharmacokinetic profile of compound **9c** in rat at an oral dose of 10 mg/kg

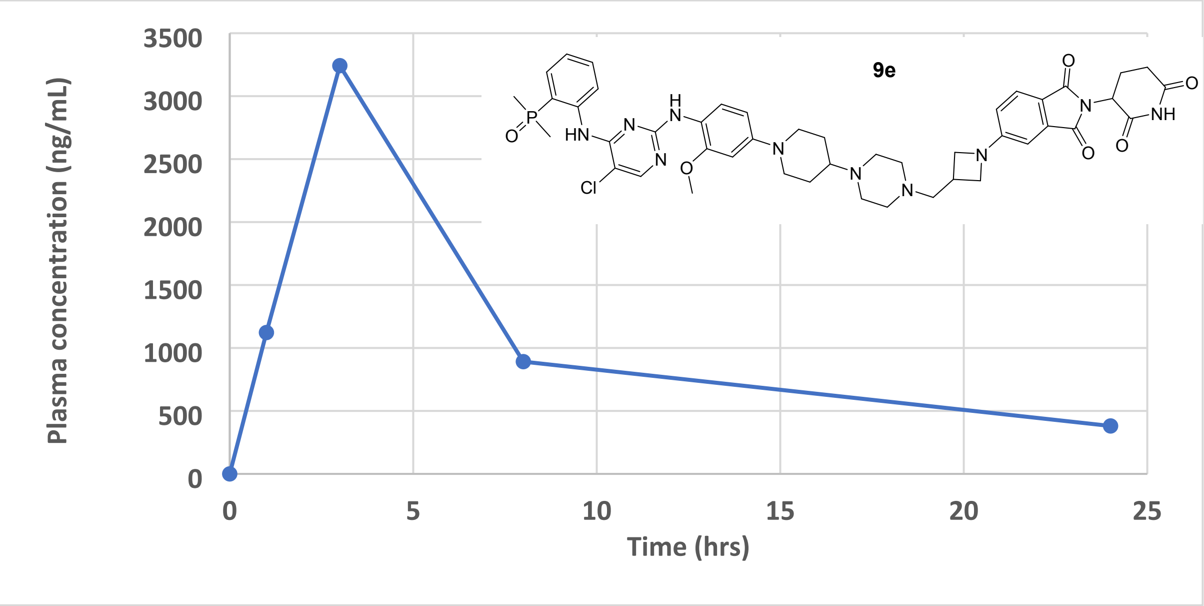

Figure S3. Pharmacokinetic profile of compound **9e** in rat at an oral dose of 10 mg/kg

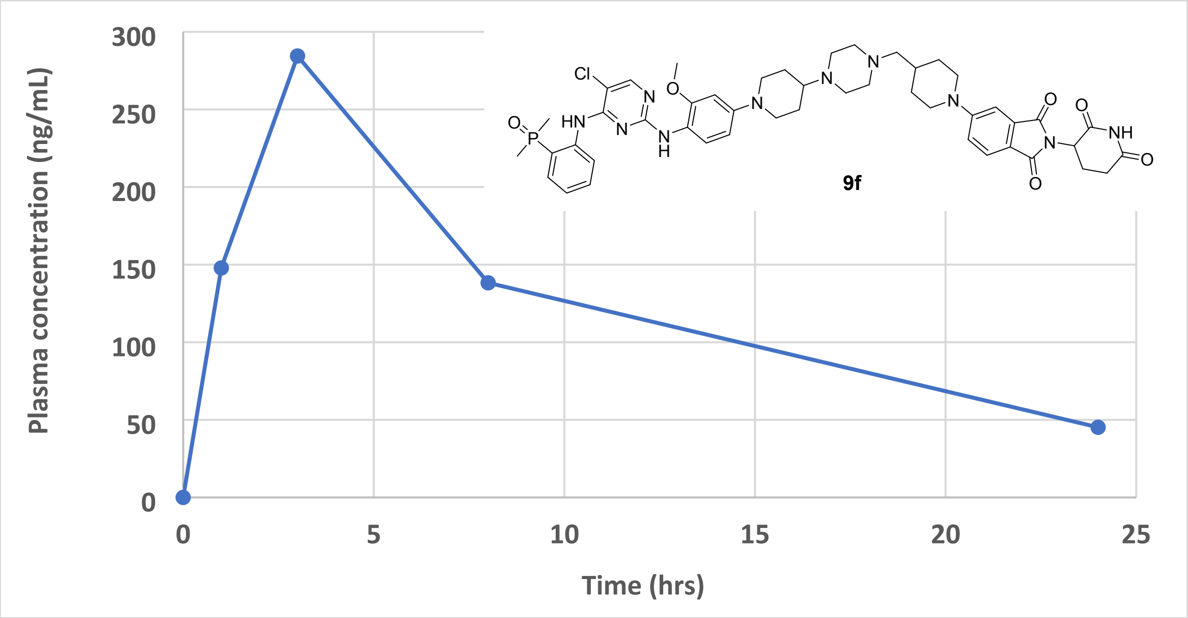

Figure S4. Pharmacokinetic profile of compound **9f** in rat at an oral dose of 10 mg/kg

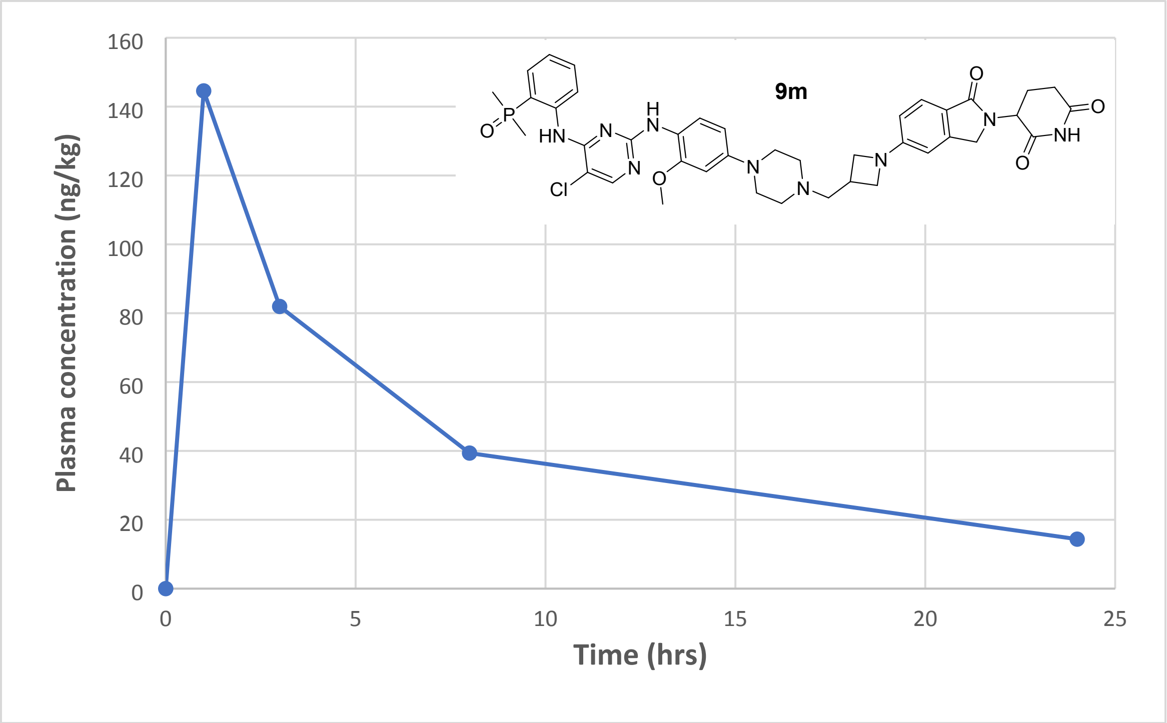

Figure S5. Pharmacokinetics of compound 9m in rat at an oral dose of 5 mg/kg.

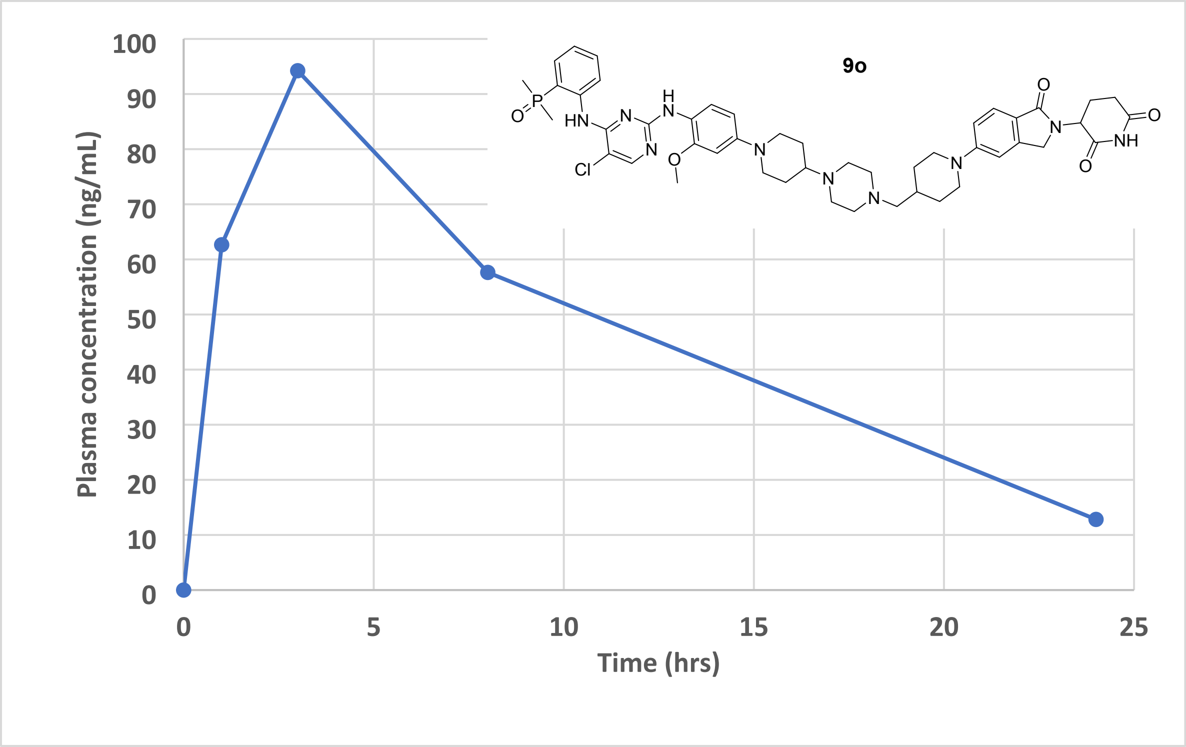

Figure S6. Pharmacokinetics of compound **9o** in rat at an oral dose of 5 mg/kg.

HPLC Traces

9b

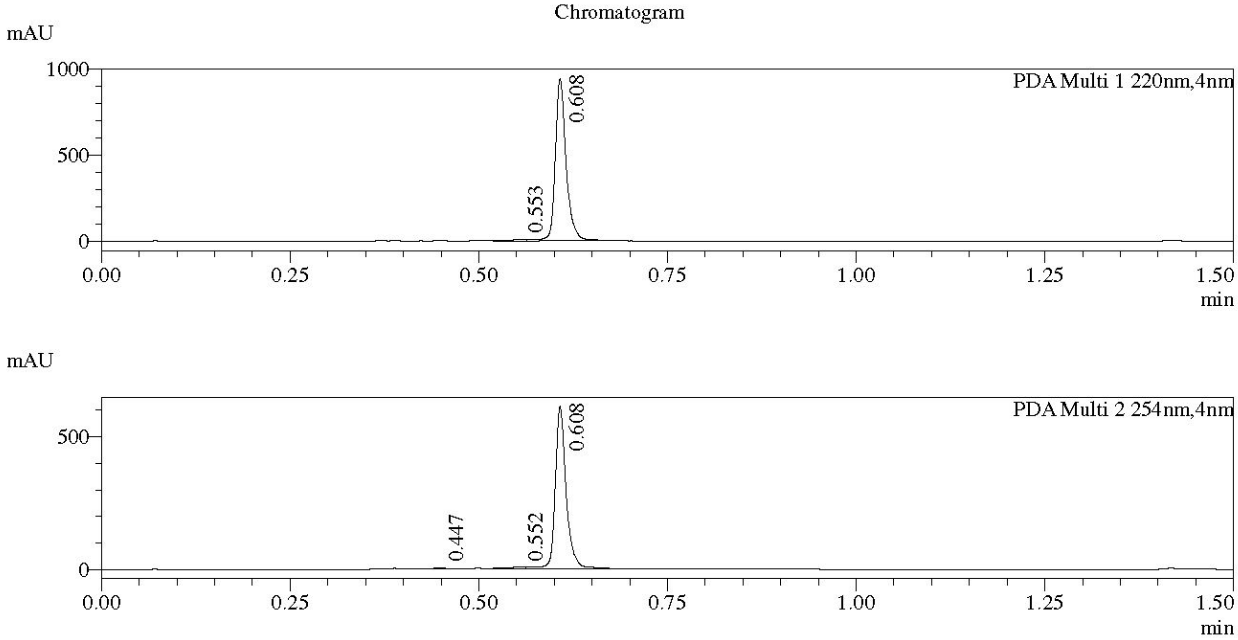

Peak Table

PDA Ch1 220nm

Peak#	Ret. Time	Height	Height%	USP Width	Area	Area%
1	0.553	10526	1.104	0.064	16656	1.739
2	0.608	943087	98.896	0.027	940912	98.261

Peak Table

PDA Ch2 254nm

Peak#	Ret. Time	Height	Height%	USP Width	Area	Area%
1	0.447	5867	0.942	0.026	5081	0.809
2	0.552	7381	1.185	0.072	12585	2.004
3	0.608	609845	97.874	0.027	610371	97.187

9c

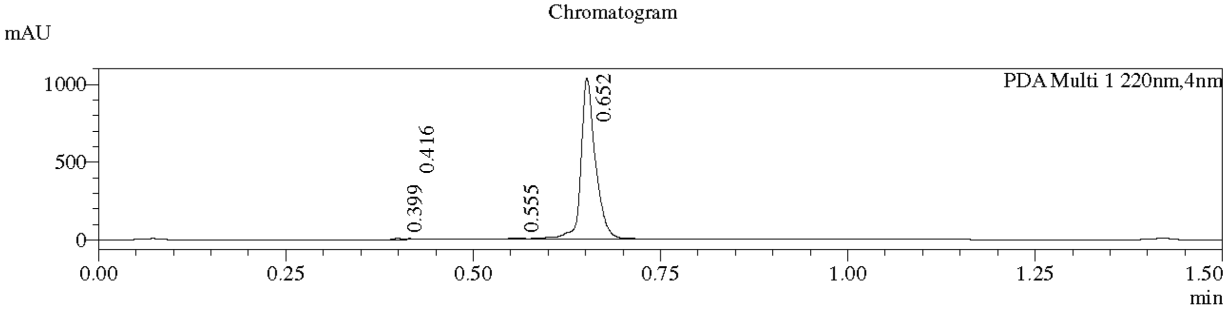

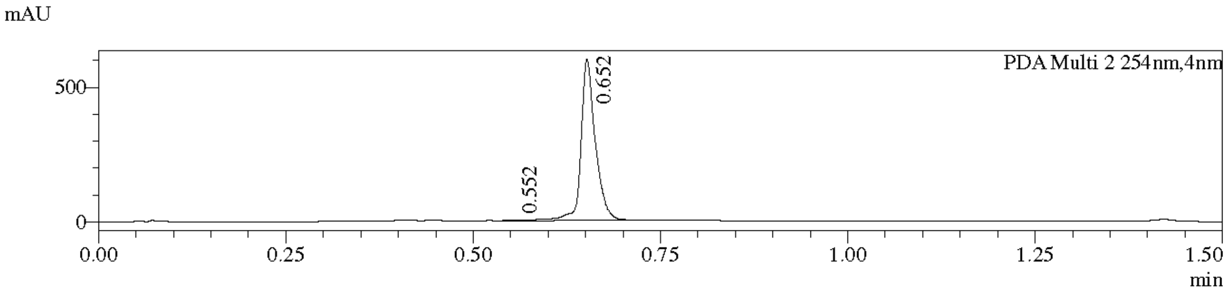

Peak Table

PDA Ch1 220nm

Peak#	Ret. Time	Height	Height%	USP Width	Area	Area%
1	0.399	7176	0.678	0.029	5708	0.404
2	0.416	6283	0.594	0.035	5543	0.392
3	0.555	6563	0.620	0.061	10696	0.757
4	0.652	1038182	98.108	0.034	1391074	98.447

Peak Table

PDA Ch2 254nm

Peak#	Ret. Time	Height	Height%	USP Width	Area	Area%
1	0.552	3217	0.534	0.105	7020	0.871
2	0.652	598768	99.466	0.034	798714	99.129

9e

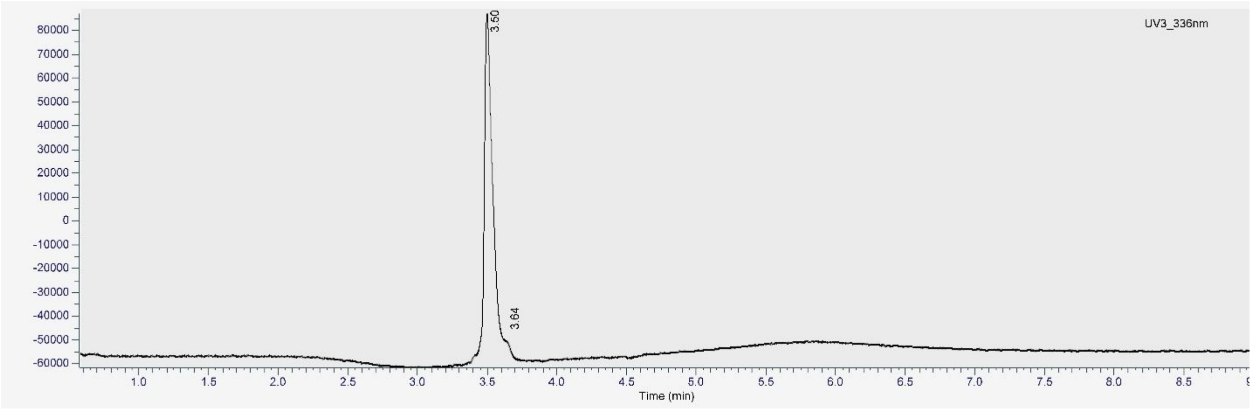

Peak #	RT (min)	Width (min)	Peak Height(mAU)	Peak Area (mAU*s)	% Area
1	3.50	0.12	140500	521928	97.97
2	3.64	0.09	3710	10793	2.03

9f

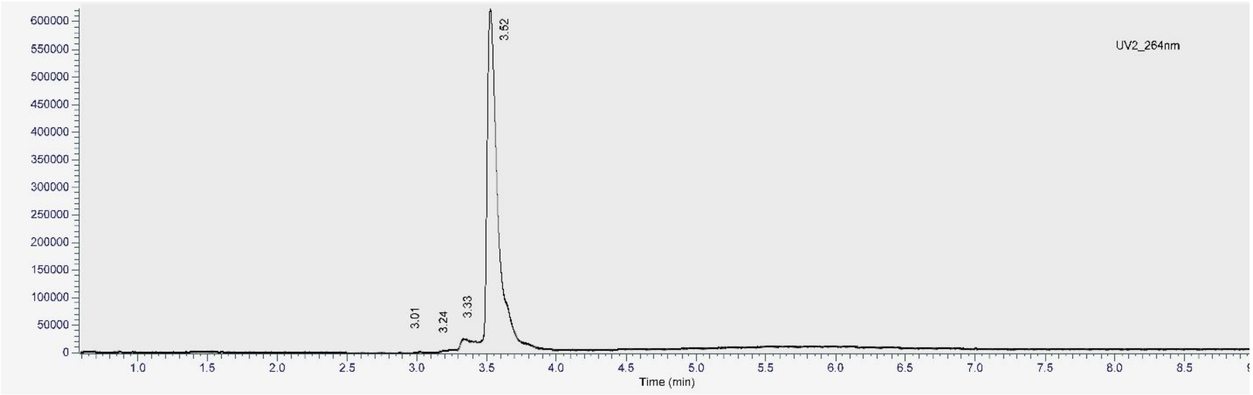

Peak #	RT (Min)	Peak Area	Peak Height	Baseline Width	% Area
1	3.012	2635.05	987.0737705	0.090502793	0.09
2	3.243333	5446.28	1494.837404	0.101739841	0.18
3	3.334667	35220.1	11805.10178	0.081703911	1.19
4	3.525333	2915080	603293.9343	0.226256983	98.54

9m

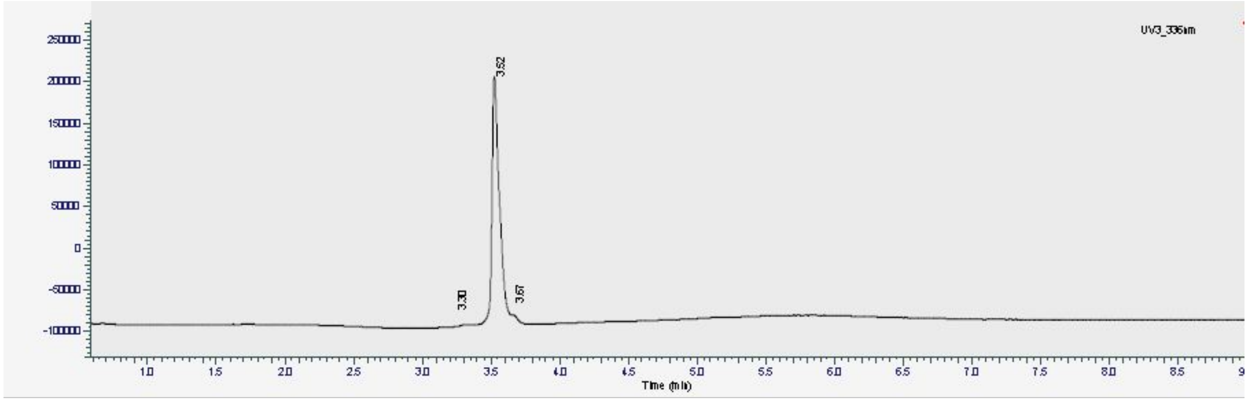

Peak #	RT (Min)	Peak Area	Peak Height	Baseline Width	% Area
1	3.300667	4565.39	1397.681658	0.148206751	0.41
2	3.524	1093717	294803.499	0.183776371	98.17
3	3.673333	15826.6	5566.40687	0.0592827	1.42

9o

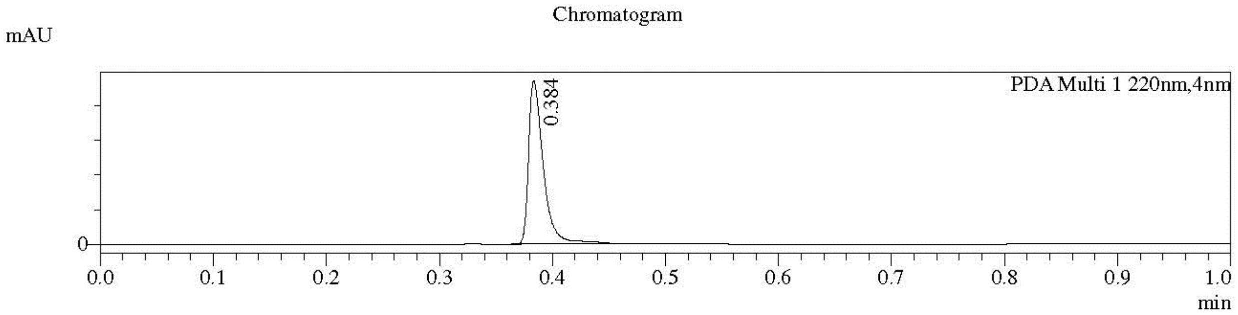

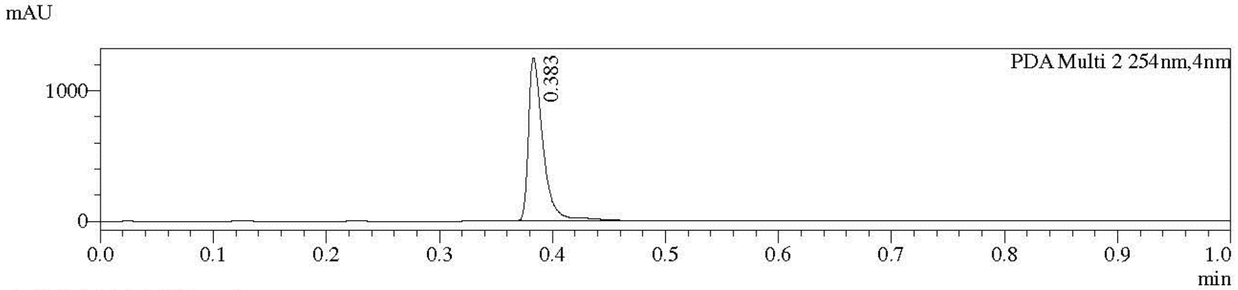

Peak Table

PDA Ch1 220nm

Peak#	Ret. Time	Height	Height%	USP Width	Area	Area%
1	0.384	2349770	100.000	0.025	2157940	100.000

Peak Table

PDA Ch2 254nm

Peak#	Ret. Time	Height	Height%	USP Width	Area	Area%
1	0.383	1248167	100.000	0.024	1125881	100.000

Table S1: In Silico Physicochemical and Pharmacokinetics of orally administered compounds.

Compound	MW	PSA	HBD	HBA	RuleOfFive	QplogPo/w	QPPCaco	QPlogBB	QPPMDCK	QPlogKp	QPlogKhsa	HOA	SASA
**9b**	812.263	205.95	2	16.75	2	4.49	8.71	-2.96	6.58	-6.39	1.01	1	1222.54
**9c**	840.317	205.85	2	17.75	2	4.95	9.08	-2.96	6.86	-6.39	1.02	1	1255.82
**9e**	895.396	211.23	2	18.75	3	5.08	2.11	-2.80	1.57	-8.49	1.26	1	1351.70
**9f**	923.450	211.08	2	19.75	3	5.17	2.19	-2.82	1.63	-8.47	1.26	1	1390.96
**9m**	798.280	180.01	2	16.75	3	5.11	18.86	-2.51	15.19	-5.73	1.02	1	1230.70
**9o**	909.466	184.89	2	19.75	3	5.42	4.80	-2.32	3.82	-7.81	1.26	1	1396.91

The descriptors and desired values are molecular weight (MW): < 500 Da, Van der Waals surface area of polar nitrogen and oxygen atoms (PSA): 7-200, hydrogen bond donor (HBD): 0-6, hydrogen bond acceptor (HBA): 2-20.0, Number of violations of Lipinski's Rule Of Five (RuleOfFive): maximum is 4, predicted octanol/water partition coefficient (QPlogPo/w): -2.0-6.5, predicted apparent Caco-2 cell permeability in nm/sec (QPPCaco): <25 poor; >500 great, predicted brain/blood partition coefficient (QPlogBB): -3.0-1.2, predicted apparent MDCK cell permeability in nm/sec (QPPMDCK): <25 poor; >500 great, predicted skin permeability (QPlogKp): -8.0--1.0, prediction of binding to human serum albumin (QPlogKhsa): -1.5-1.5, human oral absorption (HOA): 1, 2, or 3 for low, medium or high, and Solvent accessible surface area (SASA): 300-1000 $Å^2$.

Table S2: Names, SMILES, Molecular Formula and biochemical/biological data of ROS1 degrader compounds studied in this work.

Compound	SMILES	Molecular Formula	CD74-ROS1 IC50 (nM)	ROS1-G2032R IC50 (nM)	ROS1 DC50 (nM)	ROS1 Dmax (%)
7a	O=C1NC(=O)[C@H](CC1)N(C2=O)C(=O)c(c23)c(ccc3)N(CC4)CCC4n5ncc(c5)c(cnc6N)cc6O[C@@H](C)c(c7Cl)c(Cl)ccc7F	C34H30Cl2F1N7O5	264 ± 44	> 5000	310	67%
7b	O=C1NC(=O)[C@H](CC1)N(C2=O)C(=O)c(c23)c(ccc3)N4CC(C4)CN(CC5)CCC5n6ncc(c6)c(cnc7N)cc7O[C@@H](C)c(c8Cl)c(Cl)ccc8F	C38H37Cl2F1N8O5	2700 ± 254	> 5000	>1000	<5%
7c	O=C1NC(=O)[C@H](CC1)N(C2=O)C(=O)c(c23)c(ccc3)N(CC4)CCC4CN(CC5)CCC5n6ncc(c6)c(cnc7N)cc7O[C@@H](C)c(c8Cl)c(Cl)ccc8F	C40H41Cl2F1N8O5	350 ± 106	> 5000	368	76%
7d	O=C1NC(=O)[C@H](CC1)N(C2=O)C(=O)c(c23)c(ccc3)N(CC4)CCC4CCN(CC5)CCC5n6ncc(c6)c(cnc7N)cc7O[C@@H](C)c(c8Cl)c(Cl)ccc8F	C41H43Cl2F1N8O5	106 ± 29	> 5000	145	78%
7e	O=C1NC(=O)[C@H](CC1)N(C2=O)C(=O)c(c23)cc(cc3)N(CC4)CCC4n5ncc(c5)c(cnc6N)cc6O[C@@H](C)c(c7Cl)c(Cl)ccc7F	C34H30Cl2F1N7O5	155 ± 21	2909 ± 742	203	53%
7f	O=C1NC(=O)[C@H](CC1)N(C2=O)C(=O)c(c23)cc(cc3)N4CC(C4)CN(CC5)CCC5n6ncc(c6)c(cnc7N)cc7O[C@@H](C)c(c8Cl)c(Cl)ccc8F	C38H37Cl2F1N8O5	569 ± 73	> 5000	622	24%
7g	O=C1NC(=O)[C@H](CC1)N(C2=O)C(=O)c(c23)cc(cc3)N(CC4)CCC4CN(CC5)CCC5n6ncc(c6)c(cnc7N)cc7O[C@@H](C)c(c8Cl)c(Cl)ccc8F	C40H41Cl2F1N8O5	15 ± 4	>5000	21	85%
7h	O=C1NC(=O)[C@H](CC1)N(C2=O)C(=O)c(c23)cc(cc3)N(CC4)CCC4CCN(CC5)CCC5n6ncc(c6)c(cnc7N)cc7O[C@@H](C)c(c8Cl)c(Cl)ccc8F	C41H43Cl2F1N8O5	842 ± 172	>5000	>1000	11%
7i	Fc1ccc(Cl)c(c1Cl)[C@H](C)Oc2cc(cnc2N)c(c3)cnn3C4CCN(CC4)CC(CC5)CCN5CC6CCN(CC6)c(cc7)cc(c78)C(=O)N(C8=O)[C@@H](CC9)C(=O)NC9=O	C46H52Cl2F1N9O5	284 ± 51	>5000	338	32%
8a	c1cccc(c1S(=O)(=O)C(C)C)Nc(n2)c(Cl)cnc2Nc(c(c3)OC(C)C)cc(C)c3C4CCN(CC4)CCNc5cccc(c56)C(=O)N(C6=O)[C@@H]7CCC(=O)NC7=O	C43H49Cl1N8O7S1	176 ± 31	>10000	225	58
8b	c1cccc(c1S(=O)(=O)C(C)C)Nc(n2)c(Cl)cnc2Nc(c(c3)OC(C)C)cc(C)c3C4CCN(CC4)CCCNc5cccc(c56)C(=O)N(C6=O)[C@@H]7CCC(=O)NC7=O	C44H51Cl1N8O7S1	122 ±17	>10000	140	71
8c	c1cccc(c1S(=O)(=O)C(C)C)Nc(n2)c(Cl)cnc2Nc(c(c3)OC(C)C)cc(C)c3C4CCN(CC4)C5CCN(CC5)c6cccc(c67)C(=O)N(C7=O)[C@@H]8CCC(=O)NC8=O	C46H53Cl1N8O7S1	107 ± 16	1257 ± 93	143	82
8d	c1cccc(c1S(=O)(=O)C(C)C)Nc(n2)c(Cl)cnc2Nc(c(c3)OC(C)C)cc(C)c3C4CCN(CC4)C5CCN(CC5)CCNc6cccc(c67)C(=O)N(C7=O)[C@@H]8CCC(=O)NC8=O	C48H58Cl1N9O7S1	183 ± 41	2561 ± 242	302	66

8e	c1cccc(c1S(=O)(=O)C(C)C)Nc(n2)c(Cl)cnc2Nc(c(c3)OC(C)C)cc(C)c3C4CCN(CC4)C5CCN(CC5)CCCNc6cccc(c67)C(=O)N(C7=O)[C@@H]8CCC(=O)NC8=O	C49H60 Cl1N9O 7S1	562 ± 61	>10000	733	28
8f	c1cccc(c1S(=O)(=O)C(C)C)Nc(n2)c(Cl)cnc2Nc(c(c3)OC(C)C)cc(C)c3C4CCN(CC4)C5CCN(CC5)C(C6)CN6c7cccc(c78)C(=O)N(C8=O)[C@@H]9CCC(=O)NC9=O	C49H58 Cl1N9O 7S1	115 ± 22	3662 ± 299	189	67
8g	c1cccc(c1S(=O)(=O)C(C)C)Nc(n2)c(Cl)cnc2Nc(c(c3)OC(C)C)cc(C)c3C4CCN(CC4)C5CCN(CC5)CC(C6)CN6c7cccc(c78)C(=O)N(C8=O)[C@@H]9CCC(=O)NC9=O	C50H60 Cl1N9O 7S1	191 ± 60	950 ± 67	155	77
8h	c1cccc(c1S(=O)(=O)C(C)C)Nc(n2)c(Cl)cnc2Nc(c(c3)OC(C)C)cc(C)c3C4CCN(CC4)CCNc(c5)ccc(c56)C(=O)N(C6=O)[C@@H]7CCC(=O)NC7=O	C43H49 Cl1N8O 7S1	128 ± 39	6632 ± 1804	145	71
8i	c1cccc(c1S(=O)(=O)C(C)C)Nc(n2)c(Cl)cnc2Nc(c(c3)OC(C)C)cc(C)c3C4CCN(CC4)CCCNc(c5)ccc(c56)C(=O)N(C6=O)[C@@H]7CCC(=O)NC7=O	C44H51 Cl1N8O 7S1	93 ± 50	4706 ± 486	88	84
8j	c1cccc(c1S(=O)(=O)C(C)C)Nc(n2)c(Cl)cnc2Nc(c(c3)OC(C)C)cc(C)c3C4CCN(CC4)C5CCN(CC5)c(c6)ccc(c67)C(=O)N(C7=O)[C@@H]8CCC(=O)NC8=O	C46H53 Cl1N8O 7S1	39 ± 8	1534 ± 521	41	88
8k	c1cccc(c1S(=O)(=O)C(C)C)Nc(n2)c(Cl)cnc2Nc(c(c3)OC(C)C)cc(C)c3C4CCN(CC4)C5CCN(CC5)CCNc(c6)ccc(c67)C(=O)N(C7=O)[C@@H]8CCC(=O)NC8=O	C48H58 Cl1N9O 7S1	171 ± 26	8589 ± 1179	204	89
8l	c1cccc(c1S(=O)(=O)C(C)C)Nc(n2)c(Cl)cnc2Nc(c(c3)OC(C)C)cc(C)c3C4CCN(CC4)C5CCN(CC5)CCCNc(c6)ccc(c67)C(=O)N(C7=O)[C@@H]8CCC(=O)NC8=O	C49H60 Cl1N9O 7S1	598 ± 147	>10000	452	54
8m	c1cccc(c1S(=O)(=O)C(C)C)Nc(n2)c(Cl)cnc2Nc(c(c3)OC(C)C)cc(C)c3C4CCN(CC4)C5CCN(CC5)C(C6)CN6c(c7)ccc(c78)C(=O)N(C8=O)[C@@H]9CCC(=O)NC9=O	C49H58 Cl1N9O 7S1	39 ± 34	2772 ± 1084	35	92
8n	c1cccc(c1S(=O)(=O)C(C)C)Nc(n2)c(Cl)cnc2Nc(c(c3)OC(C)C)cc(C)c3C4CCN(CC4)C5CCN(CC5)CC(C6)CN6c(c7)ccc(c78)C(=O)N(C8=O)[C@@H]9CCC(=O)NC9=O	C50H60 Cl1N9O 7S1	713 ± 84	1151 ± 466	586	63
8o	c1cccc(c1S(=O)(=O)C(C)C)Nc(n2)c(Cl)cnc2Nc(c(c3)OC(C)C)cc(C)c3C4CCN(CC4)C5CN(C5)C(C6)CN6c(c7)ccc(c78)C(=O)N(C8=O)[C@@H]9CCC(=O)NC9=O	C47H54 Cl1N9O 7S1	116 ± 21	>10000	122	65
8p	c1cccc(c1S(=O)(=O)C(C)C)Nc(n2)c(Cl)cnc2Nc(c(c3)OC(C)C)cc(C)c3C4CCN(CC4)C5CN(C5)C(CC6)CCN6c(c7)ccc(c78)C(=O)N(C8=O)[C@@H]9CCC(=O)NC9=O	C49H58 Cl1N9O 7S1	40 ± 6	>10000	27	93
8q	c1cccc(c1S(=O)(=O)C(C)C)Nc(n2)c(Cl)cnc2Nc(c(c3)OC(C)C)cc(C)c3C4CCN(CC4)C5CN(C5)CC(C6)CN6c(c7)ccc(c78)C(=O)N(C8=O)[C@@H]9CCC(=O)NC9=O	C48H56 Cl1N9O 7S1	66 ± 6	6122 ± 4068	57	85

8r	c1cccc(c1S(=O)(=O)C(C)C)Nc(n2)c(Cl)cnc2Nc(c(c3)OC(C)C)cc(C)c3C4CCN(CC4)C5CN(C5)CC(CC6)CCN6c(c7)ccc(c78)C(=O)N(C8=O)[C@@H]9CCC(=O)NC9=O	C50H60Cl1N9O7S1	72 ± 3	>10000	77	91
8s	c1cccc(c1S(=O)(=O)C(C)C)Nc(n2)c(Cl)cnc2Nc(c(c3)OC(C)C)cc(C)c3C4CCN(CC4)C(CC5)CCN5C(CC6)CCN6c(c7)ccc(c78)C(=O)N(C8=O)[C@@H]9CCC(=O)NC9=O	C51H62Cl1N9O7S1	106 ± 9	>10000	120	82
8t	c1cccc(c1S(=O)(=O)C(C)C)Nc(n2)c(Cl)cnc2Nc(c(c3)OC(C)C)cc(C)c3C4CCN(CC4)C(CC5)CCN5CC(CC6)CCN6c(c7)ccc(c78)C(=O)N(C8=O)[C@@H]9CCC(=O)NC9=O	C52H64Cl1N9O7S1	147 ± 28	>10000	155	81
9a	c1cccc(P(=O)(C)C)c1Nc(c2Cl)nc(nc2)Nc(c(c3)OC)ccc3N4CCN(CC4)c(cc5)cc(c56)C(=O)N(C6=O)[C@@H](CC7)C(=O)NC7=O	C36H36Cl1N8O6P1	112 ± 17	251 ± 125	133	68
9b	c1cccc(P(=O)(C)C)c1Nc(c2Cl)nc(nc2)Nc(c(c3)OC)ccc3N4CCN(CC4)CC(C5)CN5c(cc6)cc(c67)C(=O)N(C7=O)[C@@H](CC8)C(=O)NC8=O	C40H43Cl1N9O6P1	13 ± 2	153 ± 20	10	96
9c	c1cccc(P(=O)(C)C)c1Nc(c2Cl)nc(nc2)Nc(c(c3)OC)ccc3N4CCN(CC4)CC(CC5)CCN5c(cc6)cc(c67)C(=O)N(C7=O)[C@@H](CC8)C(=O)NC8=O	C42H47Cl1N9O6P1	19 ± 8	236 ± 28	22	95
9d	c1cccc(P(=O)(C)C)c1Nc(c2Cl)nc(nc2)Nc(c(c3)OC)ccc3N4CCC(CC4)N5CCN(CC5)C(C6)CN6c(cc7)cc(c78)C(=O)N(C8=O)[C@@H](CC9)C(=O)NC9=O	C44H50Cl1N10O6P1	61 ± 5	491 ± 48	75	90
9e	c1cccc(P(=O)(C)C)c1Nc(c2Cl)nc(nc2)Nc(c(c3)OC)ccc3N4CCC(CC4)N5CCN(CC5)CC(C6)CN6c(cc7)cc(c78)C(=O)N(C8=O)[C@@H](CC9)C(=O)NC9=O	C45H52Cl1N10O6P1	3.8 ± 0.2	80 ± 27	5.1	97
9f	c1cccc(P(=O)(C)C)c1Nc(c2Cl)nc(nc2)Nc(c(c3)OC)ccc3N4CCC(CC4)N5CCN(CC5)CC(CC6)CCN6c(cc7)cc(c78)C(=O)N(C8=O)[C@@H](CC9)C(=O)NC9=O	C47H56Cl1N10O6P1	11.2 ± 2	27 ± 10	13	99
9g	c1cccc(P(=O)(C)C)c1Nc(c2Cl)nc(nc2)Nc(c(c3)OC)ccc3N4CCC(CC4)C5CCN(CC5)CC(CC6)CCN6c(cc7)cc(c78)C(=O)N(C8=O)[C@@H](CC9)C(=O)NC9=O	C48H57Cl1N9O6P1	7.1 ± 0.9	221 ± 26	11	88
9h	c1cccc(P(=O)(C)C)c1Nc(c2Cl)nc(nc2)Nc(c(c3)OC)ccc3N4CCN(CC4)C5CCN(CC5)CC(CC6)CCN6c(cc7)cc(c78)C(=O)N(C8=O)[C@@H](CC9)C(=O)NC9=O	C47H56Cl1N10O6P1	21 ± 8	115 ± 12	34	85
9i	c1cccc(P(=O)(C)C)c1Nc(c2Cl)nc(nc2)Nc(c(c3)OC)ccc3CC(C4)CN4c(ccc5)c(c56)C(=O)N(C6=O)[C@@H](CC7)C(=O)NC7=O	C36H35Cl1N7O6P1	41 ± 9	244 ± 78	56	79
9j	c1cccc(P(=O)(C)C)c1Nc(c2Cl)nc(nc2)Nc(c(c3)OC)ccc3C(C4)CN4c(ccc5)c(c56)C(=O)N(C6=O)[C@@H](CC7)C(=O)NC7=O	C35H33Cl1N7O6P1	113 ± 16	1043 ± 96	108	77
9k	c1cccc(P(=O)(C)C)c1Nc(c2Cl)nc(nc2)Nc(c(c3)OC)ccc3CC4CCN(CC4)c(ccc5)c(c56)C(=O)N(C6=O)[C@@H](CC7)C(=O)NC7=O	C38H39Cl1N7O6P1	11.2 ± 1.3	87 ± 3	6	99

9l	c1cccc(P(=O)(C)C)c1Nc(c2Cl)nc(nc2)Nc(c(c3)OC)ccc3N4CCN(CC4)c(cc5)cc(c56)CN(C6=O)[C@@H](CC7)C(=O)NC7=O	C36H38Cl1N8O5P1	6.6 ± 0.4	427 ± 32	8	92
9m	c1cccc(P(=O)(C)C)c1Nc(c2Cl)nc(nc2)Nc(c(c3)OC)ccc3N4CCN(CC4)CC(C5)CN5c(cc6)cc(c67)CN(C7=O)[C@@H](CC8)C(=O)NC8=O	C40H45Cl1N9O5P1	8 ± 0.9	120 ± 27	9	94
9n	c1cccc(P(=O)(C)C)c1Nc(c2Cl)nc(nc2)Nc(c(c3)OC)ccc3N4CCN(CC4)CC(CC5)CCN5c(cc6)cc(c67)CN(C7=O)[C@@H](CC8)C(=O)NC8=O	C42H49Cl1N9O5P1	9.5 ± 0.5	195 ± 124	11	93
9o	c1cccc(P(=O)(C)C)c1Nc(c2Cl)nc(nc2)Nc(c(c3)OC)ccc3N4CCC(CC4)N5CCN(CC5)CC(CC6)CCN6c(cc7)cc(c78)CN(C8=O)[C@@H](CC9)C(=O)NC9=O	C47H58Cl1N10O5P1	1.1 ± 0.3	6.3 ± 2.1	0.8	99
9p	c1cccc(P(=O)(C)C)c1Nc(c2Cl)nc(nc2)Nc(c(c3)OC)ccc3N4CCC(CC4)N5CCN(CC5)CC(C6)CN6c(cc7)cc(c78)CN(C8=O)[C@@H](CC9)C(=O)NC9=O	C45H54Cl1N10O5P1	15.3 ± 4.5	158 ± 81	20	94
9q	c1cccc(P(=O)(C)C)c1Nc(c2Cl)nc(nc2)Nc(c(c3)OC)ccc3N4CCC(CC4)N5CCN(CC5)CC(CC6)CCN6c(cc7)ccc7N[C@@H](CC8)C(=O)NC8=O	C45H58Cl1N10O4P1	102 ± 24	>10000	145	33
9r	c1cccc(P(=O)(C)C)c1Nc(c2Cl)nc(nc2)Nc(c(c3)OC)ccc3N4CCC(CC4)N5CCN(CC5)CC(CC6)CCN6c(cc7)ccc7C(=O)N[C@@H](CC8)C(=O)NC8=O	C46H58Cl1N10O5P1	120 ± 39	>10000	123	28
9s	c1cccc(P(=O)(C)C)c1Nc(c2Cl)nc(nc2)Nc(c(c3)OC)ccc3N4CCC(CC4)N5CCN(CC5)C(=O)CN(CC6)CCN6c(cc7)ccc7N[C@@H](CC8)C(=O)NC8=O	C45H57Cl1N11O5P1	75 ± 9	>10000	84	40
9t	c1cccc(P(=O)(C)C)c1Nc(c2Cl)nc(nc2)Nc(c(c3)OC)ccc3N4CCC(CC4)N5CCN(CC5)CCC(CC6)CCN6c(cc7)ccc7N[C@@H](CC8)C(=O)NC8=O	C46H60Cl1N10O4P1	61 ± 3	>10000	56	45
9u	c1cccc(P(=O)(C)C)c1Nc(c2Cl)nc(nc2)Nc(c(c3)OC)ccc3N4CCC(CC4)N5CCN(CC5)C(=O)CC(CC6)CCN6c(cc7)ccc7N[C@@H](CC8)C(=O)NC8=O	C46H58Cl1N10O5P1	82 ± 9	>10000	101	37
9v	c1cccc(P(=O)(C)C)c1Nc(c2Cl)nc(nc2)Nc(c(c3)OC)ccc3N4CCC(CC4)N5CCN(CC5)C(=O)C(CC6)CCN6c(cc7)ccc7N[C@@H](CC8)C(=O)NC8=O	C45H56Cl1N10O5P1	63 ± 2.4	>10000	76	42
